# Genetically determined blood pressure, antihypertensive medications, and risk of Alzheimer’s disease: a Mendelian randomization study

**DOI:** 10.1186/s13195-021-00782-y

**Published:** 2021-02-09

**Authors:** Ya-Nan Ou, Yu-Xiang Yang, Xue-Ning Shen, Ya-Hui Ma, Shi-Dong Chen, Qiang Dong, Lan Tan, Jin-Tai Yu

**Affiliations:** 1 Department of Neurology, Qingdao Municipal Hospital, Qingdao University, Qingdao, 266071 China; 2 Department of Neurology and Institute of Neurology, WHO Collaborating Center for Research and Training in Neurosciences, Huashan Hospital, Shanghai Medical College, Fudan University, Shanghai, China

**Keywords:** Blood pressure, Antihypertensive medications, Alzheimer’s disease, Single nucleotide polymorphism, Mendelian randomization

## Abstract

**Background:**

Observational studies suggest that the use of antihypertensive medications (AHMs) is associated with a reduced risk of Alzheimer’s disease (AD); however, these findings may be biased by confounding and reverse causality. We aimed to explore the effects of blood pressure (BP) and lowering systolic BP (SBP) via the protein targets of different AHMs on AD through a two-sample Mendelian randomization (MR) approach.

**Methods:**

Genetic proxies from genome-wide association studies of BP traits and BP-lowering variants in genes encoding AHM targets were extracted. Estimates were calculated by inverse-variance weighted method as the main model. MR Egger regression and leave-one-out analysis were performed to identify potential violations.

**Results:**

There was limited evidence that genetically predicted SBP/diastolic BP level affected AD risk based on 400/398 single nucleotide polymorphisms (SNPs), respectively (all *P* > 0.05). Suitable genetic variants for β-blockers (1 SNP), angiotensin receptor blockers (1 SNP), calcium channel blockers (CCBs, 45 SNPs), and thiazide diuretics (5 SNPs) were identified. Genetic proxies for CCB [odds ratio (OR) = 0.959, 95% confidence interval (CI) = 0.941–0.977, *P* = 3.92 × 10^−6^] and overall use of AHMs (OR = 0.961, 95% CI = 0.944–0.978, *P* = 5.74 × 10^−6^, SNPs = 52) were associated with a lower risk of AD. No notable heterogeneity and directional pleiotropy were identified (all *P* > 0.05). Additional analyses partly support these results. No single SNP was driving the observed effects.

**Conclusions:**

This MR analysis found evidence that genetically determined lowering BP was associated with a lower risk of AD and CCB was identified as a promising strategy for AD prevention.

**Supplementary Information:**

The online version contains supplementary material available at 10.1186/s13195-021-00782-y.

## Background

Alzheimer’s disease (AD) prevalence is rising, further increasing the social and economic burden [1]. In the absence of any therapeutic intervention, prevention strategies that target modifiable risk factors are promising approaches. Hypertension has emerged as a potential risk factor for AD [2, 3]. Antihypertensive medications (AHMs) have also been highlighted as priority repurposing candidates for AD prevention [4, 5]. However, inference from observational studies is limited by residual confounding, reverse causation, and detection bias [6]. Difficulties in implementing large-scale randomized clinical trials (RCTs) also restrict the exploration of this association.

A novel method for estimating causal effects of risk factors in observational studies using genetic variants is Mendelian randomization (MR) [7]. Due to the random assortment of genes at conception, MR overcomes the core shortcomings of observational studies and assesses lifelong exposures to risk factors, and thus, it can clarify potential causal associations [6]. Recently, two studies estimated causal effects of BP level on AD risk using MR analysis. One employed 24 single nucleotide polymorphisms (SNPs) indicating that genetically predicted higher systolic blood pressure (SBP) was causally associated with a lower risk of AD [8]. However, another study exploited more SNPs (*N* = 93), finding no significant association between SBP level and AD [9]. Andrews et al. also found a null association between polygenic risk score (PRS, prioritizing putative causal risk factor score) for increased SBP and AD risk [10]. The differences among the research results may lie in the differences in the number of SNPs included, the statistical power, and analytical bias; thus, higher-quality studies with larger sample size are urgently needed to corroborate this question. Furthermore, the expression and function of drug targets can be influenced by variants within or near the genes that encode them. Therefore, the effects of drug action can be anticipated by the genetic effects in the genes of their protein targets, as has previously been applied to lipid-lowering drugs [11]. However, only one previous study employed MR approach to investigate the effect of AHMs on AD risk, suggesting that lowering SBP via the protein targets of AHMs is unlikely to affect the risk of developing AD [12].

Herein, considering that previous MR analyses have yielded opposite results and larger databases of BP traits and AD are now available, we aimed to perform a two-sample MR analysis to assess the causal effects of genetically determined BP and genetic proxies for antihypertensive drug classes on the risk of AD comprehensively.

## Methods

### Instrument identification

Significant SNPs (*P* < 5 × 10^−8^) for BP were identified from a genome-wide association study (GWAS) meta-analysis that included 757,601 individuals of European ancestry drawn from UK Biobank (UKB, *N* = 458,577) and the International Consortium of Blood Pressure (ICBP, *N* = 299,024) database [13]. In UKB, two BP measurements were taken after a 2-min rest using an Omron HEM-7015IT digital BP monitor, or a manual sphygmometer. The mean SBP and diastolic BP (DBP) values were calculated from two automated or two manual BP measurements. For individuals with one manual and one automated BP measurement, the mean of these two values were used. For individuals with only one available BP measurement, this single value was used. The mean age of participants ranged from 56.8 to 62.1 years old (see Additional file 1).

**Fig. 1:**
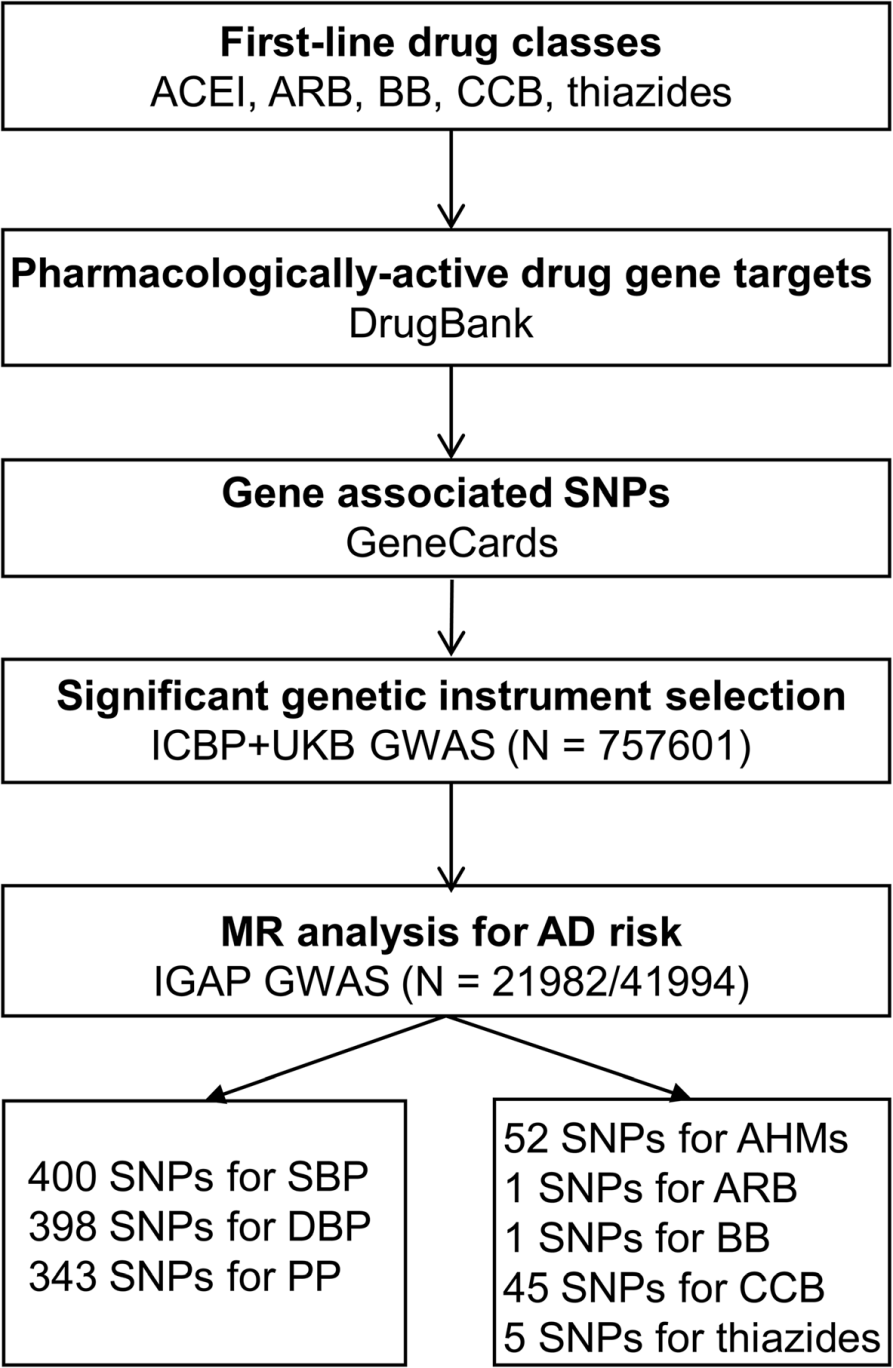
Flow diagram of the process for identifying genetic variants. Abbreviations: ACEI, angiotensin converting enzyme inhibitors; ARB, angiotensin receptor blockers; BB, β-blockers, CCB, calcium channel blocker; SNP, single nucleotide polymorphism; ICBP, International Consortium for Blood Pressure; UKB, UK Biobank; GWAS, genome-wide association studies; SBP, systolic blood pressure; DBP, diastolic blood pressure; PP, pulse pressure; MR, Mendelian randomization; AD, Alzheimer’s disease; AHMs, antihypertensive medications

We selected genetic variants as proxies for the SBP lowering effects of common antihypertensive drug classes: angiotensin converting enzyme inhibitors (ACEI), angiotensin receptor blockers (ARB), β-blockers (BB), calcium channel blockers (CCB), and thiazide diuretic agents on the basis of new consensus guidelines (Fig. 1) [14]. We identified the genes encoding pharmacologic targets related to BP lowering for common antihypertensive drug classes in DrugBank (https://www.drugbank.ca/) [15] and screened the genomic SNPs corresponding to these genes in GeneCards (https://www.genecards.org/) [16]. From all the identified variants in each gene, only variants that are significantly associated with SBP (*P* < 5 × 10^−8^) and clumped to a linkage disequilibrium (LD) threshold of *R*^2^ < 0.4 using the 1000G European reference panel were considered as candidate proxies for each medication class. This relatively lenient LD correlation threshold allows for an increase in proportion of variance explained and thus in statistical power [17–19]. For additional analysis, we also employed more stringent LD thresholds (*R*^2^ < 0.1 and *R*^2^ < 0.001, respectively).

### AD database

AD data were from a large famous GWAS dataset from International Genomics of Alzheimer’s Project (IGAP) GWAS Stage 1 result (*N* = 21,982 cases, 41,944 controls) [20]. It is composed of datasets from the Alzheimer Disease Genetics Consortium (ADGC), Cohorts for Heart and Aging Research in Genomic Epidemiology Consortium (CHARGE), The European Alzheimer’s Disease Initiative (EADI), and Genetic and Environmental Risk in AD/Defining Genetic, Polygenic and Environmental Risk for Alzheimer’s Disease Consortium (GERAD/PERADES). AD cases were all autopsy-confirmed or clinically confirmed using published criteria. The mean age of onset of AD cases ranged from 71.1 to 82.6 years, and the mean age of onset of healthy controls ranged from 51.0 to 78.9 years **(**see Additional file 1**)**.

### Statistical analyses

This MR approach was based on 3 assumptions: (1) the genetic variants associate with the exposure, (2) the instrumental variables (IVs) have no association with confounding factors, and (3) the risk of AD is influenced only by the exposure, not by other pathways [7, 21]. We estimated the overall effect of SBP/DBP/pulse pressure (PP) on AD by combining the effects of all the genome-wide significant SNPs (*P* < 5 × 10^−8^) from the UKB+ICBP GWAS which were then clumped based on the European 1000 Genomes panel to *R*^2^ < 0.001. SNPs absent in the outcome data were replaced by proxy SNPs in high LD from the 1000 Genomes Project European data where possible. Proxies were required to have a minimum *R*^2^ value of 0.8 and palindromic SNP strands were aligned using minor allele frequency up to 0.3 [22]. We excluded the SNPs that had *F* statistics lower than 10 according to standard practice [23]. Then, we estimated the effects of AHMs on AD by selecting SNPs associated with AHMs at genome-wide significance (*P* < 5 × 10^−8^) that were at moderate to low LD (*R*^2^ < 0.4) and more stringent LD thresholds (*R*^2^ < 0.1 and *R*^2^ < 0.001). The genes and the specific genomic regions screened for the identification of genetic proxies for each AHM class were detailed (see Additional file 2).

Causal effects were estimated with the random-effects maximum likelihood estimation method. We applied five complementary methods [inverse variance weighted (IVW), MR-Egger, weighted median, simple mode, and weighted mode], which provided different assumptions about horizontal pleiotropy [24]. The IVW method was performed as our primary method, which essentially assumed the intercept was zero and associated a weighted regression of SNP-exposure effects with SNP-outcome effects. The weighted median approaches give more weight to more precise instrumental variables and the estimate is consistent even when up to 50% of the information comes from invalid or weak instruments [7]. The association is considered significant after the correction for multiple testing for three BP indexes [*P* < 0.016 (0.05/3)] and five AHM classes [*P* < 0.01 (0.05/5)]. A *P* value above 0.016/0.01 but below 0.05 was considered suggestive of evidence for a potential association. Results were presented as odds ratios (ORs) and 95% confidence intervals (CIs) of AD per genetically predicted unit log-transformed increase in each trait. We estimated the intercept of MR-Egger regression, which represented the average horizontal pleiotropy [25]. We conducted a leave-one-SNP-out analysis in which we systematically removed one SNP at a time to assess the influence of potentially pleiotropic SNPs on the causal estimates. The strength of the genetic instrument was judged with *F* statistics [23]. *F *statistics are greater than 10, indicating that the instrument strength was sufficient for MR analysis [26]. Statistical significance of the above analyses was set at a 2-sided *P* value < 0.05. Statistical analyses were conducted in R (version 3.5.3) and MR analyses were conducted using “TwoSampleMR”.

## Results

### Genetically determined BP and risk of AD

**Fig. 2:**
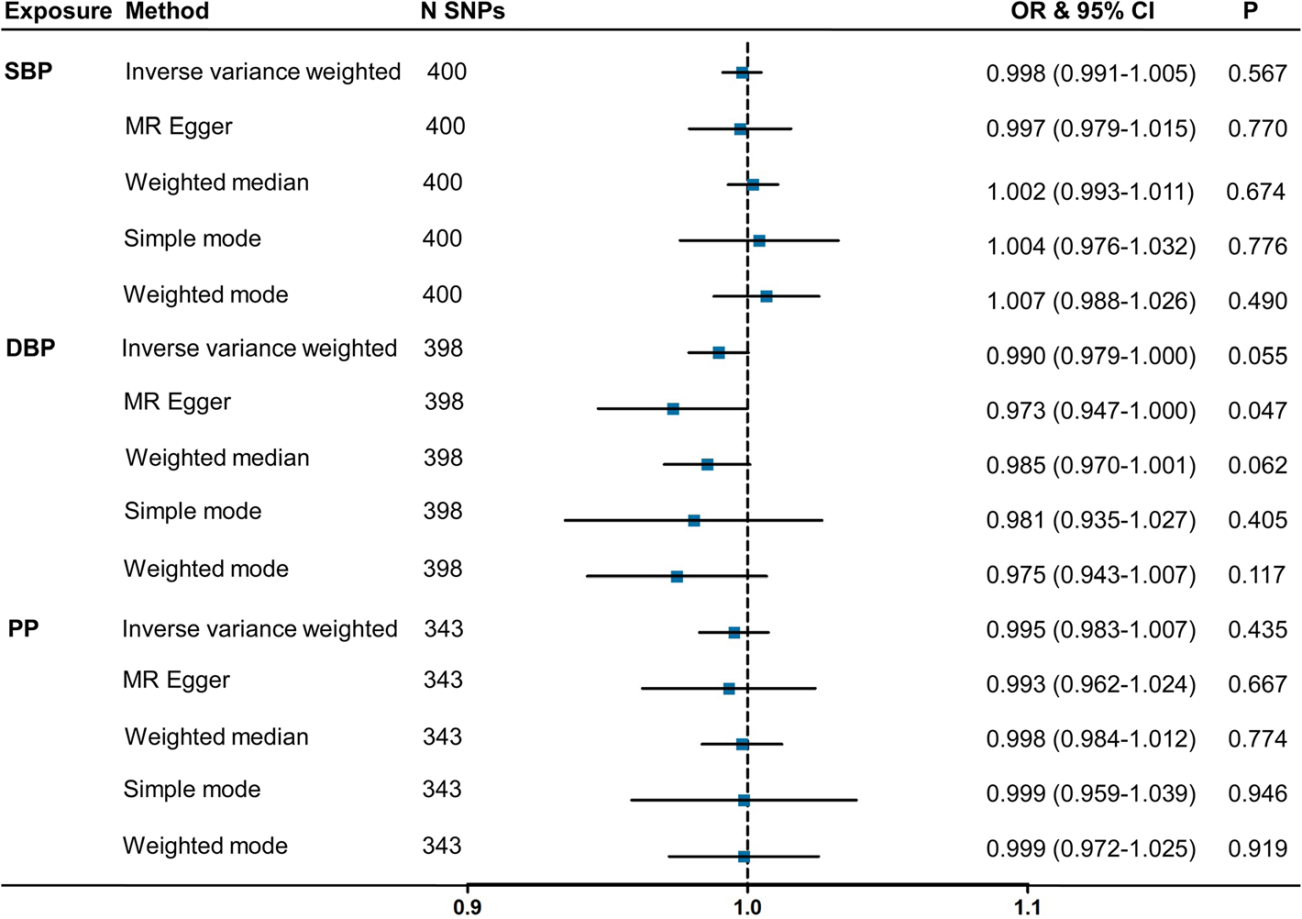
MR associations between genetically determined blood pressure and the risk of AD. Genome-wide significantly associated (*P* < 5 × 10^−8^) independent (LD *R*^2^ = 0.001, clumping distance = 10,000 kb) SNPs were used as instruments. Abbreviations: MR, Mendelian randomization; SNP, single nucleotide polymorphism; OR, odds ratio; CI, confidence interval; SBP, systolic blood pressure; DBP, diastolic blood pressure; PP, pulse pressure; LD, linkage disequilibrium

First, we examined the relationship between genetically determined BP and the risk of AD. A total of 400/398/343 independent genetic variants were found to be associated with SBP/DBP/PP, respectively (see Additional files 3, 4, 5). There was no evidence of an association between either genetically predicted SBP or PP with AD, with *P* values > 0.05 in all of the analyses (Fig. 2). However, the results were suggestive of an association between DBP and AD using IVW method with an OR of 0.990 (95% CI = 0.979–1.000, *P* = 0.055). The sensitivity analyses, MR Egger (OR = 0.973, 95% CI = 0.947–1.000, *P* = 0.047), and weighted median (OR = 0.985, 95% CI = 0.970–1.001, *P* = 0.062) methods confirmed these results. There was evidence of heterogeneity in the causal effect estimates from all of the MR analyses (all *P* values < 0.05, Table 1). Nonetheless, horizontal pleiotropic effects were absent in MR Egger regression (intercept 0.003, *P* = 0.935 for SBP; intercept 0.002, *P* = 0.182 for DBP; intercept 0.003, *P* = 0.897 for PP; Table 1). We did not find a single genetic variant of BP that had an influence on the association in the leave-one-out analysis.

**Table 1:** Heterogeneity and pleiotropy tests of instrument effects

Exposure	N SNPs	Heterogeneity analysis				Pleiotropy analysis		
		Method	Q	Degree of freedom	P	Egger intercept	SE	P
**CCB**	45	MR Egger	26.07	43	0.981	− 2.19 × 10^−3^	0.009	0.806
		IVW	26.13	44	0.985			
**Thiazides**	5	MR Egger	2.55	3	0.466	− 4.42 × 10^−2^	0.041	0.350
		IVW	3.77	4	0.438			
**AHMs**	52	MR Egger	31.73	50	0.980	− 4.54 × 10^−3^	0.009	0.586
		IVW	32.03	51	0.983			
**SBP**	400	MR Egger	635.41	398	3.506 × 10^−13^	2.27 × 10^−4^	0.003	0.935
		IVW	635.42	399	4.441 × 10^−13^			
**DBP**	398	MR Egger	524.27	396	1.557 × 10^−5^	3.25 × 10^−3^	0.002	0.182
		IVW	526.63	397	1.330 × 10^−5^			
**PP**	343	MR Egger	699.38	341	6.704 × 10^−27^	4.24 × 10^−4^	0.003	0.897
		IVW	699.41	342	9.550 × 10^−27^			

*Abbreviations*: *MR* Mendelian randomization, *IVW* inverse variance weighted, *CCB* calcium channel blocker, *AHMs* antihypertensive medications, *SBP* systolic blood pressure, *DBP* diastolic blood pressure, *PP* pulse pressure, *SE* standard error

### Genetic proxies for antihypertensive drugs and risk of AD

Next, we selected BP-lowering variants in genes encoding drug targets as proxies for the effects of AHM classes and examined their effects on AD. We identified a total of 52 variants for AHMs, including 1 for ARB, 1 for BB, 45 for CCB, and 5 for thiazides. However, we failed to explore the casual effects of ACEI on AD risk because no proxy was identified. All SNPs had *F* values > 10, suggesting that they were unlikely to introduce marked weak instrument bias into the MR analyses (see Additional file 6).

**Fig. 3:**
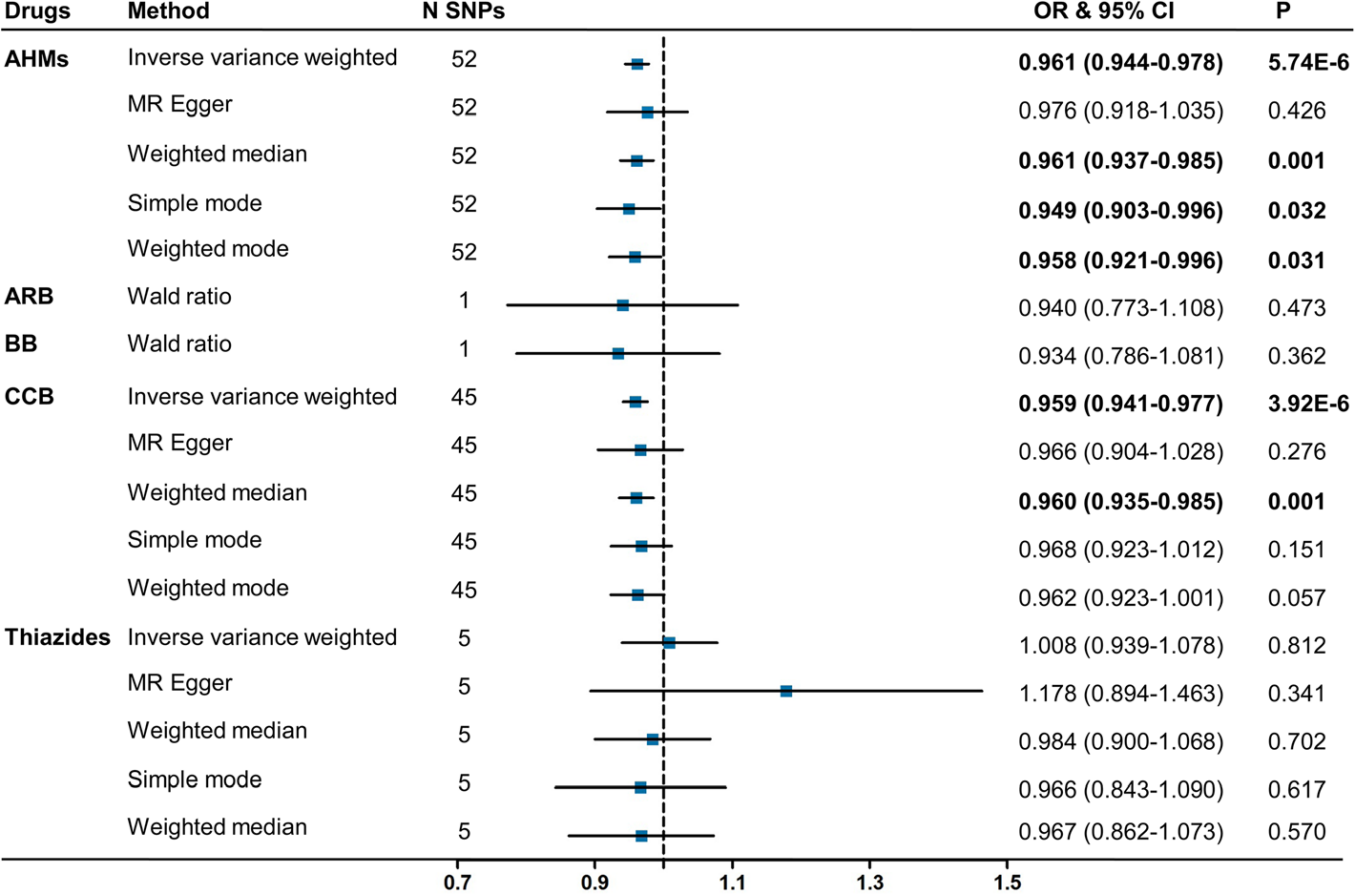
MR associations between genetic proxies for antihypertensive drug classes and the risk of AD. Genome-wide significantly associated (*P* < 5 × 10^−8^) independent (LD *R*^2^ < 0.4) SNPs were used as instruments. Bold fonts indicate significant associations. Abbreviations: SNP, single nucleotide polymorphism; OR, odds ratio; CI, confidence interval; AHMs, antihypertensive medications; ARB, angiotensin receptor blockers; BB, β-blockers; CCB, calcium channel blocker; LD, linkage disequilibrium

The MR analysis showed an association of the overall use of AHMs with lower risk of AD (OR = 0.961, 95% CI = 0.944–0.978, *P* = 5.74 × 10^−6^, SNPs = 52; Fig. 3). The associations were confirmed using sensitivity analyses including the methods of weighted median (OR = 0.961, 95% CI = 0.937–0.985, *P* = 0.001), simple mode (OR = 0.949, 95% CI = 0.903–0.996, *P* = 0.032), and weighted mode (OR = 0.958, 95% CI = 0.921–0.996, *P* = 0.031). The Cochran Q statistic of IVW method (Q = 26.130; *P* = 0.985) indicated no notable heterogeneity across instrument SNP effects (Table 1). Egger analysis did not show evidence of directional pleiotropy (*P* > 0.05). There was no distortion in the leave-one-out plot, suggesting that no single SNP was driving the observed effect in any analysis (see Additional file 7).

Reduction in SBP through variants in genes encoding targets of CCB was associated with a lower risk of AD (OR = 0.959, 95% CI = 0.941–0.977, *P* = 3.92 × 10^−6^, SNPs = 45; Fig. 3). The association was confirmed by sensitivity analyses using weighted median method (OR = 0.960, 95% CI = 0.935–0.985, *P* = 0.001). No evidence of heterogeneity and pleiotropy in the causal effect estimates was found (all *P* > 0.05, Table 1). Leave-one-out analysis did not change the overall direction (see Additional file 7). However, we did not find evidence of causal effects of ARB, BB, and thiazides on the risk of AD.

### Results of additional analysis

Additional analysis restricted to the set of SNPs with the LD threshold (*R*^2^ < 0.1) showed consistent association estimates with an OR of 0.968 (95% CI = 0.942–0.994, *P* = 0.003, SNPs = 27; see Additional file 8) for the overall AHMs. However, none of the sensitivity analyses were significant, including weighted median method with *P* = 0.078. Then, corresponding estimates using *R*^2^ < 0.001 were also presented, and estimates were partly consistent with a borderline significant OR_WM_ of 0.951 (*P* = 0.063, SNPs = 9; see Additional file 9). As for CCB, the results were consistent using LD *R*^2^ < 0.1 with 21 SNPs included, resulting in an OR of 0.955 (95% CI = 0.927–0.983, *P* = 0.002). Weighted median analysis failed to support the positive association with *P* = 0.099. When the more stringent pairwise threshold (*R*^2^ < 0.001) was used to construct the aggregated IVs, the results were partly concordant with the main observations with *P*_WM_ = 0.096. These relationships were again not robust, thus highlighting the need for caution in interpreting these effects as causal.

## Discussion

This two-sample MR study, in which we used genetic variants as proxies associated with BP in a very large cohort of well-characterized research participants, provided suggestive evidence for associations between genetically exposure to BP lowering through AHMs and a reduced risk of AD and further identified CCB as a promising strategy for AD prevention. However, these results should be interpreted with great caution.

Hypertension has been implicated as a risk factor for AD [3]. However, uncertainties remain over the nature of the association, which perhaps is complicated by misclassification of different forms of dementia, or the age of study participants [2, 27]. Several studies suggested that high BP in midlife was associated with a higher risk of AD, whereas other studies indicated that high BP in late life might be protective against AD [2, 27, 28]. Large-scale biobank datasets can provide an unparalleled opportunity to undertake hypothesis-free causal inference. Using a MR approach, the current study failed to identify a causal relationship between SBP level and AD risk. Previous observational studies have produced a consistent finding of no association between high blood pressure and AD in late-life [28]. There are two earlier studies using MR to evaluate the association of SBP with AD cases-control status. Østergaard and colleagues observed that higher SBP was associated with a reduced risk of AD [8]. However, Larsson and colleagues exploited more SNPs, finding no significant association between SBP level and AD [9]. Andrews and colleagues also found a null association between PRS for increased SBP and AD risk [10].

The association of DBP level with clinically diagnosed AD has not been extensively studied, though several studies have conducted phenome-wide scans. Using data from the UK Biobank, Richardson and colleagues found that a higher AD PRS was associated with lower DBP [29]. Similarly, a second study by Korologou-Linden and colleagues evaluated the association of an AD PRS composed of 18 SNPs, inclusive of *APOE*, finding that a higher AD PRS was associated with lower DBP [30]. This present result was also suggestive of an association between high DBP level and a lower risk of AD. High BP in late life might be protective against AD. Consistent with this, one study also showed a greater effect of decreased DBP on white matter hyperintensity volume (WMHV) burden, particularly among those who previously had a greater increase in SBP [31]. Hypotension in late life might aggravate cerebral small vessel disease and decrease brain volume in cognitively normal individuals, potentially via shifts in the auto-regulatory curve and resultant cerebral hypoperfusion [32, 33]. Alternatively, this finding might be mediated by increased arterial stiffness, which is associated with decreased DBP, although we did not find a direct association between PP, a proxy marker of arterial stiffness, and AD risk. One recent MR analysis observed association between high BP and vascular brain injury (VBI), which suggests that while reducing BP in late life may have limited utility in the prevention of AD, it may reduce the risk of vascular dementia by reducing the risk of VBI, but not specifically affect the risk of AD [10].

**Fig. 4:**
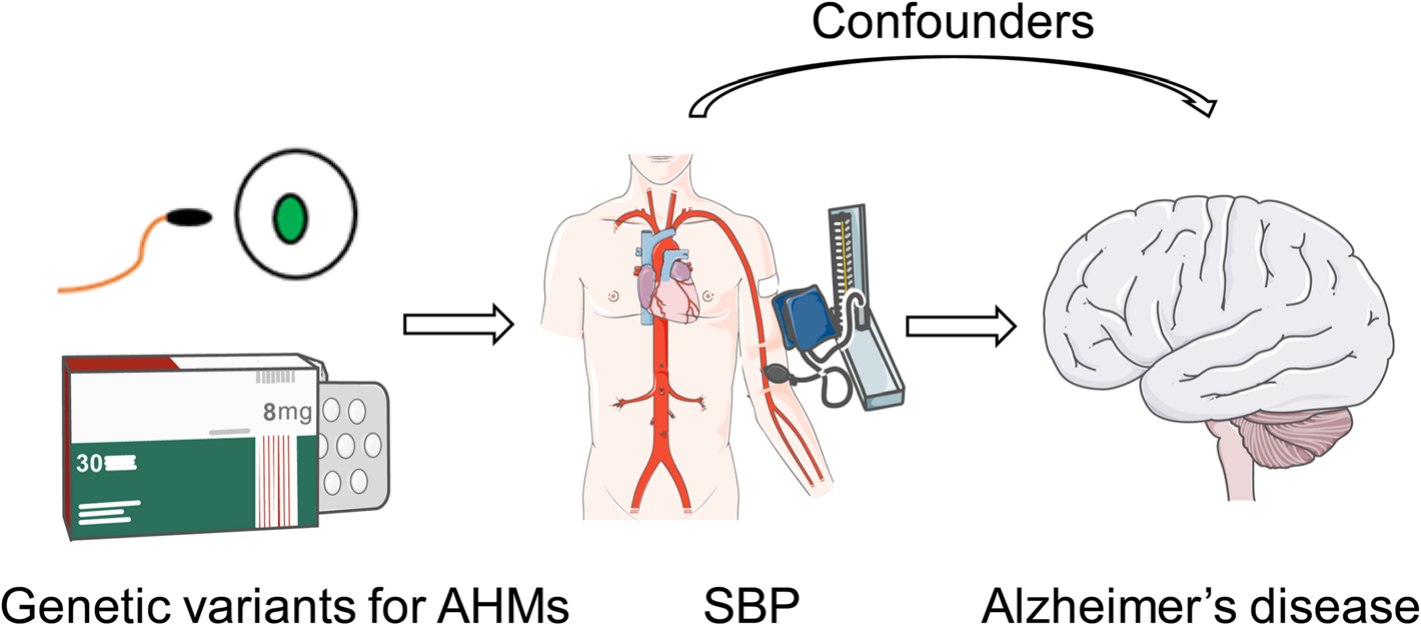
Conceptual framework for the MR analysis of AHMs and risk of AD. Abbreviations: SBP, systolic blood pressure; AHMs, antihypertensive medications

There is also a wealth of evidence in the literature from observational studies indicating that antihypertensive therapy may protect against AD or delay its onset [4, 5]. A recent high-quality meta-analysis found that among people with high BP, the use of AHMs might reduce the risk of AD [4]. A British cohort study of dementia-free individuals concluded that BP monitoring and interventions need to start around 40 years of age to preserve cognition in older age [31]. However, the well-known SPRINT MIND study did not find any significant difference in the risk of dementia between intensive and standard BP control [34]. The study may have been underpowered for this end point due to early study termination and fewer than expected cases of dementia. Using MR, the current study extends previous evidence using genetic variants in a very large cohort of well-characterized research participants and then selected gene targets of AHMs, showing that genetically determined lowering BP through AHMs was associated with a lower risk of AD, and CCB was identified as a promising strategy for AD prevention (we depicted a schematic diagram of mechanism here, see Fig. 4). One RCT found beneficial cerebrovascular effects of calcium antagonists on AD [35]. However, one recent MR analysis have showed that lowering SBP via AHMs is unlikely to affect the risk of developing AD, and if specific AHM classes do reduce the risk of AD, the mechanism may not be via SBP pathway [12]. Actually, studies have pointed out that some drugs, acting through calcium channel blocking mechanisms, have protective effects on AD, independently of BP lowering [36]. For example, the intracellular buildup of calcium in neurons can be neurotoxic and thus CCB might result in neuroprotection [37]. Although, the underlying mechanism mediating the protective effects of AHMs on AD remains unclear and warrants further research, MR analyses surely hold huge promise in the era of large-scale genetic epidemiology to identify risk or protective factors. Associations detected between AHMs (including CCB) and AD risk undertaken by large-scale analyses should prove powerful for future studies that wish to unravel causal relationships between complex traits [29].

### Limitations

The results of this study should be interpreted in conjunction with some limitations. First, we used genetic variants derived from a study with a relatively large sample size which were strongly associated with BP to avoid weak instrument problems, but our finding may still be affected by weak instrument bias [26]. Second, we were limited by the fact that MR explores the effect of lifelong exposure, whereas drugs typically have much shorter periods of exposure and BP may have age-dependent effects. The effect sizes that we have estimated will not represent the associations between critical periods of exposure and the outcome [38]. This can also be particularly problematic if the protein target of a drug is beneficial at one point during the life course and harmful at another. Thus, further work, especially RCTs, is recommended to investigate the pathways from BP/AHMs to AD and to explore how the effect varies with age. Third, as drug target models only focus on-target effects of the specific therapeutics, our genetic results for drug targets cannot reflect the pharmacokinetics of drug use. Thus, the associations between the drugs and the outcome cannot be fully reflected by the present analysis. Fourth, though we chose a liberal LD clumping threshold (*R*^2^ < 0.4) when selecting the variants associated with AHMs according to previously published approaches, this threshold introduced several dependent variants. We further employed more stringent thresholds (*R*^2^ < 0.1 and *R*^2^ < 0.001); these results inferred the possibility that a single locus with multiple SNPs might partly drive the association. Therefore, the positive associations of AHMs, including CCB with reduced AD risk, should be interpreted with great caution. Fifth, we failed to explore the protective effects of other AHM classes, including ACEI, ARB, BB, and thiazide diuretics. These null results did not mean that there were no protective effects of these medications, given that the limited number of included SNPs failed to offer sufficient statistical power to perform meaningful analyses. Future studies encompassing larger GWAS datasets for BP might identify more variants and offer deeper insights into the effects of different classes of BP lowering agents on AD. Sixth, the estimated effect of BP level on AD risk, which is associated with a high risk of mortality, may be susceptible to survival bias. Last, since all of participants are of European ancestry, the results of this study are not necessarily valid for other ethnic groups.

## Conclusions

In this two-sample MR study, we examined the effects of BP level and lowering BP via AHMs on AD using genetic variants. We provided evidence that inherited exposure to BP lowering through AHMs was associated with lower a risk of AD, and CCB might be a promising strategy for AD prevention. Evidence showed that there is an effect of AHMs on AD, which may be partly mediated by the mechanism of the BP-lowering effect. Our results complement the findings from observational studies and warrant further investigation for the development of potential AD preventive strategies targeting BP control.

## Supplementary Information

**Additional file 1.** Demographic characteristics of included GWASes used in the present MR analysis.

**Additional file 2.** Drug classes, substances and targets with their DrugBank ID.

**Additional file 3.** Genome-wide significant and independent SNPs that were used as instruments for SBP.

**Additional file 4.** Genome-wide significant and independent SNPs that were used as instruments for DBP.

**Additional file 5.** Genome-wide significant and independent SNPs that were used as instruments for PP.

**Additional file 6.** SNPs that fulfilled our selection criteria to be used as proxies for the effects for AHM classes.

**Additional file 7.** Leave-one-out plots.

**Additional file 8.** MR results for the casual relationships between AHMs and AD using a LD *R*^2^ < 0.1.

**Additional file 9.** MR results for the casual relationships between AHMs and AD using a LD *R*^2^ < 0.001.

## Data Availability

The analyses for this study were based on publicly available summary statistics. The datasets supporting the conclusions of this article are included within the article and its additional files.

## Abbreviations

AD: Alzheimer’s disease
AHMs: Antihypertensive medications
RCT: Randomized clinical trials
MR: Mendelian randomization
SBP: Systolic blood pressure
SNP: Single nucleotide polymorphisms
GWAS: Genome-wide association study
UKB: UK Biobank
ICBP: International Consortium of Blood Pressure
DBP: Diastolic BP
ACEI: Angiotensin converting enzyme inhibitors
ARB: Angiotensin receptor blockers
BB: β-Blockers
CCB: Calcium channel blockers
LD: Linkage disequilibrium
IGAP: International Genomics of Alzheimer’s Project
ADGC: Alzheimer Disease Genetics Consortium
CHARGE: Cohorts for Heart and Aging Research in Genomic Epidemiology Consortium
EADI: The European Alzheimer’s Disease Initiative
GERAD/PERADES: Genetic and Environmental Risk in AD/Defining Genetic, Polygenic and Environmental Risk for Alzheimer’s Disease Consortium
IV: Instrumental variable
IVW: Inverse variance weighted
OR: Odds ratio
CI: Confidence interval
PRS: Polygenic risk scores
WMHV: White matter hyperintensity volume
VBI: Vascular brain injury
