# Supplementary material for: Genetically determined blood pressure, antihypertensive medications, and risk of Alzheimer’s disease: a Mendelian randomization study
Source: Alzheimers Res Ther. 2021 Feb 9;13:41. doi: 10.1186/s13195-021-00782-y (PMC7874453; doi:10.1186/s13195-021-00782-y)
Supplement: Supplementary file 1 — Additional file 1. Demographic characteristics of included GWASes used in the present MR analysis. [file 13195_2021_782_MOESM1_ESM.docx]

**Additional file 1 Demographic characteristics of included GWASs used in the present MR analysis**

| **GWAS type** | **Sample size (case/control)** | **Mean age of cases** | **Mean age of controls** | **Phenotype ascertainment** |
| --- | --- | --- | --- | --- |
| **AD GWAS** | 21982/41994 |  |  |  |
| ADGC | 14428/14562 | 71.1 | 76.2 | Autopsy-confirmed or clinically-confirmed |
| CHARGE | 2137/13474 | 82.6 | 76.7 | Autopsy-confirmed or clinically-confirmed |
| EADI | 2240/6631 | 75.4 | 78.9 | Autopsy-confirmed or clinically-confirmed |
| GERAD/PERADES | 3177/7277 | 73.0 | 51.0 | Autopsy-confirmed or clinically-confirmed |
| **BP GWAS** | 757601 |  | |  |
| UKB | 458577 | 56.8 | | Digital and manual blood pressure monitor |
| ICBP | 299024 | 62.1 | | Standardized sphygmomanometer |

Abbreviations: GWAS, Genome wide association study; MR, Mendelian randomization; ADGC, Alzheimer Disease Genetics Consortium; CHARGE, Cohorts for Heart and Aging Research in Genomic Epidemiology Consortium; EADI, Genetic and Environmental Risk in AD/Defining Genetic; GERAD/PERADES, Polygenic and Environmental Risk for Alzheimer's Disease Consortium; UKB, UK Biobank; ICBP, International Consortium of Blood Pressure Genome Wide Association Studies.
