# Supplementary material for: Genetically determined blood pressure, antihypertensive medications, and risk of Alzheimer’s disease: a Mendelian randomization study
Source: Alzheimers Res Ther. 2021 Feb 9;13:41. doi: 10.1186/s13195-021-00782-y (PMC7874453; doi:10.1186/s13195-021-00782-y)
Supplement: Supplementary file 2 — Additional file 2. Drug classes, substances and targets with their DrugBank ID. [file 13195_2021_782_MOESM2_ESM.docx]

**Additional file 2 Drug classes, substances and targets with their DrugBank ID**

| **drug** | **substance** | **drugbank_id** | **gene** |
| --- | --- | --- | --- |
| ARB | azilsartan | DB08822 | AGTR1 |
| ARB | candesartan | DB13919 | AGTR1 |
| ARB | eprosartan | DB00876 | AGTR1 |
| ARB | irbesartan | DB01029 | AGTR1 |
| ARB | losartan | DB00678 | AGTR1 |
| ARB | olmesartan | DB00275 | AGTR1 |
| ARB | telmisartan | DB00966 | PPARG;AGTR1 |
| ARB | Candesartan; cilexetil | DB00796 | AGTR1 |
| ARB | Forasartan | DB01342 | AGTR1 |
| ARB | Saprisartan | DB01347 | AGTR1 |
| ARB | Tasosartan | DB01349 | AGTR1;AGTR2 |
| ARB | Fimasartan | DB09279 | AGTR1 |
| ARB | valsartan | DB00177 | AGTR1 |
| ACEI | captopril | DB01197 | ACE |
| ACEI | cilazapril | DB01340 | ACE |
| ACEI | enalapril | DB00584 | ACE |
| ACEI | fosinopril | DB00492 | ACE |
| ACEI | lisinopril | DB00722 | ACE |
| ACEI | moexipril | DB00691 | ACE;ACE2 |
| ACEI | perindopril | DB00790 | ACE |
| ACEI | quinapril | DB00881 | ACE |
| ACEI | ramipril | DB00178 | ACE |
| ACEI | Rescinnamine | DB01180 | ACE |
| ACEI | Benazepril | [DB00542](https://www.drugbank.ca/drugs/DB00542) | ACE |
| ACEI | Enalaprilat | [DB09477](https://www.drugbank.ca/drugs/DB09477) | ACE;BDKRB1 |
| ACEI | Zofenopril | DB13166 | ACE |
| ACEI | Spirapril | DB01348 | ACE |
| ACEI | trandolapril | DB00519 | ACE |
| ACEI | imidapril | DB11783 | ACE |
| BB | atenolol | DB00335 | ADRB1 |
| BB | betaxolol | DB00195 | ADRB1 |
| BB | bisoprolol | DB00612 | ADRB1 |
| BB | Esmolol | DB00187 | ADRB1 |
| BB | Alprenolol | DB00866 | ADRB1;ADRB2;HTR1A;ADRB3 |
| BB | metoprolol | DB00264 | ADRB1 |
| BB | propranolol | DB00571 | ADRB1 |
| BB | timolol | DB00373 | ADRB1;ADRB2 |
| BB | carvedilol | DB01136 | ADRB1;ADRA1A |
| BB | labetalol | DB00598 | ADRA1A;ADRA1D;ADRB2;ADRA1B;ADRB1 |
| BB | Levobunolol | DB01210 | ADRB2;ADRB1 |
| BB | nadolol | DB01203 | ADRB1 |
| BB | oxprenolol | DB01580 | ADRB1 |
| BB | practolol | DB01297 | ADRB1 |
| BB | Metipranolol | DB01214 | ADRB2;ADRB1 |
| BB | Bevantolol | DB01295 | ADRB1;ADRB2;ADRA1A |
| BB | celiprolol | DB04846 | ADRB2;ADRB1 |
| BB | nebivolol | DB04861 | ADRB1 |
| BB | sotalol | DB00489 | KCNH2;ADRB2;ADRB1 |
| BB | Penbutolol | DB01359 | ADRB1;ADRB2;HTR1A;HTR1B |
| BB | Bupranolol | DB08808 | ADRB1;ADRB2;ADRB3 |
| BB | Levobetaxolol | DB09351 | ADRB1 |
| BB | Arotinolol | DB09204 | CYP2D6 |
| BB | carteolol | DB00521 | ADRB1;ADRB2 |
| BB | Sotalol | DB00489 | KCNH2;ADRB1;ADRB2 |
| BB | Arotinolol | DB09204 | ADRB1;ADRB2; |
| BB | Timolol | DB00373 | ADRB1;ADRB2;E |
| BB | Labetalol | DB00598 | ADRB1;ADRB2 |
| BB | Alprenolol | DB00866 | ADRB1;ADRB2;HTR1A;ADRB3 |
| BB | Metipranolol | DB01214 | ADRB2;ADRB1 |
| BB | Penbutolol | DB01359 | ADRB1;ADRB2;HTR1A;HTR1B |
| BB | Levobunolol | DB01210 | ADRB2;ADRB1 |
| BB | acebutolol | DB01193 | ADRB1 |
| BB | carteolol | DB00521 | ADRB1;ADRB2 |
| BB | isoprenaline | DB01064 | ADRB3;ADRB1;ADRB2 |
| BB | pindolol | DB00960 | ADRB1;ADRB2 |
| BB | timolol | DB00373 | ADRB1;ADRB2 |
| CCB | amlodipine | DB00381 | CACNA1D;CACNA1C;CACNB2;CACNA2D1;CACNA1S |
| CCB | isradipine | DB00270 | CACNA1D;CACNA2D2;CACNA2D1;CACNA1S;CACNB2;CACNA1C;CACNA1H |
| CCB | lacidipine | DB09236 | CACNB3;CACNB4;CACNA1S;CACNB2;CACNA1F;CACNA1D;CACNB1;CACNA1C |
| CCB | lercanidipine | DB00528 | CACNG1 |
| CCB | nimodipine | DB00393 | CACNA1S;CACNB2;CACNB4;CACNA1C;CACNA1D;CACNB1;CACNB3;CACNA1F |
| CCB | Benidipine | DB09231 |  |
| CCB | Cilnidipine | DB09232 |  |
| CCB | verapamil | DB00661 | CACNB4;CACNA1S;CACNB1;CACNB3;CACNA1C;CACNB2;CACNA1F;CACNA1D |
| CCB | Aranidipine | DB09229 | CACNA1C;CACNA1D;CACNA1F;CACNA1S |
| CCB | nifedipine | DB01115 | CACNA2D1;CACNA1S;CACNA1C;CACNB2;CACNA1D |
| CCB | nisoldipine | DB00401 | CACNA1S;CACNA1C;CACNB2;CACNA1D;CACNA2D1 |
| CCB | Manidipine | DB09238 |  |
| CCB | Nilvadipine | DB06712 | CACNA1C;CACNA2D1;CACNB2;CACNA1D;CACNA1S;CACNA2D3 |
| CCB | Barnidipine | DB09227 | CACNA1C |
| CCB | Levamlodipine | DB09237 | CACNA1C;CACNA1D; |
| CCB | Diltiazem | DB00343 | CACNG1;CACNA1C |
| CCB | Felodipine | DB01023 | CACNA1C;CACNA2D1;CACNB2;CACNA1D;CACNA1S;CACNA1H;CACNA2D2;PDE1B;PDE1A;NR3C2 |
| CCB | Nitrendipine | DB01054 | CACNA1C;CACNA2D1;CACNB2;CACNA1D;CACNA2D2;CACNA1H;CACNA1S |
| CCB | Nicardipine | DB00622 | CACNA1C;CACNB2;CACNA2D1;CACNA1D;PDE1A;PDE1B;ADRA1A;ADRA1B;ADRA1D;CHRM1;CHRM2;CHRM3;CHRM4;CHRM5 |
| CCB | mibefradil | DB01388 | CACNA1I;CACNB3;CACNB1;CACNA1G;CACNB2;CACNA1C;CACNB4;CACNA1H;CACNA1F;CACNA1D;CACNA1S |
| CCB | perhexiline | DB01074 | CPT2;CPT1A |
| CCB | prenylamine | DB04825 | MYLK2 |
| Thiazides | bendroflumethiazide | DB00436 | SLC12A3;KCNMA1 |
| Thiazides | chlorothiazide | DB00880 | CA1;SLC12A3;CA2 |
| Thiazides | hydrochlorothiazide | DB00999 | SLC12A3;KCNMA1 |
| Thiazides | hydroflumethiazide | DB00774 | SLC12A1 |
| Thiazides | indapamide | DB00808 | SLC12A3 |
| Thiazides | metolazone | DB00524 | SLC12A3 |
| Thiazides | Quinethazone | DB01325 | CA1;CA2;SLC12A1;SLC12A2;SLC12A3 |
| Thiazides | Trichlormethiazide | DB01021 | SLC12A3;ATP1A1;CA1;CA2;CA4 |
| Thiazides | Benzthiazide | DB00562 | SLC12A3;CA1;CA2;CA4;CA9;CA12 |
| Thiazides | chlortalidone | DB00310 | SLC12A1 |
| Thiazides | polythiazide | DB01324 | SLC12A3 |
| Thiazides | Triamterene | DB00384 | SCNN1G;SCNN1A;SCNN1B;SCNN1D |
| Thiazides | Cyclothiazide | DB00606 | FXYD2;CA1;CA2;SFRP4 |
| Thiazides | cyclopenthiazide | DB13532 |  |
| Thiazides | methyclothiazide | DB00232 | SLC12A1 |
| Thiazides | Theobromine | DB01412 | ADORA1;ADORA2A;PDE4B |
| Thiazides | Potassium | DB14500 | ATP1A1 |
| Thiazides | meprobamate | DB00371 | GABRA3;GABRA2;GABRA1;GABRB3;GABRB1;GABRA4;GABRB2;GABRQ;GABRA5;GABRG1;GABRD;GABRG3;GABRG2;GABRA6;GABRP;GABRE |
| Thiazides | Mebutizide | DB13430 |  |
| Thiazides | xipamide |  |  |
| Thiazides | mefruside |  |  |

The latest release of DrugBank (version 5.1.7, released 2020-07-02) contains 13,596 drug entries including 2,640 approved small molecule drugs, 1,389 approved biologics (proteins, peptides, vaccines, and allergenics), 131 nutraceuticals and over 6,377 experimental (discovery-phase) drugs. Additionally, 5,225 non-redundant protein (i.e. drug target/enzyme/transporter/carrier) sequences are linked to these drug entries. Each entry contains more than 200 data fields with half of the information being devoted to drug/chemical data and the other half devoted to drug target or protein data.
