# Supplementary material for: Genetically determined blood pressure, antihypertensive medications, and risk of Alzheimer’s disease: a Mendelian randomization study
Source: Alzheimers Res Ther. 2021 Feb 9;13:41. doi: 10.1186/s13195-021-00782-y (PMC7874453; doi:10.1186/s13195-021-00782-y)
Supplement: Supplementary file 3 — Additional file 3. Genome-wide significant and independent SNPs that were used as instruments for SBP. [file 13195_2021_782_MOESM3_ESM.docx]

**Additional file 3 Genome-wide significant and independent SNPs that were used as instruments for SBP**

| SNP | Beta.exposure | SE.exposure | Pval.exposure | EAF | Effect_allele | Other_allele | Beta.outcome | SE.outcome | Pval.outcome | Samplesize |
| --- | --- | --- | --- | --- | --- | --- | --- | --- | --- | --- |
| rs1000423 | 0.4138 | 0.0346 | 6.50E-33 | 0.2684 | T | C | -0.0033 | 0.0167 | 0.8431 | 757601 |
| rs10043077 | -0.1931 | 0.0324 | 2.52E-09 | 0.361 | T | C | 0.0026 | 0.016 | 0.8692 | 757601 |
| rs10048404 | -0.2607 | 0.0317 | 1.91E-16 | 0.6299 | T | C | 0.0028 | 0.0157 | 0.8587 | 757601 |
| rs10048760 | -0.1862 | 0.0301 | 6.56E-10 | 0.4712 | T | G | -0.0023 | 0.0143 | 0.8705 | 757601 |
| rs1006545 | 0.6846 | 0.048 | 3.50E-46 | 0.1128 | T | G | 0.0045 | 0.0227 | 0.842 | 757601 |
| rs10069690 | 0.3098 | 0.0369 | 4.47E-17 | 0.7418 | T | C | -0.0322 | 0.0215 | 0.1336 | 757601 |
| rs10091532 | -0.2067 | 0.0305 | 1.33E-11 | 0.5832 | A | C | -0.0206 | 0.0145 | 0.1576 | 757601 |
| rs1010064 | 0.3571 | 0.0387 | 3.02E-20 | 0.1837 | A | C | -0.0291 | 0.0182 | 0.11 | 757601 |
| rs10188003 | 0.1883 | 0.0307 | 8.80E-10 | 0.607 | T | C | 0.0075 | 0.0147 | 0.6087 | 757601 |
| rs10207726 | -0.2142 | 0.033 | 8.06E-11 | 0.704 | T | C | 0.0256 | 0.0157 | 0.1041 | 757601 |
| rs10224210 | -0.3831 | 0.034 | 1.60E-29 | 0.2789 | T | C | -0.0029 | 0.0164 | 0.8614 | 757601 |
| rs10282122 | -0.302 | 0.0327 | 2.46E-20 | 0.3316 | T | C | -0.0108 | 0.0156 | 0.4893 | 757601 |
| rs10420519 | -0.4921 | 0.0887 | 2.86E-08 | 0.9653 | T | G | -0.4627 | 0.0539 | 9.14E-18 | 757601 |
| rs1043069 | 0.234 | 0.0311 | 5.26E-14 | 0.3844 | T | G | -0.0145 | 0.0148 | 0.3281 | 757601 |
| rs1044822 | -0.248 | 0.0424 | 5.16E-09 | 0.8518 | T | C | 0.0304 | 0.0202 | 0.1321 | 757601 |
| rs10460108 | 0.2141 | 0.0301 | 1.12E-12 | 0.5199 | A | G | -0.0042 | 0.0144 | 0.7678 | 757601 |
| rs10501122 | 0.1916 | 0.0315 | 1.18E-09 | 0.361 | T | C | 0.025 | 0.0151 | 0.09803 | 757601 |
| rs10501410 | 0.4122 | 0.0607 | 1.10E-11 | 0.9308 | A | G | 0.021 | 0.0294 | 0.4741 | 757601 |
| rs1052501 | 0.2262 | 0.0412 | 4.14E-08 | 0.1671 | T | C | 0.0099 | 0.0189 | 0.5995 | 757601 |
| rs10746963 | -0.2177 | 0.0388 | 2.05E-08 | 0.8166 | A | G | 0.0053 | 0.0189 | 0.7813 | 757601 |
| rs10749572 | -0.203 | 0.0302 | 1.88E-11 | 0.4556 | T | G | 0.0187 | 0.0143 | 0.1913 | 757601 |
| rs10776752 | 0.8211 | 0.0576 | 4.61E-46 | 0.9191 | T | G | -0.0169 | 0.0284 | 0.5519 | 757601 |
| rs10777213 | -0.1786 | 0.0299 | 2.45E-09 | 0.4756 | A | G | 0.0206 | 0.0143 | 0.1485 | 757601 |
| rs1077795 | 0.2507 | 0.0344 | 3.33E-13 | 0.2607 | A | G | -0.0135 | 0.0165 | 0.412 | 757601 |
| rs10782230 | 0.2106 | 0.0302 | 2.91E-12 | 0.5155 | A | G | 0.0221 | 0.0143 | 0.1205 | 757601 |
| rs10804330 | 0.2351 | 0.0306 | 1.62E-14 | 0.4332 | T | C | 0.0073 | 0.0145 | 0.6139 | 757601 |
| rs10866828 | 0.2476 | 0.0355 | 3.19E-12 | 0.7504 | T | C | 0.0232 | 0.0175 | 0.1847 | 757601 |
| rs10941043 | -0.2585 | 0.0332 | 6.42E-15 | 0.2902 | T | G | 0.0059 | 0.0157 | 0.7062 | 757601 |
| rs10980408 | -0.7606 | 0.0827 | 3.83E-20 | 0.0359 | T | C | -0.0382 | 0.0383 | 0.319 | 757601 |
| rs11097909 | -0.3628 | 0.043 | 3.35E-17 | 0.8528 | T | C | 0.0103 | 0.0199 | 0.6044 | 757601 |
| rs11120093 | -0.1792 | 0.0307 | 5.13E-09 | 0.5918 | T | C | 0.0314 | 0.0145 | 0.03027 | 757601 |
| rs11145807 | 0.2135 | 0.0322 | 3.54E-11 | 0.5943 | A | G | -0.0019 | 0.0154 | 0.9002 | 757601 |
| rs11159091 | 0.1978 | 0.0303 | 6.79E-11 | 0.5385 | A | G | -0.0205 | 0.0143 | 0.1516 | 757601 |
| rs111866816 | 0.3569 | 0.0597 | 2.29E-09 | 0.9291 | T | C | -0.0589 | 0.0277 | 0.03363 | 757601 |
| rs11191580 | 1.0995 | 0.055 | 7.74E-89 | 0.0824 | T | C | 0.0229 | 0.0252 | 0.3626 | 757601 |
| rs111929315 | 0.3146 | 0.0485 | 8.60E-11 | 0.1083 | A | G | -0.0164 | 0.0224 | 0.4634 | 757601 |
| rs11210029 | -0.203 | 0.0313 | 8.92E-11 | 0.3678 | A | G | -0.0039 | 0.0148 | 0.7911 | 757601 |
| rs11241313 | -0.2071 | 0.0326 | 2.23E-10 | 0.6888 | T | C | 0.0126 | 0.0154 | 0.4113 | 757601 |
| rs11252324 | -0.4164 | 0.0573 | 3.61E-13 | 0.9229 | T | G | 0.0238 | 0.0259 | 0.3568 | 757601 |
| rs113086489 | 0.3249 | 0.0307 | 3.80E-26 | 0.4475 | T | C | -0.0374 | 0.0147 | 0.01083 | 757601 |
| rs113264678 | 0.4063 | 0.0727 | 2.26E-08 | 0.954 | T | C | -0.0089 | 0.0338 | 0.7915 | 757601 |
| rs1133400 | -0.2975 | 0.0376 | 2.53E-15 | 0.214 | A | G | -0.0456 | 0.0178 | 0.01049 | 757601 |
| rs1154214 | -0.2031 | 0.0306 | 3.27E-11 | 0.6037 | T | G | 0 | 0.0145 | 0.9989 | 757601 |
| rs11592107 | 0.3024 | 0.0326 | 1.55E-20 | 0.6904 | A | G | 0.0098 | 0.0154 | 0.5265 | 757601 |
| rs116025100 | 0.5365 | 0.0851 | 2.86E-10 | 0.9617 | A | G | 0.051 | 0.041 | 0.2138 | 757601 |
| rs11604310 | -0.2778 | 0.0411 | 1.46E-11 | 0.8345 | T | C | 0.0109 | 0.0188 | 0.5639 | 757601 |
| rs11636952 | 0.5313 | 0.0328 | 4.22E-59 | 0.6859 | T | C | -0.0212 | 0.0153 | 0.1673 | 757601 |
| rs11641374 | -0.1943 | 0.0309 | 3.26E-10 | 0.4005 | A | C | -0.0264 | 0.0147 | 0.07192 | 757601 |
| rs11653927 | -0.2796 | 0.0308 | 1.17E-19 | 0.6155 | T | C | -0.0471 | 0.0146 | 0.001232 | 757601 |
| rs11655604 | -0.2033 | 0.0333 | 1.09E-09 | 0.6421 | T | C | -0.0203 | 0.018 | 0.2587 | 757601 |
| rs11672660 | 0.2212 | 0.0381 | 6.32E-09 | 0.8004 | T | C | 0.02 | 0.0178 | 0.2624 | 757601 |
| rs117206641 | 0.3154 | 0.0499 | 2.66E-10 | 0.8892 | T | C | -0.0361 | 0.0239 | 0.1315 | 757601 |
| rs117285318 | 0.4413 | 0.0589 | 6.93E-14 | 0.0775 | T | C | -0.0168 | 0.029 | 0.5617 | 757601 |
| rs117464403 | 0.864 | 0.1199 | 5.80E-13 | 0.9817 | A | G | 0.0067 | 0.0724 | 0.9268 | 757601 |
| rs11874246 | 0.2856 | 0.0328 | 3.23E-18 | 0.7037 | T | C | -0.0055 | 0.0157 | 0.7261 | 757601 |
| rs11925504 | -0.2901 | 0.0305 | 1.78E-21 | 0.4279 | A | G | -0.0169 | 0.0145 | 0.2438 | 757601 |
| rs11960210 | 0.4727 | 0.0313 | 1.25E-51 | 0.3755 | T | C | 0.0148 | 0.0145 | 0.3061 | 757601 |
| rs11977526 | -0.3213 | 0.0312 | 6.62E-25 | 0.5991 | A | G | -0.0029 | 0.0149 | 0.8449 | 757601 |
| rs1199330 | -0.2654 | 0.047 | 1.65E-08 | 0.1176 | A | G | 0.0236 | 0.0225 | 0.295 | 757601 |
| rs12042924 | -0.1807 | 0.0303 | 2.62E-09 | 0.4716 | T | C | -0.0257 | 0.0143 | 0.07152 | 757601 |
| rs12063372 | 0.1989 | 0.0318 | 3.86E-10 | 0.6154 | A | G | 0.0018 | 0.0154 | 0.9045 | 757601 |
| rs1209384 | 0.2558 | 0.0313 | 2.85E-16 | 0.6122 | A | G | 7.00E-04 | 0.015 | 0.9651 | 757601 |
| rs12136922 | 0.2027 | 0.0304 | 2.69E-11 | 0.5051 | A | G | -0.0197 | 0.0141 | 0.1641 | 757601 |
| rs12153395 | -0.3303 | 0.0486 | 1.07E-11 | 0.8853 | A | G | -0.0099 | 0.0244 | 0.6855 | 757601 |
| rs12255372 | 0.2358 | 0.0335 | 1.94E-12 | 0.7117 | T | G | 0.0014 | 0.0156 | 0.9289 | 757601 |
| rs12264186 | 0.2135 | 0.0387 | 3.58E-08 | 0.8129 | T | C | -0.0055 | 0.0184 | 0.763 | 757601 |
| rs12426261 | 0.3775 | 0.0309 | 2.31E-34 | 0.6208 | A | G | -0.0038 | 0.0146 | 0.7966 | 757601 |
| rs12446456 | -0.3003 | 0.0302 | 2.97E-23 | 0.5726 | T | C | -0.0377 | 0.0144 | 0.008659 | 757601 |
| rs12464602 | -0.2437 | 0.0315 | 1.02E-14 | 0.3792 | A | G | -0.0015 | 0.0152 | 0.9199 | 757601 |
| rs12473915 | -0.295 | 0.0375 | 3.42E-15 | 0.7983 | A | G | -0.0158 | 0.0177 | 0.374 | 757601 |
| rs12509595 | -0.8367 | 0.0334 | 2.55E-138 | 0.2923 | T | C | 0.0216 | 0.016 | 0.1766 | 757601 |
| rs12511987 | -0.2329 | 0.0399 | 5.39E-09 | 0.1774 | T | G | 0.0324 | 0.0192 | 0.09069 | 757601 |
| rs12596630 | 0.4278 | 0.0547 | 5.01E-15 | 0.9097 | T | C | -0.021 | 0.0259 | 0.4174 | 757601 |
| rs12627651 | 0.3498 | 0.0341 | 1.02E-24 | 0.7128 | A | G | 0.012 | 0.0166 | 0.4699 | 757601 |
| rs12637573 | -0.1731 | 0.0302 | 9.95E-09 | 0.5282 | A | G | -0.0072 | 0.0143 | 0.6153 | 757601 |
| rs12643599 | 0.3134 | 0.0313 | 1.23E-23 | 0.3605 | A | G | 0.0124 | 0.0148 | 0.4001 | 757601 |
| rs12656497 | -0.6382 | 0.0307 | 7.14E-96 | 0.5966 | T | C | 0.0246 | 0.0145 | 0.09028 | 757601 |
| rs12661036 | -0.2104 | 0.0374 | 1.82E-08 | 0.225 | T | C | 0.0073 | 0.0181 | 0.6852 | 757601 |
| rs12668436 | -0.2151 | 0.035 | 7.88E-10 | 0.2459 | T | C | -0.024 | 0.0165 | 0.1454 | 757601 |
| rs12693982 | 0.2575 | 0.0309 | 7.49E-17 | 0.5976 | T | C | -0.0238 | 0.0148 | 0.1085 | 757601 |
| rs12694277 | -0.2018 | 0.0335 | 1.80E-09 | 0.7054 | T | C | -0.0025 | 0.0157 | 0.8728 | 757601 |
| rs12731646 | -0.189 | 0.0307 | 7.21E-10 | 0.591 | T | C | 0.0035 | 0.0146 | 0.808 | 757601 |
| rs1275988 | -0.541 | 0.0308 | 4.42E-69 | 0.3888 | T | C | -0.0144 | 0.0147 | 0.3281 | 757601 |
| rs12885878 | -0.2291 | 0.0367 | 4.32E-10 | 0.7663 | A | G | -0.0243 | 0.0174 | 0.1616 | 757601 |
| rs12906962 | -0.2653 | 0.0325 | 3.28E-16 | 0.324 | T | C | 0.005 | 0.0156 | 0.7485 | 757601 |
| rs1290784 | 0.4124 | 0.0303 | 2.97E-42 | 0.5517 | T | C | 0.0111 | 0.0143 | 0.4366 | 757601 |
| rs1290933 | -0.2847 | 0.0327 | 3.17E-18 | 0.3081 | A | C | 0.0202 | 0.0157 | 0.1978 | 757601 |
| rs12926550 | -0.2548 | 0.0324 | 3.43E-15 | 0.6844 | A | G | -0.014 | 0.0154 | 0.3631 | 757601 |
| rs1293969 | -0.1988 | 0.0347 | 1.03E-08 | 0.2516 | T | C | -0.0188 | 0.0163 | 0.25 | 757601 |
| rs12985940 | 0.4642 | 0.0434 | 1.08E-26 | 0.1592 | T | C | 0.0373 | 0.0208 | 0.07219 | 757601 |
| rs13016772 | 0.2522 | 0.0355 | 1.23E-12 | 0.2349 | T | C | 0.0462 | 0.0168 | 0.006025 | 757601 |
| rs13107261 | -0.1778 | 0.0314 | 1.57E-08 | 0.6313 | A | G | -0.005 | 0.0148 | 0.7359 | 757601 |
| rs13107325 | -0.9086 | 0.0592 | 4.22E-53 | 0.9261 | T | C | 0.0202 | 0.0273 | 0.4607 | 757601 |
| rs13149209 | 0.281 | 0.0367 | 1.97E-14 | 0.2227 | T | C | 0.0065 | 0.0171 | 0.7022 | 757601 |
| rs13253358 | 0.2127 | 0.033 | 1.13E-10 | 0.7021 | T | C | 0.013 | 0.0156 | 0.4047 | 757601 |
| rs13289468 | 0.2488 | 0.0306 | 3.93E-16 | 0.4257 | A | C | -0.008 | 0.0146 | 0.582 | 757601 |
| rs1332813 | 0.2203 | 0.0314 | 2.32E-12 | 0.6486 | T | C | 0 | 0.0157 | 0.9976 | 757601 |
| rs13358657 | -0.388 | 0.0445 | 2.95E-18 | 0.1332 | A | G | -0.0274 | 0.0213 | 0.1986 | 757601 |
| rs13412750 | -0.2889 | 0.0341 | 2.33E-17 | 0.7292 | A | G | 0.0108 | 0.0161 | 0.5043 | 757601 |
| rs13420463 | 0.3143 | 0.036 | 2.72E-18 | 0.2266 | A | G | -0.0347 | 0.0169 | 0.04048 | 757601 |
| rs1375564 | 0.2579 | 0.0315 | 2.84E-16 | 0.3605 | T | C | -0.0152 | 0.0146 | 0.3 | 757601 |
| rs1382472 | -0.1917 | 0.0307 | 4.47E-10 | 0.5959 | A | G | -0.009 | 0.0146 | 0.5359 | 757601 |
| rs1408945 | -0.3196 | 0.0304 | 8.33E-26 | 0.5757 | T | G | 0.0172 | 0.0144 | 0.2336 | 757601 |
| rs1433121 | -0.228 | 0.0326 | 2.66E-12 | 0.3094 | T | C | 0.0146 | 0.0158 | 0.355 | 757601 |
| rs1436138 | 0.3119 | 0.0315 | 4.73E-23 | 0.3633 | A | G | -0.016 | 0.0151 | 0.2885 | 757601 |
| rs1437649 | -0.2189 | 0.0357 | 8.57E-10 | 0.7655 | A | G | -0.0147 | 0.0169 | 0.384 | 757601 |
| rs145042302 | -0.5886 | 0.0972 | 1.39E-09 | 0.97 | A | G | 0.0026 | 0.0478 | 0.9568 | 757601 |
| rs146550789 | -0.4824 | 0.0778 | 5.64E-10 | 0.0417 | T | C | 0.0484 | 0.0385 | 0.2087 | 757601 |
| rs148140538 | -0.3252 | 0.0562 | 7.39E-09 | 0.9192 | T | C | -0.0639 | 0.0266 | 0.01652 | 757601 |
| rs148401029 | -0.4623 | 0.0848 | 4.97E-08 | 0.9648 | A | C | 0.0369 | 0.0389 | 0.3439 | 757601 |
| rs1493132 | -0.1766 | 0.0318 | 2.73E-08 | 0.3397 | T | C | 0.0298 | 0.0151 | 0.04786 | 757601 |
| rs149339216 | -0.6912 | 0.0779 | 6.93E-19 | 0.0434 | T | C | 0.0675 | 0.0395 | 0.08799 | 757601 |
| rs1551355 | 0.2098 | 0.0356 | 3.89E-09 | 0.7666 | T | C | -0.0275 | 0.017 | 0.1043 | 757601 |
| rs1565440 | 0.1746 | 0.0311 | 1.94E-08 | 0.6248 | A | G | 0.0294 | 0.0147 | 0.04495 | 757601 |
| rs1575290 | 0.1973 | 0.0301 | 5.59E-11 | 0.5267 | T | C | 0.0045 | 0.0143 | 0.7522 | 757601 |
| rs1623474 | 0.3827 | 0.0321 | 7.66E-33 | 0.6697 | T | C | -0.0203 | 0.0151 | 0.1788 | 757601 |
| rs1624823 | 0.3371 | 0.0313 | 4.26E-27 | 0.6199 | A | G | 0.0175 | 0.0149 | 0.2405 | 757601 |
| rs1630736 | -0.1706 | 0.0309 | 3.52E-08 | 0.535 | T | C | -0.0246 | 0.0157 | 0.1166 | 757601 |
| rs167479 | -0.5642 | 0.0327 | 7.21E-67 | 0.5274 | T | G | 0.0159 | 0.0222 | 0.4738 | 757601 |
| rs17010957 | -0.534 | 0.043 | 1.78E-35 | 0.1463 | T | C | -0.0099 | 0.0208 | 0.6349 | 757601 |
| rs17035181 | 0.3074 | 0.0429 | 7.61E-13 | 0.1448 | T | G | -0.0097 | 0.0205 | 0.6374 | 757601 |
| rs17245822 | -0.1899 | 0.0312 | 1.15E-09 | 0.3733 | A | C | -0.013 | 0.0147 | 0.3767 | 757601 |
| rs17249754 | -0.8446 | 0.0403 | 1.25E-97 | 0.8317 | A | G | -0.0029 | 0.0189 | 0.8767 | 757601 |
| rs17257081 | 0.2274 | 0.0392 | 6.35E-09 | 0.1935 | A | G | -0.0226 | 0.0172 | 0.1883 | 757601 |
| rs1745417 | 0.2871 | 0.0301 | 1.59E-21 | 0.4799 | T | C | 0.0134 | 0.0143 | 0.3492 | 757601 |
| rs17562391 | 0.1967 | 0.0306 | 1.35E-10 | 0.5814 | T | C | -0.004 | 0.0146 | 0.7837 | 757601 |
| rs17608766 | -0.6903 | 0.0433 | 2.48E-57 | 0.1445 | T | C | 0.0216 | 0.0213 | 0.3112 | 757601 |
| rs17684859 | -0.2241 | 0.034 | 4.24E-11 | 0.2665 | T | C | 0.004 | 0.0162 | 0.8038 | 757601 |
| rs17760259 | -0.2654 | 0.0304 | 2.25E-18 | 0.4276 | T | C | -7.00E-04 | 0.0143 | 0.9599 | 757601 |
| rs17762 | 0.4117 | 0.0571 | 5.60E-13 | 0.9223 | A | G | -0.0395 | 0.0265 | 0.1356 | 757601 |
| rs17812022 | -0.3613 | 0.0525 | 5.65E-12 | 0.9042 | T | C | 0.0457 | 0.0256 | 0.07428 | 757601 |
| rs1814951 | -0.3231 | 0.0466 | 3.91E-12 | 0.1215 | A | G | 1.00E-04 | 0.0214 | 0.997 | 757601 |
| rs1871190 | 0.1954 | 0.0324 | 1.66E-09 | 0.6651 | T | G | -0.0033 | 0.0153 | 0.8282 | 757601 |
| rs1882961 | 0.2443 | 0.0326 | 6.69E-14 | 0.6913 | T | C | 0.0045 | 0.0156 | 0.7731 | 757601 |
| rs1889785 | 0.1782 | 0.0304 | 4.35E-09 | 0.5448 | A | G | -0.0083 | 0.0143 | 0.5624 | 757601 |
| rs1896326 | -0.2797 | 0.0371 | 4.41E-14 | 0.7709 | A | G | -0.0166 | 0.0179 | 0.3522 | 757601 |
| rs1906672 | 0.2966 | 0.0358 | 1.20E-16 | 0.7681 | A | G | -0.0097 | 0.0172 | 0.5745 | 757601 |
| rs1957563 | 0.3629 | 0.0342 | 2.32E-26 | 0.735 | T | C | -0.015 | 0.0162 | 0.3532 | 757601 |
| rs1984195 | 0.2409 | 0.0303 | 1.77E-15 | 0.5113 | A | G | 0.0194 | 0.0142 | 0.1728 | 757601 |
| rs2014408 | 0.5169 | 0.0373 | 1.26E-43 | 0.7913 | T | C | -0.0281 | 0.0177 | 0.1119 | 757601 |
| rs2046341 | -0.2542 | 0.0382 | 2.74E-11 | 0.8079 | A | G | -0.0089 | 0.0182 | 0.6251 | 757601 |
| rs2060664 | 0.216 | 0.0345 | 4.06E-10 | 0.2516 | T | C | 0.0154 | 0.0166 | 0.3532 | 757601 |
| rs2065498 | -0.2934 | 0.0403 | 3.36E-13 | 0.8294 | T | G | 0.0022 | 0.0185 | 0.9053 | 757601 |
| rs2111557 | 0.1764 | 0.0302 | 5.22E-09 | 0.5325 | T | C | -8.00E-04 | 0.0142 | 0.9538 | 757601 |
| rs2113077 | 0.2097 | 0.0305 | 6.09E-12 | 0.5697 | A | G | 0.0127 | 0.0148 | 0.3875 | 757601 |
| rs2126474 | -0.2601 | 0.0306 | 1.87E-17 | 0.5875 | T | G | -0.0027 | 0.0148 | 0.8559 | 757601 |
| rs2161967 | 0.2836 | 0.0307 | 2.87E-20 | 0.5721 | T | G | -0.0073 | 0.0146 | 0.6157 | 757601 |
| rs2177843 | 0.4394 | 0.0432 | 2.80E-24 | 0.8495 | T | C | -0.0409 | 0.0204 | 0.04538 | 757601 |
| rs2232460 | -0.2171 | 0.032 | 1.10E-11 | 0.6657 | A | G | 0.0308 | 0.015 | 0.04098 | 757601 |
| rs2236295 | -0.3028 | 0.0309 | 1.05E-22 | 0.6022 | T | G | 0.0301 | 0.0148 | 0.04159 | 757601 |
| rs2238787 | 0.2552 | 0.0332 | 1.45E-14 | 0.708 | A | G | -0.0302 | 0.0158 | 0.0565 | 757601 |
| rs2249105 | 0.2927 | 0.0313 | 7.63E-21 | 0.3679 | A | G | 0.0128 | 0.0147 | 0.3853 | 757601 |
| rs2289124 | -0.308 | 0.0415 | 1.14E-13 | 0.8327 | A | G | 0.002 | 0.0198 | 0.9205 | 757601 |
| rs2291434 | -0.2622 | 0.0303 | 5.10E-18 | 0.4665 | T | G | -0.0118 | 0.0146 | 0.4209 | 757601 |
| rs2306363 | -0.4358 | 0.0376 | 5.24E-31 | 0.7955 | T | G | 0.0109 | 0.0179 | 0.5412 | 757601 |
| rs2327429 | 0.2 | 0.0338 | 3.16E-09 | 0.2917 | T | C | -0.0163 | 0.016 | 0.3086 | 757601 |
| rs234623 | -0.1804 | 0.0302 | 2.43E-09 | 0.4959 | A | G | 0.0128 | 0.0145 | 0.3768 | 757601 |
| rs2353940 | -0.2075 | 0.0358 | 6.85E-09 | 0.2493 | T | C | 0.0354 | 0.0172 | 0.03927 | 757601 |
| rs2354862 | 0.2507 | 0.0317 | 2.42E-15 | 0.3593 | A | C | 0.0143 | 0.0148 | 0.3351 | 757601 |
| rs236916 | 0.3166 | 0.0446 | 1.31E-12 | 0.8652 | A | G | 0.0488 | 0.0208 | 0.01899 | 757601 |
| rs2384063 | 0.3266 | 0.0357 | 6.33E-20 | 0.2393 | T | C | 0.0235 | 0.0173 | 0.1737 | 757601 |
| rs2392929 | -0.7507 | 0.0379 | 1.96E-87 | 0.2027 | T | G | -0.0054 | 0.0182 | 0.7674 | 757601 |
| rs2423514 | 0.3011 | 0.0302 | 1.77E-23 | 0.4589 | A | G | -2.00E-04 | 0.0143 | 0.991 | 757601 |
| rs246973 | 0.2479 | 0.0335 | 1.45E-13 | 0.7118 | T | C | -0.0082 | 0.016 | 0.608 | 757601 |
| rs2470004 | -0.3454 | 0.0392 | 1.28E-18 | 0.1825 | T | C | -0.0223 | 0.0183 | 0.223 | 757601 |
| rs2493296 | 0.4183 | 0.0442 | 3.14E-21 | 0.8575 | T | C | -0.0254 | 0.0206 | 0.2172 | 757601 |
| rs2498323 | 0.3171 | 0.0517 | 8.52E-10 | 0.902 | A | G | -0.0493 | 0.0239 | 0.03945 | 757601 |
| rs2580350 | 0.1769 | 0.0307 | 8.39E-09 | 0.4391 | A | G | 0.0077 | 0.0147 | 0.6014 | 757601 |
| rs2589218 | -0.2258 | 0.0339 | 2.54E-11 | 0.2703 | T | C | -0.0101 | 0.0161 | 0.5293 | 757601 |
| rs2598 | 0.168 | 0.0303 | 2.87E-08 | 0.467 | A | G | -0.012 | 0.0144 | 0.4048 | 757601 |
| rs2610990 | -0.2903 | 0.0343 | 2.86E-17 | 0.7359 | A | G | -0.0231 | 0.0164 | 0.1587 | 757601 |
| rs2627313 | 0.3208 | 0.0303 | 3.55E-26 | 0.5546 | T | C | 0.0276 | 0.0143 | 0.05318 | 757601 |
| rs262986 | -0.2371 | 0.0305 | 7.67E-15 | 0.5296 | A | G | -0.0179 | 0.0146 | 0.22 | 757601 |
| rs263532 | 0.1798 | 0.0307 | 4.72E-09 | 0.4245 | T | C | 6.00E-04 | 0.0146 | 0.9657 | 757601 |
| rs2643826 | 0.4473 | 0.0306 | 1.74E-48 | 0.5495 | T | C | 0.0163 | 0.0146 | 0.2646 | 757601 |
| rs2652812 | -0.2516 | 0.0353 | 1.03E-12 | 0.2456 | T | C | 0.0215 | 0.0166 | 0.1935 | 757601 |
| rs2655445 | -0.2018 | 0.0312 | 9.58E-11 | 0.3933 | A | G | -0.0203 | 0.0149 | 0.1736 | 757601 |
| rs2689690 | -0.2702 | 0.0316 | 1.15E-17 | 0.6322 | T | C | 0.0114 | 0.0149 | 0.4456 | 757601 |
| rs2724377 | 0.1938 | 0.0301 | 1.29E-10 | 0.4697 | A | G | -0.0145 | 0.0144 | 0.3138 | 757601 |
| rs2745599 | 0.2164 | 0.0317 | 8.96E-12 | 0.448 | A | G | 0.0012 | 0.016 | 0.9386 | 757601 |
| rs2753960 | 0.4466 | 0.0309 | 2.66E-47 | 0.5801 | T | G | -0.0071 | 0.0145 | 0.6249 | 757601 |
| rs2776037 | -0.1851 | 0.0309 | 2.15E-09 | 0.5849 | T | C | 0.0096 | 0.0146 | 0.5105 | 757601 |
| rs2815063 | 0.2755 | 0.0458 | 1.76E-09 | 0.8685 | A | C | 0.0565 | 0.022 | 0.01026 | 757601 |
| rs2833834 | 0.2177 | 0.0338 | 1.22E-10 | 0.7235 | A | C | 0.0308 | 0.0159 | 0.05271 | 757601 |
| rs28374392 | 0.1924 | 0.0338 | 1.21E-08 | 0.3769 | T | C | -0.0038 | 0.0168 | 0.8205 | 757601 |
| rs28429256 | 0.215 | 0.0325 | 3.89E-11 | 0.6658 | A | G | 0.0319 | 0.0159 | 0.04475 | 757601 |
| rs28572357 | -0.2733 | 0.0308 | 6.34E-19 | 0.3977 | A | C | 0.0263 | 0.0148 | 0.07574 | 757601 |
| rs28578714 | 0.2066 | 0.0327 | 2.53E-10 | 0.3938 | T | C | 0.0268 | 0.0177 | 0.1293 | 757601 |
| rs28688791 | -0.3222 | 0.038 | 2.34E-17 | 0.1982 | T | C | 0.0074 | 0.0177 | 0.6761 | 757601 |
| rs28866311 | -0.2762 | 0.0302 | 5.45E-20 | 0.4737 | T | G | 0.0198 | 0.0147 | 0.1783 | 757601 |
| rs2900568 | -0.1889 | 0.03 | 2.96E-10 | 0.4816 | T | C | -0.0021 | 0.0142 | 0.8837 | 757601 |
| rs2904315 | -0.2081 | 0.0325 | 1.58E-10 | 0.6869 | A | G | 0.0349 | 0.0154 | 0.02344 | 757601 |
| rs2913920 | 0.2418 | 0.0359 | 1.62E-11 | 0.235 | T | C | -0.0211 | 0.0167 | 0.2074 | 757601 |
| rs3098186 | -0.2422 | 0.0303 | 1.41E-15 | 0.4844 | T | C | -0.045 | 0.0146 | 0.001971 | 757601 |
| rs33836 | 0.1766 | 0.0304 | 6.56E-09 | 0.5378 | T | C | 0.0126 | 0.0145 | 0.3844 | 757601 |
| rs34025993 | 0.223 | 0.0308 | 4.71E-13 | 0.586 | A | G | 0.0032 | 0.015 | 0.8293 | 757601 |
| rs34072724 | -0.2422 | 0.0303 | 1.37E-15 | 0.5111 | A | G | -0.0058 | 0.0146 | 0.6915 | 757601 |
| rs34079867 | 0.1992 | 0.0354 | 1.78E-08 | 0.734 | T | C | -0.0171 | 0.0174 | 0.3235 | 757601 |
| rs34130368 | -0.3016 | 0.0497 | 1.28E-09 | 0.883 | T | G | 4.00E-04 | 0.0244 | 0.9872 | 757601 |
| rs34487963 | -0.8819 | 0.1244 | 1.35E-12 | 0.9815 | A | C | 0.0263 | 0.06 | 0.6607 | 757601 |
| rs34496659 | 0.4545 | 0.0616 | 1.54E-13 | 0.9298 | A | G | -0.0055 | 0.0301 | 0.8541 | 757601 |
| rs34535756 | 0.478 | 0.0786 | 1.18E-09 | 0.9606 | T | C | -0.0168 | 0.0361 | 0.6411 | 757601 |
| rs34941092 | -0.3225 | 0.0425 | 3.23E-14 | 0.8502 | A | G | 0.0349 | 0.0199 | 0.08011 | 757601 |
| rs35413927 | -0.3002 | 0.0328 | 5.25E-20 | 0.3054 | A | G | 0.0195 | 0.0156 | 0.2114 | 757601 |
| rs35444 | 0.4368 | 0.031 | 3.47E-45 | 0.3862 | A | G | 0.0077 | 0.0149 | 0.6036 | 757601 |
| rs35680304 | 0.2694 | 0.031 | 3.76E-18 | 0.4071 | T | C | 0.0059 | 0.0148 | 0.6903 | 757601 |
| rs35783704 | -0.4619 | 0.0507 | 8.81E-20 | 0.8958 | A | G | -0.0238 | 0.0249 | 0.3391 | 757601 |
| rs360153 | -0.3445 | 0.0306 | 1.73E-29 | 0.5834 | T | C | 0.015 | 0.0145 | 0.2994 | 757601 |
| rs365990 | 0.225 | 0.0312 | 5.95E-13 | 0.3658 | A | G | 0.0058 | 0.015 | 0.6958 | 757601 |
| rs3735533 | -0.91 | 0.0577 | 5.29E-56 | 0.9257 | T | C | -0.0173 | 0.0277 | 0.5309 | 757601 |
| rs3754944 | 0.1768 | 0.0308 | 9.30E-09 | 0.4125 | A | C | -0.0028 | 0.0144 | 0.846 | 757601 |
| rs3764400 | 0.3748 | 0.0445 | 3.69E-17 | 0.1365 | T | C | -0.0511 | 0.0217 | 0.01864 | 757601 |
| rs3772219 | 0.2733 | 0.0324 | 3.10E-17 | 0.3176 | A | C | -0.0441 | 0.0155 | 0.004517 | 757601 |
| rs3807925 | -0.1859 | 0.0319 | 5.39E-09 | 0.3504 | A | G | -4.00E-04 | 0.0152 | 0.9813 | 757601 |
| rs3819532 | -0.1875 | 0.0306 | 9.44E-10 | 0.6087 | T | C | 0.0014 | 0.0146 | 0.9234 | 757601 |
| rs3860770 | -0.2663 | 0.0333 | 1.20E-15 | 0.7084 | A | G | -0.0034 | 0.0161 | 0.83 | 757601 |
| rs3918226 | 0.664 | 0.0575 | 8.46E-31 | 0.9189 | T | C | -0.0337 | 0.0284 | 0.2361 | 757601 |
| rs3950627 | 0.1851 | 0.0308 | 1.82E-09 | 0.469 | A | C | 0.004 | 0.0144 | 0.7834 | 757601 |
| rs3980686 | -0.4998 | 0.0487 | 1.03E-24 | 0.8925 | T | G | 0.0095 | 0.0221 | 0.6662 | 757601 |
| rs404100 | 0.1935 | 0.0303 | 1.68E-10 | 0.5487 | T | C | 0.0045 | 0.0143 | 0.7512 | 757601 |
| rs4143175 | 0.2187 | 0.0352 | 5.10E-10 | 0.7591 | T | C | 0.0114 | 0.017 | 0.5022 | 757601 |
| rs42377 | -0.3153 | 0.0331 | 1.69E-21 | 0.6955 | A | G | -0.0534 | 0.0154 | 0.0005432 | 757601 |
| rs4245599 | -0.1794 | 0.0305 | 4.04E-09 | 0.5416 | A | G | 0.0285 | 0.0143 | 0.04621 | 757601 |
| rs4286632 | 0.211 | 0.0343 | 7.64E-10 | 0.2694 | A | G | 0 | 0.0167 | 0.998 | 757601 |
| rs4408839 | -0.2301 | 0.0345 | 2.43E-11 | 0.2567 | A | G | 0.0082 | 0.0166 | 0.6222 | 757601 |
| rs4427587 | 0.2062 | 0.0313 | 4.28E-11 | 0.4381 | T | C | -0.013 | 0.015 | 0.3865 | 757601 |
| rs4440615 | -0.2201 | 0.0312 | 1.87E-12 | 0.3679 | A | G | 0.0204 | 0.0149 | 0.1724 | 757601 |
| rs4511593 | -0.2881 | 0.0318 | 1.28E-19 | 0.3472 | T | C | -0.0089 | 0.0151 | 0.5568 | 757601 |
| rs4553000 | -0.2035 | 0.03 | 1.09E-11 | 0.4859 | T | C | -0.0182 | 0.0142 | 0.2006 | 757601 |
| rs4577304 | -0.1767 | 0.0302 | 4.99E-09 | 0.4767 | T | C | -0.021 | 0.0144 | 0.144 | 757601 |
| rs4595370 | -0.2092 | 0.0328 | 1.73E-10 | 0.6988 | A | G | -0.0022 | 0.0154 | 0.8858 | 757601 |
| rs4598218 | 0.1911 | 0.0313 | 1.00E-09 | 0.3842 | T | C | -0.0106 | 0.0148 | 0.4721 | 757601 |
| rs4606697 | -0.3196 | 0.0523 | 9.71E-10 | 0.8959 | A | G | 0.0016 | 0.0281 | 0.956 | 757601 |
| rs4651224 | 0.1986 | 0.0306 | 9.00E-11 | 0.5526 | T | C | 0.0149 | 0.0144 | 0.3007 | 757601 |
| rs4667454 | 0.2636 | 0.0322 | 2.63E-16 | 0.3295 | A | G | -0.0026 | 0.0155 | 0.8663 | 757601 |
| rs4775769 | -0.4162 | 0.0517 | 7.76E-16 | 0.9055 | T | G | -0.0105 | 0.024 | 0.6624 | 757601 |
| rs4784541 | -0.2015 | 0.0307 | 4.93E-11 | 0.5252 | T | C | -0.0198 | 0.0152 | 0.1937 | 757601 |
| rs483071 | 0.2709 | 0.0313 | 5.09E-18 | 0.3752 | T | C | -0.0046 | 0.0149 | 0.7576 | 757601 |
| rs4873492 | 0.3431 | 0.0403 | 1.61E-17 | 0.8276 | T | C | 0.0199 | 0.0185 | 0.2812 | 757601 |
| rs488834 | -0.3799 | 0.0365 | 2.35E-25 | 0.2355 | T | C | -0.0111 | 0.0173 | 0.5215 | 757601 |
| rs4888408 | 0.3653 | 0.0307 | 1.42E-32 | 0.4145 | A | G | -0.0132 | 0.0146 | 0.3678 | 757601 |
| rs4894132 | 0.2469 | 0.0342 | 5.51E-13 | 0.2717 | T | C | -8.00E-04 | 0.0167 | 0.9598 | 757601 |
| rs4908348 | 0.2366 | 0.033 | 8.07E-13 | 0.3056 | T | G | 0.0136 | 0.016 | 0.394 | 757601 |
| rs4925159 | 0.2174 | 0.0305 | 9.66E-13 | 0.5754 | A | G | 0.0166 | 0.0144 | 0.2491 | 757601 |
| rs4932373 | -0.635 | 0.0328 | 2.49E-83 | 0.3258 | A | C | -0.0052 | 0.0163 | 0.7484 | 757601 |
| rs4948643 | 0.2258 | 0.0338 | 2.40E-11 | 0.7181 | T | C | -0.0037 | 0.0158 | 0.8167 | 757601 |
| rs4952609 | 0.2124 | 0.0347 | 9.60E-10 | 0.2561 | A | G | -0.021 | 0.0163 | 0.1977 | 757601 |
| rs4955575 | 0.2158 | 0.0348 | 5.63E-10 | 0.2539 | A | C | -0.0262 | 0.0165 | 0.1116 | 757601 |
| rs4957026 | 0.1982 | 0.0323 | 8.12E-10 | 0.6601 | A | G | 0.0094 | 0.0152 | 0.5377 | 757601 |
| rs4961293 | 0.2268 | 0.0303 | 7.35E-14 | 0.5487 | T | C | 0.004 | 0.0144 | 0.7824 | 757601 |
| rs5020545 | -0.2179 | 0.0305 | 9.71E-13 | 0.5563 | T | C | -0.0052 | 0.0146 | 0.7223 | 757601 |
| rs509833 | 0.329 | 0.044 | 7.08E-14 | 0.8614 | A | G | -0.0163 | 0.0209 | 0.4367 | 757601 |
| rs55924432 | 0.2651 | 0.0317 | 5.70E-17 | 0.599 | T | C | -0.0179 | 0.0156 | 0.2522 | 757601 |
| rs55944332 | -0.2613 | 0.0355 | 1.79E-13 | 0.2368 | A | G | 0.0081 | 0.017 | 0.6349 | 757601 |
| rs56288724 | -0.2178 | 0.031 | 2.01E-12 | 0.4169 | A | G | -0.0161 | 0.0149 | 0.278 | 757601 |
| rs56407827 | 0.3603 | 0.034 | 2.78E-26 | 0.7313 | T | C | -8.00E-04 | 0.0161 | 0.9609 | 757601 |
| rs569550 | -0.5765 | 0.0318 | 1.33E-73 | 0.3963 | T | G | 0.0191 | 0.0152 | 0.209 | 757601 |
| rs571689 | 0.228 | 0.0304 | 6.77E-14 | 0.4804 | T | C | 0.0208 | 0.0146 | 0.1536 | 757601 |
| rs573455 | 0.1994 | 0.0303 | 4.77E-11 | 0.539 | A | G | 0.0108 | 0.0145 | 0.4578 | 757601 |
| rs5742643 | -0.2233 | 0.0349 | 1.53E-10 | 0.7513 | T | C | -0.023 | 0.0166 | 0.1654 | 757601 |
| rs57946343 | 0.716 | 0.0426 | 2.10E-63 | 0.1473 | T | C | 0.0043 | 0.0199 | 0.8308 | 757601 |
| rs59980837 | 1.0997 | 0.1163 | 3.32E-21 | 0.9822 | T | G | 0.0703 | 0.0567 | 0.2152 | 757601 |
| rs60191654 | -0.2382 | 0.0385 | 5.88E-10 | 0.1882 | A | G | -0.009 | 0.0193 | 0.642 | 757601 |
| rs6029756 | -0.2712 | 0.033 | 1.88E-16 | 0.6775 | A | G | 0.0101 | 0.0159 | 0.5257 | 757601 |
| rs6031431 | -0.2617 | 0.0304 | 7.05E-18 | 0.4624 | A | G | -0.0124 | 0.0146 | 0.3925 | 757601 |
| rs604723 | -0.655 | 0.0339 | 2.55E-83 | 0.7244 | T | C | -0.001 | 0.0158 | 0.9477 | 757601 |
| rs6054139 | 0.2094 | 0.0306 | 8.23E-12 | 0.394 | A | G | 0.001 | 0.0147 | 0.9453 | 757601 |
| rs6058088 | 0.2832 | 0.0417 | 1.14E-11 | 0.1561 | T | G | 0.0013 | 0.0195 | 0.9487 | 757601 |
| rs6062324 | -0.3294 | 0.0363 | 1.18E-19 | 0.7636 | A | G | 0.0322 | 0.0173 | 0.06191 | 757601 |
| rs6078093 | -0.1849 | 0.0304 | 1.20E-09 | 0.572 | A | G | -0.0059 | 0.0148 | 0.6899 | 757601 |
| rs6090907 | -0.3854 | 0.0425 | 1.29E-19 | 0.853 | A | G | 0.0385 | 0.0199 | 0.05356 | 757601 |
| rs60991988 | 0.3789 | 0.0498 | 2.82E-14 | 0.1069 | T | G | 0.0083 | 0.0228 | 0.7157 | 757601 |
| rs6108787 | -0.4274 | 0.03 | 5.38E-46 | 0.4704 | T | G | 0.0014 | 0.0143 | 0.9233 | 757601 |
| rs61772592 | -0.3181 | 0.0455 | 2.86E-12 | 0.1255 | A | G | -0.016 | 0.0214 | 0.4557 | 757601 |
| rs61917655 | 0.3427 | 0.0514 | 2.68E-11 | 0.8986 | T | C | -0.0013 | 0.0245 | 0.9578 | 757601 |
| rs62047964 | 0.5115 | 0.0686 | 9.29E-14 | 0.9378 | T | C | -0.1042 | 0.0353 | 0.003208 | 757601 |
| rs62076622 | 0.2363 | 0.0377 | 3.79E-10 | 0.1987 | A | G | -0.0368 | 0.0181 | 0.0416 | 757601 |
| rs62112908 | -0.2388 | 0.0419 | 1.25E-08 | 0.1536 | A | G | 0.0034 | 0.02 | 0.8632 | 757601 |
| rs62170470 | 0.1972 | 0.0321 | 7.69E-10 | 0.3983 | T | C | -0.0281 | 0.0154 | 0.06741 | 757601 |
| rs62187653 | 0.3286 | 0.0511 | 1.23E-10 | 0.0971 | T | C | 0.0155 | 0.0235 | 0.5093 | 757601 |
| rs62309747 | -0.2244 | 0.0304 | 1.59E-13 | 0.5266 | A | G | 0.0332 | 0.0145 | 0.02192 | 757601 |
| rs6271 | -0.5547 | 0.0611 | 1.18E-19 | 0.9265 | T | C | -0.0258 | 0.037 | 0.4865 | 757601 |
| rs629864 | -0.1868 | 0.0319 | 4.69E-09 | 0.3503 | T | C | 0.006 | 0.0151 | 0.6931 | 757601 |
| rs6438857 | 0.2736 | 0.0305 | 3.13E-19 | 0.4226 | T | C | 0.0237 | 0.0144 | 0.09943 | 757601 |
| rs6445583 | 0.2774 | 0.0349 | 1.90E-15 | 0.2535 | A | G | -0.0173 | 0.0167 | 0.2998 | 757601 |
| rs6452769 | -0.3143 | 0.0377 | 7.82E-17 | 0.7947 | A | G | -0.0128 | 0.0177 | 0.4693 | 757601 |
| rs6490019 | -0.2897 | 0.0309 | 6.61E-21 | 0.6204 | A | G | 0.0195 | 0.0147 | 0.1841 | 757601 |
| rs6504213 | -0.2982 | 0.0312 | 1.25E-21 | 0.5818 | T | C | -0.0046 | 0.0153 | 0.7647 | 757601 |
| rs6539467 | 0.265 | 0.0404 | 5.57E-11 | 0.8339 | A | G | 0.0222 | 0.0191 | 0.2454 | 757601 |
| rs6562778 | 0.178 | 0.0304 | 4.96E-09 | 0.5411 | A | G | -0.0085 | 0.0148 | 0.5636 | 757601 |
| rs658780 | -0.2028 | 0.0347 | 5.29E-09 | 0.2553 | T | G | 0.0193 | 0.0167 | 0.2496 | 757601 |
| rs665445 | -0.1909 | 0.0334 | 1.15E-08 | 0.7206 | A | C | 0.011 | 0.0158 | 0.488 | 757601 |
| rs6731373 | 0.1913 | 0.0326 | 4.18E-09 | 0.6508 | A | G | 6.00E-04 | 0.0162 | 0.9704 | 757601 |
| rs6737318 | 0.2348 | 0.0364 | 1.13E-10 | 0.2218 | A | G | 0.0073 | 0.0174 | 0.673 | 757601 |
| rs6771917 | -0.3793 | 0.0355 | 1.39E-26 | 0.7523 | T | C | -0.0105 | 0.0165 | 0.5254 | 757601 |
| rs6788984 | 0.2999 | 0.0432 | 3.81E-12 | 0.1437 | A | G | -0.0116 | 0.0195 | 0.5509 | 757601 |
| rs68085857 | 0.274 | 0.0357 | 1.68E-14 | 0.766 | T | C | -0.0213 | 0.0167 | 0.2042 | 757601 |
| rs68096471 | -0.2098 | 0.0343 | 9.26E-10 | 0.7341 | A | G | -0.0129 | 0.0167 | 0.4396 | 757601 |
| rs68115553 | -0.6445 | 0.1143 | 1.74E-08 | 0.0199 | A | G | -0.0317 | 0.0583 | 0.5869 | 757601 |
| rs6823199 | 0.2094 | 0.0348 | 1.72E-09 | 0.2562 | T | C | -0.02 | 0.0163 | 0.2191 | 757601 |
| rs6870654 | 0.2136 | 0.0347 | 7.58E-10 | 0.2546 | T | C | 0.0088 | 0.0162 | 0.5862 | 757601 |
| rs6892983 | 0.3427 | 0.0307 | 7.11E-29 | 0.5978 | A | C | 0.0053 | 0.0145 | 0.7132 | 757601 |
| rs6921291 | 0.3575 | 0.0385 | 1.58E-20 | 0.8093 | T | C | -0.0319 | 0.0179 | 0.07549 | 757601 |
| rs6959688 | -0.2344 | 0.031 | 4.22E-14 | 0.4019 | A | G | 0.033 | 0.0148 | 0.02597 | 757601 |
| rs698748 | 0.1871 | 0.0325 | 8.90E-09 | 0.579 | A | G | -0.0012 | 0.0166 | 0.9442 | 757601 |
| rs699 | -0.3748 | 0.0308 | 5.59E-34 | 0.4072 | A | G | 0.0314 | 0.0144 | 0.02953 | 757601 |
| rs7012866 | -0.2325 | 0.0301 | 1.21E-14 | 0.5009 | T | G | 0.0076 | 0.0142 | 0.5953 | 757601 |
| rs702395 | 0.2318 | 0.0305 | 3.24E-14 | 0.5631 | T | C | 0.0408 | 0.0146 | 0.005262 | 757601 |
| rs7093894 | 0.236 | 0.0427 | 3.16E-08 | 0.8488 | A | C | 0.0487 | 0.0197 | 0.0133 | 757601 |
| rs7107356 | -0.4598 | 0.0301 | 1.63E-52 | 0.5041 | A | G | -0.0668 | 0.0143 | 2.79E-06 | 757601 |
| rs7125196 | 0.4422 | 0.0472 | 7.31E-21 | 0.1183 | T | C | -0.0017 | 0.0231 | 0.9407 | 757601 |
| rs7134440 | 0.4788 | 0.0562 | 1.58E-17 | 0.9178 | T | C | -0.0383 | 0.027 | 0.1554 | 757601 |
| rs7134677 | -0.3851 | 0.0332 | 4.46E-31 | 0.7022 | T | C | -0.0077 | 0.0162 | 0.6344 | 757601 |
| rs7154723 | 0.253 | 0.0309 | 2.72E-16 | 0.615 | A | G | -0.0068 | 0.0146 | 0.64 | 757601 |
| rs7186298 | -0.2315 | 0.0302 | 1.88E-14 | 0.5705 | T | C | 0.0283 | 0.0145 | 0.05146 | 757601 |
| rs7213273 | -0.4 | 0.0315 | 6.24E-37 | 0.345 | A | G | -0.004 | 0.0149 | 0.7883 | 757601 |
| rs7218708 | -0.1781 | 0.0303 | 4.38E-09 | 0.5169 | A | G | -0.0175 | 0.0143 | 0.2212 | 757601 |
| rs7245140 | -0.3367 | 0.0391 | 7.67E-18 | 0.1802 | T | C | 0.0071 | 0.0186 | 0.7041 | 757601 |
| rs7255933 | 0.2306 | 0.0345 | 2.44E-11 | 0.7426 | A | G | -0.0569 | 0.0166 | 0.0006272 | 757601 |
| rs72683923 | 0.9587 | 0.1101 | 3.08E-18 | 0.0212 | T | C | 0.1035 | 0.0504 | 0.03993 | 757601 |
| rs72778133 | -0.2417 | 0.0443 | 4.98E-08 | 0.1422 | T | C | -0.0038 | 0.0214 | 0.8585 | 757601 |
| rs7278003 | -0.1876 | 0.0304 | 6.63E-10 | 0.5622 | T | C | -0.0078 | 0.0144 | 0.5904 | 757601 |
| rs72842207 | -0.203 | 0.0367 | 3.14E-08 | 0.7856 | T | C | 0.0159 | 0.0175 | 0.3621 | 757601 |
| rs72847885 | 0.2413 | 0.0318 | 3.08E-14 | 0.337 | A | G | -0.003 | 0.015 | 0.8394 | 757601 |
| rs73046792 | -0.3554 | 0.0426 | 7.23E-17 | 0.8412 | A | G | -0.0222 | 0.0213 | 0.2965 | 757601 |
| rs73049928 | -0.2382 | 0.0392 | 1.20E-09 | 0.1939 | A | G | -0.0533 | 0.019 | 0.005065 | 757601 |
| rs7306710 | -0.2429 | 0.0303 | 1.03E-15 | 0.519 | T | C | -0.013 | 0.0146 | 0.3722 | 757601 |
| rs73075659 | 0.3962 | 0.0321 | 5.52E-35 | 0.3346 | A | G | 0.0165 | 0.0154 | 0.2841 | 757601 |
| rs7331680 | 0.4101 | 0.0423 | 3.35E-22 | 0.8509 | T | G | -0.0097 | 0.0201 | 0.6299 | 757601 |
| rs73727605 | 0.3616 | 0.0623 | 6.60E-09 | 0.9337 | A | G | -0.0136 | 0.0311 | 0.6627 | 757601 |
| rs73855810 | 0.2732 | 0.0434 | 3.04E-10 | 0.8594 | A | G | -0.0297 | 0.0203 | 0.1436 | 757601 |
| rs7395791 | -0.2162 | 0.0308 | 2.19E-12 | 0.5581 | A | G | -0.0052 | 0.0146 | 0.722 | 757601 |
| rs74048190 | -0.4404 | 0.0757 | 6.07E-09 | 0.0478 | T | C | -0.022 | 0.0371 | 0.5528 | 757601 |
| rs743395 | 0.2597 | 0.0317 | 2.55E-16 | 0.6166 | T | C | -0.0226 | 0.0151 | 0.1357 | 757601 |
| rs7439567 | 0.2537 | 0.0309 | 2.31E-16 | 0.5894 | T | C | -0.024 | 0.0145 | 0.09778 | 757601 |
| rs7491248 | 0.2163 | 0.0362 | 2.38E-09 | 0.7761 | A | G | -0.0103 | 0.017 | 0.5429 | 757601 |
| rs75016974 | -0.2513 | 0.0439 | 1.05E-08 | 0.8577 | T | C | -0.012 | 0.0215 | 0.5751 | 757601 |
| rs7514579 | 0.2243 | 0.0361 | 5.45E-10 | 0.2288 | A | C | -0.0306 | 0.0174 | 0.07935 | 757601 |
| rs75461554 | -0.3016 | 0.0377 | 1.18E-15 | 0.7993 | T | C | 0.0092 | 0.0176 | 0.6002 | 757601 |
| rs75672964 | 0.5885 | 0.0839 | 2.35E-12 | 0.9582 | T | C | 0.0593 | 0.0464 | 0.2011 | 757601 |
| rs75961402 | 0.2659 | 0.0418 | 1.95E-10 | 0.8466 | A | G | 0.0022 | 0.0198 | 0.9101 | 757601 |
| rs7615099 | 0.1891 | 0.0321 | 3.90E-09 | 0.3325 | A | G | 0.0011 | 0.0153 | 0.9411 | 757601 |
| rs76452347 | -0.2974 | 0.0397 | 7.13E-14 | 0.795 | T | C | -0.0019 | 0.0199 | 0.9252 | 757601 |
| rs76719272 | -0.2738 | 0.0461 | 2.97E-09 | 0.8688 | T | C | -0.0569 | 0.023 | 0.0133 | 757601 |
| rs7683728 | -0.3654 | 0.0304 | 2.43E-33 | 0.4688 | T | C | 0.0337 | 0.0142 | 0.01791 | 757601 |
| rs77032376 | -0.2727 | 0.043 | 2.35E-10 | 0.8515 | T | C | -0.0057 | 0.021 | 0.7855 | 757601 |
| rs7703560 | -0.2246 | 0.0333 | 1.51E-11 | 0.2998 | A | G | -0.0035 | 0.0157 | 0.8249 | 757601 |
| rs7725413 | -0.1985 | 0.0359 | 3.07E-08 | 0.2301 | T | C | 0.024 | 0.0171 | 0.16 | 757601 |
| rs77375686 | -0.3467 | 0.0485 | 8.38E-13 | 0.1117 | A | G | 0.0146 | 0.0228 | 0.5212 | 757601 |
| rs7744902 | -0.4088 | 0.0593 | 5.64E-12 | 0.9234 | A | G | -0.0241 | 0.0281 | 0.3906 | 757601 |
| rs7763558 | 0.3363 | 0.0321 | 1.17E-25 | 0.6759 | A | G | 0.0038 | 0.0151 | 0.8033 | 757601 |
| rs7765526 | 0.201 | 0.0307 | 5.88E-11 | 0.5367 | A | G | -0.0152 | 0.0148 | 0.3021 | 757601 |
| rs778124 | 0.2965 | 0.0311 | 1.45E-21 | 0.6264 | A | G | 0.0042 | 0.0147 | 0.7765 | 757601 |
| rs77924615 | -0.4081 | 0.039 | 1.12E-25 | 0.8014 | A | G | -0.0131 | 0.0189 | 0.4878 | 757601 |
| rs7821832 | 0.4222 | 0.0348 | 6.67E-34 | 0.2553 | T | G | 0.0135 | 0.0163 | 0.4076 | 757601 |
| rs7830607 | -0.206 | 0.0327 | 3.09E-10 | 0.6954 | A | G | 0.0276 | 0.0156 | 0.07706 | 757601 |
| rs78474310 | -0.4699 | 0.0734 | 1.51E-10 | 0.0448 | A | G | -0.0042 | 0.0371 | 0.909 | 757601 |
| rs7854147 | 0.3056 | 0.0461 | 3.29E-11 | 0.123 | A | G | 0.0019 | 0.0227 | 0.932 | 757601 |
| rs78648104 | -0.4287 | 0.0541 | 2.37E-15 | 0.0925 | T | C | 0.035 | 0.0265 | 0.1866 | 757601 |
| rs786923 | -0.3082 | 0.031 | 2.83E-23 | 0.3761 | T | C | 0.0031 | 0.0146 | 0.8332 | 757601 |
| rs79069610 | -0.4005 | 0.0727 | 3.68E-08 | 0.05 | T | C | 0.0132 | 0.0339 | 0.6972 | 757601 |
| rs7912283 | -0.2144 | 0.0322 | 2.94E-11 | 0.3532 | A | G | -0.0108 | 0.0157 | 0.4911 | 757601 |
| rs7926110 | 0.2603 | 0.0321 | 5.71E-16 | 0.3267 | T | G | -0.0037 | 0.0152 | 0.8072 | 757601 |
| rs7926335 | 0.3135 | 0.0339 | 2.52E-20 | 0.7309 | T | C | 0.0211 | 0.0161 | 0.1892 | 757601 |
| rs7927515 | 0.2271 | 0.0319 | 1.05E-12 | 0.6541 | A | C | -0.0074 | 0.0149 | 0.6186 | 757601 |
| rs79384779 | 0.3179 | 0.0428 | 1.08E-13 | 0.8488 | T | C | -0.0179 | 0.0206 | 0.3844 | 757601 |
| rs7944927 | 0.2235 | 0.0392 | 1.23E-08 | 0.2181 | T | C | -0.0131 | 0.02 | 0.5106 | 757601 |
| rs79539362 | 0.4003 | 0.0504 | 2.09E-15 | 0.1008 | T | C | 0.0104 | 0.0244 | 0.6691 | 757601 |
| rs7963801 | -0.2362 | 0.0311 | 2.87E-14 | 0.5779 | T | C | 0.0142 | 0.015 | 0.3441 | 757601 |
| rs79782817 | 0.5324 | 0.0499 | 1.43E-26 | 0.8973 | T | G | -0.0208 | 0.0232 | 0.3698 | 757601 |
| rs8044992 | 0.2138 | 0.0331 | 1.07E-10 | 0.2877 | T | C | -0.0016 | 0.0157 | 0.9194 | 757601 |
| rs8125763 | 0.1761 | 0.0301 | 4.84E-09 | 0.5283 | A | C | -0.0355 | 0.0143 | 0.01268 | 757601 |
| rs8142376 | 0.1676 | 0.03 | 2.20E-08 | 0.509 | T | C | -0.0059 | 0.0142 | 0.6804 | 757601 |
| rs8180684 | 0.2134 | 0.0335 | 1.80E-10 | 0.7104 | T | C | -0.0062 | 0.0159 | 0.6969 | 757601 |
| rs843093 | -0.2085 | 0.0338 | 6.95E-10 | 0.2912 | A | G | -0.0241 | 0.0166 | 0.1447 | 757601 |
| rs848445 | -0.2025 | 0.0339 | 2.28E-09 | 0.7149 | T | C | 0.0193 | 0.0162 | 0.2354 | 757601 |
| rs869396 | -0.2115 | 0.0305 | 4.12E-12 | 0.5341 | A | C | 0.0011 | 0.0145 | 0.9381 | 757601 |
| rs871004 | 0.2336 | 0.0317 | 1.65E-13 | 0.6519 | A | G | 0.0081 | 0.0149 | 0.5848 | 757601 |
| rs8904 | 0.3061 | 0.0314 | 1.71E-22 | 0.6322 | A | G | 0.0209 | 0.0149 | 0.1608 | 757601 |
| rs908951 | -0.2261 | 0.0315 | 7.14E-13 | 0.5622 | T | C | 0.0161 | 0.0154 | 0.2956 | 757601 |
| rs927315 | 0.1689 | 0.0303 | 2.44E-08 | 0.5287 | T | C | -0.011 | 0.0152 | 0.4686 | 757601 |
| rs9302885 | 0.2242 | 0.0302 | 1.03E-13 | 0.5548 | A | G | 0.0102 | 0.0144 | 0.4786 | 757601 |
| rs9303175 | -0.2048 | 0.0327 | 3.65E-10 | 0.6537 | T | G | -0.0044 | 0.0159 | 0.783 | 757601 |
| rs9349379 | 0.2664 | 0.0312 | 1.31E-17 | 0.407 | A | G | -0.0067 | 0.0148 | 0.6496 | 757601 |
| rs9361836 | 0.2196 | 0.0324 | 1.25E-11 | 0.6828 | T | C | 0.0045 | 0.0152 | 0.7652 | 757601 |
| rs9368222 | 0.2281 | 0.0339 | 1.84E-11 | 0.7312 | A | C | -0.0105 | 0.016 | 0.5103 | 757601 |
| rs9401913 | 0.5202 | 0.0305 | 3.66E-65 | 0.5613 | A | G | 0.0072 | 0.0146 | 0.6225 | 757601 |
| rs9486916 | 0.2657 | 0.0385 | 5.42E-12 | 0.8021 | T | C | -0.0254 | 0.0181 | 0.1595 | 757601 |
| rs9507885 | -0.3208 | 0.0542 | 3.23E-09 | 0.9047 | T | C | 2.00E-04 | 0.0266 | 0.9935 | 757601 |
| rs9508495 | -0.3557 | 0.0353 | 6.34E-24 | 0.2435 | T | C | 0.0032 | 0.0168 | 0.849 | 757601 |
| rs9526707 | -0.2039 | 0.0323 | 2.77E-10 | 0.6784 | A | G | -0.0128 | 0.0155 | 0.4087 | 757601 |
| rs9549627 | 0.2846 | 0.05 | 1.25E-08 | 0.8825 | A | G | -0.0027 | 0.026 | 0.9169 | 757601 |
| rs9651825 | -0.2042 | 0.034 | 1.93E-09 | 0.2705 | A | G | -0.0126 | 0.0164 | 0.4408 | 757601 |
| rs977184 | -0.184 | 0.0314 | 4.86E-09 | 0.3748 | T | C | -0.0059 | 0.0149 | 0.6922 | 757601 |
| rs9869437 | -0.2001 | 0.0318 | 3.22E-10 | 0.6477 | A | C | -0.0301 | 0.0153 | 0.04961 | 757601 |
| rs9876694 | 0.4713 | 0.0651 | 4.64E-13 | 0.9416 | T | C | 0.0021 | 0.0314 | 0.9467 | 757601 |
| rs9880098 | 0.3081 | 0.0308 | 1.59E-23 | 0.6054 | A | G | 0.0188 | 0.0145 | 0.1947 | 757601 |
| rs9886665 | 0.2048 | 0.0343 | 2.47E-09 | 0.7329 | T | C | 0.0274 | 0.0171 | 0.1088 | 757601 |
| rs9897429 | 0.2645 | 0.0319 | 1.19E-16 | 0.48 | A | G | 0.0313 | 0.0166 | 0.05913 | 757601 |
| rs9918879 | -0.2984 | 0.0499 | 2.28E-09 | 0.897 | T | G | 0.0381 | 0.0242 | 0.1157 | 757601 |
