# Supplementary material for: Genetically determined blood pressure, antihypertensive medications, and risk of Alzheimer’s disease: a Mendelian randomization study
Source: Alzheimers Res Ther. 2021 Feb 9;13:41. doi: 10.1186/s13195-021-00782-y (PMC7874453; doi:10.1186/s13195-021-00782-y)
Supplement: Supplementary file 4 — Additional file 4. Genome-wide significant and independent SNPs that were used as instruments for DBP. [file 13195_2021_782_MOESM4_ESM.docx]

**Additional file 4 Genome-wide significant and independent SNPs that were used as instruments for DBP**

| SNP | Beta.exposure | SE.exposure | Pval.exposure | EAF | Effect_allele | Other_allele | Beta.outcome | SE.outcome | Pval.outcome | Samplesize |
| --- | --- | --- | --- | --- | --- | --- | --- | --- | --- | --- |
| rs10054208 | 0.1187 | 0.0185 | 1.49E-10 | 0.6383 | T | C | -0.0022 | 0.0159 | 0.8882 | 757601 |
| rs10062049 | 0.2208 | 0.0255 | 4.50E-18 | 0.8641 | T | C | -0.0418 | 0.0209 | 0.04562 | 757601 |
| rs1006545 | 0.3633 | 0.0275 | 7.96E-40 | 0.1125 | T | G | 0.0045 | 0.0227 | 0.842 | 757601 |
| rs10069690 | 0.1615 | 0.021 | 1.42E-14 | 0.7419 | T | C | -0.0322 | 0.0215 | 0.1336 | 757601 |
| rs10087280 | 0.1381 | 0.0232 | 2.54E-09 | 0.1683 | A | G | -0.0097 | 0.0185 | 0.6004 | 757601 |
| rs10164193 | -0.2196 | 0.0327 | 1.87E-11 | 0.0777 | T | G | 0.018 | 0.0266 | 0.4977 | 757601 |
| rs1035673 | 0.1625 | 0.0176 | 3.00E-20 | 0.6032 | T | C | -0.0107 | 0.0146 | 0.4631 | 757601 |
| rs1039897 | -0.1085 | 0.0183 | 3.26E-09 | 0.3497 | A | G | -9.00E-04 | 0.0152 | 0.9511 | 757601 |
| rs10424224 | 0.1042 | 0.0182 | 1.05E-08 | 0.6416 | T | C | 0.0219 | 0.0149 | 0.1418 | 757601 |
| rs1043809 | 0.159 | 0.0223 | 9.77E-13 | 0.1917 | T | C | -0.003 | 0.0184 | 0.8713 | 757601 |
| rs1044822 | -0.1334 | 0.0243 | 4.14E-08 | 0.8512 | T | C | 0.0304 | 0.0202 | 0.1321 | 757601 |
| rs10490923 | 0.1533 | 0.0262 | 5.02E-09 | 0.8743 | A | G | 0.0683 | 0.021 | 0.001147 | 757601 |
| rs10493408 | 0.1584 | 0.0255 | 5.09E-10 | 0.8669 | A | C | -0.0346 | 0.0207 | 0.09511 | 757601 |
| rs10500932 | 0.2784 | 0.0333 | 5.79E-17 | 0.9257 | A | G | -0.0417 | 0.0266 | 0.1172 | 757601 |
| rs10759697 | 0.1308 | 0.0173 | 3.94E-14 | 0.5094 | A | G | -0.0323 | 0.0142 | 0.02272 | 757601 |
| rs10776752 | 0.4573 | 0.033 | 1.25E-43 | 0.9195 | T | G | -0.0169 | 0.0284 | 0.5519 | 757601 |
| rs1077795 | 0.1987 | 0.0199 | 1.62E-23 | 0.2611 | A | G | -0.0135 | 0.0165 | 0.412 | 757601 |
| rs10804330 | 0.1331 | 0.0176 | 4.60E-14 | 0.4329 | T | C | 0.0073 | 0.0145 | 0.6139 | 757601 |
| rs10832586 | -0.3083 | 0.0216 | 2.53E-46 | 0.2016 | A | C | 0.0287 | 0.0179 | 0.1091 | 757601 |
| rs10838702 | 0.2375 | 0.0178 | 1.27E-40 | 0.6125 | T | G | 0.0783 | 0.0145 | 6.68E-08 | 757601 |
| rs10873612 | -0.1096 | 0.0179 | 9.51E-10 | 0.4039 | T | C | 0.0172 | 0.0155 | 0.267 | 757601 |
| rs10941043 | -0.1269 | 0.019 | 2.52E-11 | 0.2906 | T | G | 0.0059 | 0.0157 | 0.7062 | 757601 |
| rs10980408 | -0.3745 | 0.0477 | 4.17E-15 | 0.0358 | T | C | -0.0382 | 0.0383 | 0.319 | 757601 |
| rs11040503 | -0.17 | 0.0236 | 5.65E-13 | 0.8143 | A | C | 0.0362 | 0.0202 | 0.07353 | 757601 |
| rs11070245 | -0.1287 | 0.0174 | 1.57E-13 | 0.5321 | T | G | 0.0046 | 0.0143 | 0.7479 | 757601 |
| rs11077961 | 0.1073 | 0.0186 | 8.55E-09 | 0.3676 | A | G | -0.011 | 0.016 | 0.4912 | 757601 |
| rs11108209 | -0.1901 | 0.03 | 2.40E-10 | 0.0932 | T | C | 0.048 | 0.025 | 0.05493 | 757601 |
| rs11141731 | -0.1258 | 0.0207 | 1.31E-09 | 0.772 | T | C | -0.0244 | 0.0175 | 0.1639 | 757601 |
| rs1114347 | -0.1792 | 0.0173 | 3.32E-25 | 0.4823 | A | G | 0.0196 | 0.0143 | 0.1712 | 757601 |
| rs11145807 | 0.155 | 0.0184 | 4.10E-17 | 0.5942 | A | G | -0.0019 | 0.0154 | 0.9002 | 757601 |
| rs11153730 | 0.1551 | 0.0173 | 2.57E-19 | 0.4906 | T | C | -0.0278 | 0.0142 | 0.05006 | 757601 |
| rs11228613 | 0.1741 | 0.0212 | 2.10E-16 | 0.2163 | T | G | 0.0064 | 0.0173 | 0.7112 | 757601 |
| rs11252324 | -0.2339 | 0.0328 | 1.03E-12 | 0.923 | T | G | 0.0238 | 0.0259 | 0.3568 | 757601 |
| rs1133400 | -0.1318 | 0.0215 | 8.30E-10 | 0.2148 | A | G | -0.0456 | 0.0178 | 0.01049 | 757601 |
| rs114503346 | -0.2678 | 0.0426 | 3.10E-10 | 0.9539 | T | C | -0.0026 | 0.0395 | 0.9469 | 757601 |
| rs115447786 | 0.2904 | 0.0455 | 1.75E-10 | 0.9573 | T | C | -0.1222 | 0.0438 | 0.005284 | 757601 |
| rs11556924 | -0.181 | 0.0181 | 1.83E-23 | 0.6173 | T | C | -0.0022 | 0.0159 | 0.8885 | 757601 |
| rs11592107 | 0.1203 | 0.0187 | 1.23E-10 | 0.6906 | A | G | 0.0098 | 0.0154 | 0.5265 | 757601 |
| rs116063464 | 0.2017 | 0.0369 | 4.68E-08 | 0.9399 | A | G | -0.0245 | 0.0308 | 0.426 | 757601 |
| rs11636952 | 0.3997 | 0.0189 | 5.21E-99 | 0.6869 | T | C | -0.0212 | 0.0153 | 0.1673 | 757601 |
| rs11661473 | 0.2007 | 0.0196 | 1.54E-24 | 0.7317 | A | G | -7.00E-04 | 0.0161 | 0.9657 | 757601 |
| rs11684340 | 0.1249 | 0.021 | 2.75E-09 | 0.2176 | A | C | 0.0054 | 0.0176 | 0.7576 | 757601 |
| rs11687089 | 0.1739 | 0.0175 | 2.79E-23 | 0.4171 | T | C | 0.0104 | 0.0145 | 0.47 | 757601 |
| rs11692619 | -0.1281 | 0.0184 | 3.31E-12 | 0.6393 | T | C | 0.0164 | 0.0154 | 0.2854 | 757601 |
| rs11721984 | -0.1409 | 0.0177 | 1.89E-15 | 0.5468 | T | C | 0.0062 | 0.0147 | 0.6732 | 757601 |
| rs11778153 | 0.1192 | 0.0182 | 5.84E-11 | 0.3569 | T | C | 0.016 | 0.015 | 0.2871 | 757601 |
| rs1178979 | 0.1504 | 0.0221 | 9.96E-12 | 0.1953 | T | C | 0.0065 | 0.0183 | 0.7201 | 757601 |
| rs11859505 | -0.1037 | 0.0181 | 9.76E-09 | 0.5805 | A | G | 0.0061 | 0.015 | 0.6815 | 757601 |
| rs11923343 | -0.1138 | 0.0181 | 3.10E-10 | 0.6396 | A | G | 0.0153 | 0.0146 | 0.2972 | 757601 |
| rs11945489 | -0.1392 | 0.0192 | 3.99E-13 | 0.7091 | T | C | 0.0031 | 0.0158 | 0.8421 | 757601 |
| rs11960210 | 0.2474 | 0.018 | 3.36E-43 | 0.3751 | T | C | 0.0148 | 0.0145 | 0.3061 | 757601 |
| rs11961593 | -0.3158 | 0.0349 | 1.49E-19 | 0.9315 | T | C | -0.0297 | 0.0282 | 0.2917 | 757601 |
| rs12088448 | -0.1544 | 0.0182 | 2.53E-17 | 0.356 | A | C | 0.0093 | 0.0154 | 0.5472 | 757601 |
| rs12148044 | 0.1371 | 0.023 | 2.66E-09 | 0.8266 | A | G | 0.0225 | 0.0187 | 0.2292 | 757601 |
| rs12152463 | 0.1006 | 0.0174 | 8.02E-09 | 0.5749 | T | C | 0.0038 | 0.0145 | 0.7939 | 757601 |
| rs1215469 | -0.1383 | 0.0211 | 5.23E-11 | 0.7705 | A | C | 0.0028 | 0.0175 | 0.8743 | 757601 |
| rs12216886 | 0.1292 | 0.0221 | 4.76E-09 | 0.1923 | T | G | 0.0058 | 0.0188 | 0.7584 | 757601 |
| rs12229480 | 0.1359 | 0.0193 | 2.11E-12 | 0.2775 | T | C | 0.0295 | 0.0159 | 0.06328 | 757601 |
| rs12247028 | -0.1396 | 0.0188 | 1.18E-13 | 0.3678 | A | G | 0.003 | 0.0154 | 0.8433 | 757601 |
| rs12337056 | 0.1364 | 0.0228 | 2.18E-09 | 0.8239 | T | C | 0.0204 | 0.0201 | 0.3112 | 757601 |
| rs12405515 | -0.1698 | 0.0174 | 1.92E-22 | 0.4298 | T | G | -8.00E-04 | 0.0144 | 0.9538 | 757601 |
| rs1243876 | -0.1063 | 0.019 | 2.14E-08 | 0.2988 | T | C | 0.0028 | 0.0156 | 0.8579 | 757601 |
| rs12444212 | 0.1291 | 0.0226 | 1.06E-08 | 0.1826 | T | C | 0.0122 | 0.0193 | 0.5277 | 757601 |
| rs12446456 | -0.181 | 0.0175 | 3.99E-25 | 0.5727 | T | C | -0.0377 | 0.0144 | 0.008659 | 757601 |
| rs12503341 | -0.2993 | 0.0462 | 9.43E-11 | 0.9606 | A | G | 0.0356 | 0.0352 | 0.3123 | 757601 |
| rs12509595 | -0.4972 | 0.0192 | 1.58E-148 | 0.2924 | T | C | 0.0216 | 0.016 | 0.1766 | 757601 |
| rs12515541 | 0.1156 | 0.0177 | 6.23E-11 | 0.3928 | T | G | 0.0128 | 0.0146 | 0.3832 | 757601 |
| rs12574332 | 0.2072 | 0.0266 | 6.14E-15 | 0.8773 | T | C | 0.0427 | 0.0225 | 0.05811 | 757601 |
| rs12596630 | 0.2606 | 0.0314 | 1.03E-16 | 0.9095 | T | C | -0.021 | 0.0259 | 0.4174 | 757601 |
| rs12601936 | -0.1429 | 0.0178 | 1.07E-15 | 0.6107 | A | G | 0.028 | 0.0146 | 0.05592 | 757601 |
| rs1263671 | -0.1394 | 0.0238 | 4.69E-09 | 0.1632 | T | C | -5.00E-04 | 0.02 | 0.9804 | 757601 |
| rs1265842 | 0.1113 | 0.0174 | 1.70E-10 | 0.5166 | T | C | -0.0288 | 0.0145 | 0.04639 | 757601 |
| rs12693302 | -0.2378 | 0.0181 | 2.16E-39 | 0.3482 | A | G | 0.0095 | 0.015 | 0.5253 | 757601 |
| rs12728150 | -0.2045 | 0.0318 | 1.28E-10 | 0.081 | A | G | -0.0309 | 0.0263 | 0.24 | 757601 |
| rs1275988 | -0.2945 | 0.0177 | 1.92E-62 | 0.3889 | T | C | -0.0144 | 0.0147 | 0.3281 | 757601 |
| rs12790943 | -0.1002 | 0.0175 | 1.14E-08 | 0.5787 | T | C | -0.0189 | 0.0145 | 0.1911 | 757601 |
| rs12866098 | 0.1033 | 0.0186 | 2.73E-08 | 0.6577 | A | G | -0.0127 | 0.0154 | 0.4103 | 757601 |
| rs12906962 | -0.2378 | 0.0188 | 8.73E-37 | 0.3233 | T | C | 0.005 | 0.0156 | 0.7485 | 757601 |
| rs12919839 | -0.1098 | 0.0192 | 1.04E-08 | 0.7159 | T | C | 0.0016 | 0.0158 | 0.9186 | 757601 |
| rs12929303 | 0.1572 | 0.0174 | 1.58E-19 | 0.4675 | A | G | -0.0043 | 0.0143 | 0.7644 | 757601 |
| rs12990959 | -0.1271 | 0.0187 | 1.11E-11 | 0.3125 | T | C | -0.0191 | 0.0154 | 0.2135 | 757601 |
| rs13001283 | 0.1522 | 0.0239 | 1.92E-10 | 0.8404 | A | G | -0.0483 | 0.0204 | 0.01772 | 757601 |
| rs13042148 | -0.1674 | 0.0244 | 7.24E-12 | 0.8463 | T | C | -0.0175 | 0.0208 | 0.3999 | 757601 |
| rs13107325 | -0.6747 | 0.0339 | 3.72E-88 | 0.9258 | T | C | 0.0202 | 0.0273 | 0.4607 | 757601 |
| rs13118687 | -0.1496 | 0.0175 | 1.37E-17 | 0.5298 | A | G | -0.0212 | 0.0146 | 0.1466 | 757601 |
| rs13124515 | -0.1052 | 0.0187 | 1.98E-08 | 0.6869 | T | C | -0.0072 | 0.0154 | 0.6387 | 757601 |
| rs13139571 | -0.2408 | 0.0203 | 2.29E-32 | 0.7634 | A | C | -0.0127 | 0.0165 | 0.4423 | 757601 |
| rs13152154 | -0.1186 | 0.0195 | 1.23E-09 | 0.2707 | T | C | -0.0082 | 0.0161 | 0.6091 | 757601 |
| rs13215166 | -0.3094 | 0.0174 | 1.79E-70 | 0.4415 | A | G | -0.0032 | 0.0143 | 0.8227 | 757601 |
| rs1322639 | -0.1584 | 0.0209 | 3.87E-14 | 0.2234 | A | G | -0.0126 | 0.0175 | 0.4731 | 757601 |
| rs13237249 | 0.1366 | 0.0177 | 1.03E-14 | 0.602 | T | C | 0.0266 | 0.0148 | 0.07244 | 757601 |
| rs13240040 | 0.1186 | 0.019 | 3.98E-10 | 0.3164 | A | G | 0.0034 | 0.0159 | 0.8316 | 757601 |
| rs1327235 | -0.3018 | 0.0173 | 4.76E-68 | 0.4714 | A | G | 0.0017 | 0.0143 | 0.9031 | 757601 |
| rs13355146 | 0.1224 | 0.0178 | 6.39E-12 | 0.6168 | T | C | 0.0115 | 0.0148 | 0.4366 | 757601 |
| rs13358657 | -0.224 | 0.0255 | 1.70E-18 | 0.1332 | A | G | -0.0274 | 0.0213 | 0.1986 | 757601 |
| rs134041 | 0.1223 | 0.0175 | 3.05E-12 | 0.564 | T | C | -0.0023 | 0.0144 | 0.8731 | 757601 |
| rs138420351 | 0.5568 | 0.0854 | 7.11E-11 | 0.984 | T | C | -0.1362 | 0.0715 | 0.05678 | 757601 |
| rs142449193 | -0.2573 | 0.0426 | 1.51E-09 | 0.954 | T | C | -0.0135 | 0.0339 | 0.6911 | 757601 |
| rs1425486 | -0.1331 | 0.0187 | 1.11E-12 | 0.6793 | T | C | 0.0159 | 0.0153 | 0.3012 | 757601 |
| rs1436138 | 0.1991 | 0.0182 | 7.33E-28 | 0.3633 | A | G | -0.016 | 0.0151 | 0.2885 | 757601 |
| rs147081004 | 0.1411 | 0.0257 | 4.09E-08 | 0.1444 | A | C | 0.0072 | 0.0217 | 0.7395 | 757601 |
| rs148401029 | -0.3122 | 0.0486 | 1.32E-10 | 0.9648 | A | C | 0.0369 | 0.0389 | 0.3439 | 757601 |
| rs1502358 | -0.1127 | 0.0185 | 1.13E-09 | 0.3187 | A | G | 0.0022 | 0.0152 | 0.8862 | 757601 |
| rs150816167 | -0.2873 | 0.0446 | 1.17E-10 | 0.0451 | T | C | -0.0093 | 0.0389 | 0.8108 | 757601 |
| rs1518460 | 0.1342 | 0.0189 | 1.26E-12 | 0.2918 | A | G | 0.0176 | 0.0157 | 0.2626 | 757601 |
| rs1582931 | 0.2161 | 0.0175 | 4.51E-35 | 0.5252 | A | G | 0.0241 | 0.0143 | 0.09116 | 757601 |
| rs1623474 | 0.2234 | 0.0184 | 6.24E-34 | 0.67 | T | C | -0.0203 | 0.0151 | 0.1788 | 757601 |
| rs1669907 | 0.1158 | 0.0191 | 1.36E-09 | 0.6968 | T | G | -0.0205 | 0.0162 | 0.2057 | 757601 |
| rs167479 | -0.362 | 0.0188 | 1.67E-82 | 0.5278 | T | G | 0.0159 | 0.0222 | 0.4738 | 757601 |
| rs1675383 | 0.1488 | 0.0174 | 1.47E-17 | 0.5569 | A | C | -0.0177 | 0.0144 | 0.2182 | 757601 |
| rs16853198 | 0.3386 | 0.0327 | 4.44E-25 | 0.0762 | A | G | 0.0042 | 0.0259 | 0.8723 | 757601 |
| rs1687295 | 0.2061 | 0.0194 | 2.99E-26 | 0.7296 | T | C | -0.0065 | 0.016 | 0.6832 | 757601 |
| rs16875357 | -0.1205 | 0.0203 | 2.70E-09 | 0.2431 | T | G | 0.0243 | 0.0167 | 0.1467 | 757601 |
| rs17321041 | 0.2313 | 0.0363 | 1.78E-10 | 0.9367 | T | C | -0.0561 | 0.0311 | 0.07142 | 757601 |
| rs17396055 | -0.115 | 0.0184 | 4.13E-10 | 0.6676 | A | G | -0.0078 | 0.0152 | 0.6092 | 757601 |
| rs17432462 | -0.1036 | 0.0179 | 7.31E-09 | 0.3766 | T | C | 0.0094 | 0.0148 | 0.5226 | 757601 |
| rs1745417 | 0.1708 | 0.0173 | 4.69E-23 | 0.4807 | T | C | 0.0134 | 0.0143 | 0.3492 | 757601 |
| rs17454517 | 0.1216 | 0.0174 | 2.65E-12 | 0.5064 | A | G | -0.0016 | 0.0143 | 0.9104 | 757601 |
| rs17677603 | -0.2 | 0.0178 | 3.90E-29 | 0.3837 | A | G | -0.0102 | 0.0148 | 0.4927 | 757601 |
| rs17678552 | -0.1649 | 0.0182 | 1.33E-19 | 0.3439 | T | C | -0.0099 | 0.0152 | 0.5167 | 757601 |
| rs17832905 | 0.1923 | 0.0346 | 2.81E-08 | 0.9283 | A | C | -0.0354 | 0.0288 | 0.2188 | 757601 |
| rs17880989 | 0.4014 | 0.0591 | 1.11E-11 | 0.9741 | A | G | -0.0882 | 0.0536 | 0.09945 | 757601 |
| rs1790123 | 0.1991 | 0.0218 | 6.87E-20 | 0.1968 | T | C | 0.0297 | 0.0178 | 0.09487 | 757601 |
| rs1819663 | 0.1147 | 0.0174 | 4.63E-11 | 0.4929 | A | G | 0.0095 | 0.0143 | 0.5067 | 757601 |
| rs1848510 | 0.1256 | 0.0181 | 4.10E-12 | 0.6377 | A | G | 0.0093 | 0.0148 | 0.533 | 757601 |
| rs1867624 | 0.1412 | 0.0178 | 2.08E-15 | 0.3853 | T | C | 0.0125 | 0.0147 | 0.3924 | 757601 |
| rs1871190 | 0.1078 | 0.0186 | 6.63E-09 | 0.6656 | T | G | -0.0033 | 0.0153 | 0.8282 | 757601 |
| rs1876490 | 0.1364 | 0.0192 | 1.16E-12 | 0.2833 | A | G | -0.0133 | 0.0159 | 0.4005 | 757601 |
| rs1882961 | 0.1272 | 0.0188 | 1.40E-11 | 0.6912 | T | C | 0.0045 | 0.0156 | 0.7731 | 757601 |
| rs1889785 | 0.1255 | 0.0174 | 5.61E-13 | 0.5449 | A | G | -0.0083 | 0.0143 | 0.5624 | 757601 |
| rs1903752 | -0.0987 | 0.0178 | 3.20E-08 | 0.4614 | T | C | 0.0149 | 0.015 | 0.3191 | 757601 |
| rs1906672 | 0.1402 | 0.0205 | 8.48E-12 | 0.7676 | A | G | -0.0097 | 0.0172 | 0.5745 | 757601 |
| rs1950500 | 0.1396 | 0.019 | 2.20E-13 | 0.7081 | T | C | 0.0084 | 0.0157 | 0.5908 | 757601 |
| rs1984195 | 0.1736 | 0.0173 | 1.43E-23 | 0.5117 | A | G | 0.0194 | 0.0142 | 0.1728 | 757601 |
| rs198851 | 0.3889 | 0.0244 | 2.93E-57 | 0.8504 | T | G | -0.0385 | 0.0198 | 0.0524 | 757601 |
| rs2009733 | 0.1217 | 0.0176 | 5.10E-12 | 0.5005 | A | G | 0.0112 | 0.0151 | 0.4571 | 757601 |
| rs2133386 | -0.1322 | 0.0176 | 5.21E-14 | 0.5673 | A | C | 0.009 | 0.0145 | 0.5337 | 757601 |
| rs2146315 | -0.1197 | 0.0205 | 5.03E-09 | 0.7682 | T | C | 0.0295 | 0.0171 | 0.0851 | 757601 |
| rs2191046 | 0.1184 | 0.0197 | 1.78E-09 | 0.2646 | T | G | -0.0137 | 0.0166 | 0.4099 | 757601 |
| rs2236295 | -0.207 | 0.0177 | 1.42E-31 | 0.6008 | T | G | 0.0301 | 0.0148 | 0.04159 | 757601 |
| rs2239268 | 0.1097 | 0.019 | 7.40E-09 | 0.2995 | A | G | -0.0149 | 0.0154 | 0.3316 | 757601 |
| rs2239917 | 0.1731 | 0.0176 | 9.69E-23 | 0.5748 | T | C | 0.0015 | 0.0146 | 0.9177 | 757601 |
| rs2271139 | -0.1247 | 0.0192 | 8.23E-11 | 0.714 | A | C | 5.00E-04 | 0.016 | 0.9764 | 757601 |
| rs2273654 | 0.1165 | 0.0175 | 2.75E-11 | 0.4392 | T | C | 0.0443 | 0.0144 | 0.002111 | 757601 |
| rs227426 | 0.1119 | 0.0175 | 1.75E-10 | 0.4381 | T | G | -0.0102 | 0.0143 | 0.4769 | 757601 |
| rs2306363 | -0.2643 | 0.0216 | 1.63E-34 | 0.7952 | T | G | 0.0109 | 0.0179 | 0.5412 | 757601 |
| rs2307111 | -0.1742 | 0.0178 | 1.62E-22 | 0.3966 | T | C | -0.005 | 0.0145 | 0.7292 | 757601 |
| rs234623 | -0.1191 | 0.0174 | 8.56E-12 | 0.4959 | A | G | 0.0128 | 0.0145 | 0.3768 | 757601 |
| rs2376997 | -0.1389 | 0.0218 | 1.94E-10 | 0.7511 | A | C | -0.0124 | 0.0164 | 0.4511 | 757601 |
| rs2397060 | -0.161 | 0.0251 | 1.46E-10 | 0.1405 | T | C | 0.0289 | 0.0201 | 0.1508 | 757601 |
| rs2421200 | -0.1097 | 0.0173 | 2.59E-10 | 0.5118 | T | G | 0.0067 | 0.0145 | 0.6442 | 757601 |
| rs2442618 | -0.1315 | 0.0177 | 1.21E-13 | 0.4277 | T | C | -0.0028 | 0.0151 | 0.8539 | 757601 |
| rs2444769 | 0.158 | 0.0219 | 4.85E-13 | 0.2051 | A | C | -0.0059 | 0.0178 | 0.7396 | 757601 |
| rs2469141 | 0.1351 | 0.0238 | 1.39E-08 | 0.1628 | T | C | -0.0273 | 0.0192 | 0.1545 | 757601 |
| rs2484294 | 0.3165 | 0.0196 | 1.17E-58 | 0.2673 | A | G | 0.0051 | 0.016 | 0.7512 | 757601 |
| rs2487926 | 0.0972 | 0.0176 | 3.31E-08 | 0.4295 | A | G | 0.0161 | 0.0146 | 0.2701 | 757601 |
| rs2493296 | 0.2496 | 0.0254 | 7.45E-23 | 0.8581 | T | C | -0.0254 | 0.0206 | 0.2172 | 757601 |
| rs2548459 | -0.132 | 0.0176 | 5.95E-14 | 0.5195 | T | C | -0.0212 | 0.0147 | 0.149 | 757601 |
| rs2569882 | 0.1199 | 0.0182 | 4.28E-11 | 0.4342 | T | C | -0.0067 | 0.0162 | 0.6796 | 757601 |
| rs2586970 | -0.1493 | 0.0175 | 1.56E-17 | 0.5639 | A | G | -0.0266 | 0.0143 | 0.0626 | 757601 |
| rs2589218 | -0.1207 | 0.0196 | 6.90E-10 | 0.2698 | T | C | -0.0101 | 0.0161 | 0.5293 | 757601 |
| rs2598 | 0.1387 | 0.0175 | 1.94E-15 | 0.4674 | A | G | -0.012 | 0.0144 | 0.4048 | 757601 |
| rs2627313 | 0.151 | 0.0175 | 5.86E-18 | 0.5543 | T | C | 0.0276 | 0.0143 | 0.05318 | 757601 |
| rs2643826 | 0.1857 | 0.0175 | 2.83E-26 | 0.5492 | T | C | 0.0163 | 0.0146 | 0.2646 | 757601 |
| rs2681485 | 0.2945 | 0.0176 | 1.31E-62 | 0.4024 | A | G | 0.0077 | 0.0144 | 0.5919 | 757601 |
| rs2744133 | 0.1435 | 0.0193 | 1.17E-13 | 0.2749 | A | G | 0.0096 | 0.0159 | 0.5486 | 757601 |
| rs28377357 | -0.1243 | 0.019 | 6.03E-11 | 0.7062 | A | G | 0.0255 | 0.0158 | 0.1075 | 757601 |
| rs28429256 | 0.1636 | 0.0188 | 2.83E-18 | 0.6656 | A | G | 0.0319 | 0.0159 | 0.04475 | 757601 |
| rs28544928 | 0.1543 | 0.0199 | 9.13E-15 | 0.2535 | T | G | -0.0038 | 0.0165 | 0.82 | 757601 |
| rs28570096 | 0.1396 | 0.0188 | 1.15E-13 | 0.6906 | T | C | 0.0085 | 0.0155 | 0.5833 | 757601 |
| rs28661492 | -0.1359 | 0.0222 | 9.56E-10 | 0.7978 | T | C | 0.0053 | 0.0187 | 0.7764 | 757601 |
| rs28675079 | -0.1444 | 0.0222 | 8.34E-11 | 0.8133 | A | G | -0.0014 | 0.0184 | 0.939 | 757601 |
| rs2906152 | -0.1873 | 0.0181 | 5.55E-25 | 0.3696 | A | G | -0.009 | 0.0151 | 0.5489 | 757601 |
| rs2921604 | -0.096 | 0.0176 | 4.46E-08 | 0.4633 | T | C | 0.0204 | 0.0145 | 0.1612 | 757601 |
| rs2925345 | 0.189 | 0.0174 | 1.60E-27 | 0.5324 | T | C | -0.0129 | 0.0146 | 0.3788 | 757601 |
| rs2957468 | 0.1377 | 0.0185 | 8.43E-14 | 0.6646 | A | G | -6.00E-04 | 0.0153 | 0.9673 | 757601 |
| rs2978098 | 0.1548 | 0.0176 | 1.33E-18 | 0.4535 | A | C | -0.0344 | 0.0145 | 0.01805 | 757601 |
| rs3006583 | -0.1303 | 0.0222 | 4.66E-09 | 0.1886 | T | C | -0.0488 | 0.018 | 0.006792 | 757601 |
| rs311564 | -0.133 | 0.0183 | 4.23E-13 | 0.6539 | A | G | -0.0127 | 0.0152 | 0.4027 | 757601 |
| rs3117736 | 0.2374 | 0.0196 | 9.71E-34 | 0.7339 | T | C | -0.018 | 0.0162 | 0.2659 | 757601 |
| rs34130368 | -0.2027 | 0.0284 | 8.77E-13 | 0.8828 | T | G | 4.00E-04 | 0.0244 | 0.9872 | 757601 |
| rs342977 | -0.1577 | 0.0205 | 1.67E-14 | 0.2285 | A | G | -0.0068 | 0.0171 | 0.6898 | 757601 |
| rs34487963 | -0.5734 | 0.0712 | 8.18E-16 | 0.9815 | A | C | 0.0263 | 0.06 | 0.6607 | 757601 |
| rs34517439 | -0.2514 | 0.0279 | 2.02E-19 | 0.8801 | A | C | -0.0028 | 0.0255 | 0.9114 | 757601 |
| rs34645159 | -0.133 | 0.0174 | 2.07E-14 | 0.4987 | A | G | 0.0099 | 0.0143 | 0.4891 | 757601 |
| rs347585 | 0.1506 | 0.0189 | 1.57E-15 | 0.2986 | T | C | -0.0247 | 0.0154 | 0.1092 | 757601 |
| rs35091929 | 0.1828 | 0.0177 | 6.46E-25 | 0.6032 | T | C | -0.0069 | 0.0148 | 0.6384 | 757601 |
| rs35213536 | 0.2044 | 0.0205 | 2.54E-23 | 0.7533 | T | G | -0.0318 | 0.0171 | 0.06261 | 757601 |
| rs35261542 | 0.1196 | 0.0195 | 9.29E-10 | 0.7321 | A | C | -0.0091 | 0.0161 | 0.5712 | 757601 |
| rs35413927 | -0.1274 | 0.0189 | 1.77E-11 | 0.3049 | A | G | 0.0195 | 0.0156 | 0.2114 | 757601 |
| rs35506078 | -0.1348 | 0.0183 | 1.54E-13 | 0.3366 | T | C | 0.005 | 0.0153 | 0.7417 | 757601 |
| rs35927325 | 0.2221 | 0.0364 | 1.01E-09 | 0.9386 | T | C | -0.0082 | 0.03 | 0.786 | 757601 |
| rs360153 | -0.2198 | 0.0175 | 4.37E-36 | 0.5828 | T | C | 0.015 | 0.0145 | 0.2994 | 757601 |
| rs36117336 | -0.147 | 0.0198 | 1.10E-13 | 0.2562 | T | C | 0.0079 | 0.0165 | 0.6333 | 757601 |
| rs3735533 | -0.487 | 0.0331 | 6.32E-49 | 0.9258 | T | C | -0.0173 | 0.0277 | 0.5309 | 757601 |
| rs3743111 | 0.1517 | 0.0178 | 1.62E-17 | 0.387 | A | G | 0.0039 | 0.0147 | 0.7904 | 757601 |
| rs3743369 | 0.104 | 0.0179 | 6.82E-09 | 0.3722 | A | G | -3.00E-04 | 0.0148 | 0.9844 | 757601 |
| rs3772219 | 0.1754 | 0.0185 | 2.94E-21 | 0.3193 | A | C | -0.0441 | 0.0155 | 0.004517 | 757601 |
| rs3774702 | 0.147 | 0.0228 | 1.18E-10 | 0.8232 | A | G | 0.0039 | 0.0186 | 0.8341 | 757601 |
| rs3776299 | 0.1266 | 0.0175 | 5.06E-13 | 0.5441 | A | G | -0.0057 | 0.0142 | 0.6887 | 757601 |
| rs3785837 | 0.1453 | 0.0213 | 9.57E-12 | 0.2365 | A | G | 0.0031 | 0.0173 | 0.8587 | 757601 |
| rs3798293 | -0.1328 | 0.021 | 2.70E-10 | 0.2165 | A | G | 0.0211 | 0.0171 | 0.2173 | 757601 |
| rs3802230 | -0.1605 | 0.0174 | 2.75E-20 | 0.4554 | A | C | 0.0132 | 0.0143 | 0.3547 | 757601 |
| rs3807101 | -0.1743 | 0.0265 | 4.57E-11 | 0.877 | T | C | -0.0261 | 0.0218 | 0.2329 | 757601 |
| rs3861113 | 0.2126 | 0.0322 | 3.95E-11 | 0.9175 | A | C | -0.0146 | 0.0256 | 0.5679 | 757601 |
| rs3864004 | 0.1004 | 0.0173 | 6.28E-09 | 0.5315 | A | G | 0.0047 | 0.0144 | 0.7423 | 757601 |
| rs387865 | -0.1059 | 0.0191 | 3.17E-08 | 0.6938 | T | C | 0.0106 | 0.016 | 0.5062 | 757601 |
| rs3916033 | -0.1233 | 0.0185 | 2.42E-11 | 0.4435 | T | C | 0.0013 | 0.0152 | 0.9307 | 757601 |
| rs3918226 | 0.6117 | 0.0329 | 5.31E-77 | 0.9187 | T | C | -0.0337 | 0.0284 | 0.2361 | 757601 |
| rs3943093 | 0.2477 | 0.0184 | 3.95E-41 | 0.6766 | T | C | 0.0247 | 0.0151 | 0.1026 | 757601 |
| rs4074812 | -0.1336 | 0.0175 | 2.07E-14 | 0.4465 | A | G | -3.00E-04 | 0.0143 | 0.9838 | 757601 |
| rs4077158 | -0.1832 | 0.0173 | 3.09E-26 | 0.5286 | T | C | -0.0024 | 0.0143 | 0.8665 | 757601 |
| rs4102481 | -0.1248 | 0.019 | 4.87E-11 | 0.3049 | T | G | 0.0022 | 0.0155 | 0.8888 | 757601 |
| rs4141663 | -0.1496 | 0.0175 | 1.41E-17 | 0.5784 | T | C | -0.0242 | 0.0144 | 0.09278 | 757601 |
| rs4362428 | -0.1127 | 0.0176 | 1.45E-10 | 0.5913 | A | C | -0.0321 | 0.0143 | 0.02525 | 757601 |
| rs440454 | -0.2602 | 0.0192 | 7.52E-42 | 0.684 | A | G | 0.024 | 0.0163 | 0.1402 | 757601 |
| rs4424827 | -0.0981 | 0.0175 | 2.11E-08 | 0.4331 | T | C | 0.0025 | 0.0143 | 0.8619 | 757601 |
| rs4507125 | -0.1244 | 0.0211 | 3.60E-09 | 0.2136 | A | C | -0.0341 | 0.0174 | 0.04993 | 757601 |
| rs45474499 | 0.3562 | 0.0415 | 8.50E-18 | 0.9527 | T | C | -0.0254 | 0.0335 | 0.4494 | 757601 |
| rs4556017 | -0.1601 | 0.0247 | 9.67E-11 | 0.1476 | T | C | 0.0193 | 0.0209 | 0.3546 | 757601 |
| rs4615669 | -0.114 | 0.0174 | 6.10E-11 | 0.4403 | A | G | -0.0313 | 0.0152 | 0.03996 | 757601 |
| rs4645335 | 0.1142 | 0.0185 | 7.04E-10 | 0.664 | A | G | -0.008 | 0.0154 | 0.6034 | 757601 |
| rs4651224 | 0.1102 | 0.0175 | 3.39E-10 | 0.5531 | T | C | 0.0149 | 0.0144 | 0.3007 | 757601 |
| rs4675682 | -0.1409 | 0.0173 | 4.49E-16 | 0.4622 | T | C | 0.0068 | 0.0143 | 0.6359 | 757601 |
| rs4704514 | 0.1087 | 0.0193 | 1.71E-08 | 0.7167 | T | C | -0.0049 | 0.016 | 0.7612 | 757601 |
| rs4722548 | -0.1346 | 0.0176 | 1.99E-14 | 0.3996 | T | C | -0.0118 | 0.0145 | 0.4154 | 757601 |
| rs4726006 | 0.1339 | 0.02 | 2.39E-11 | 0.7452 | A | G | -3.00E-04 | 0.0164 | 0.9837 | 757601 |
| rs4743021 | -0.108 | 0.0194 | 2.41E-08 | 0.3147 | T | C | 0.0068 | 0.0166 | 0.6839 | 757601 |
| rs4756782 | 0.1551 | 0.0234 | 3.52E-11 | 0.835 | A | C | 0.0196 | 0.0191 | 0.3058 | 757601 |
| rs4814837 | -0.1003 | 0.0184 | 4.62E-08 | 0.6576 | T | C | -0.0165 | 0.015 | 0.2716 | 757601 |
| rs4873492 | 0.1401 | 0.0231 | 1.28E-09 | 0.8275 | T | C | 0.0199 | 0.0185 | 0.2812 | 757601 |
| rs488834 | -0.1931 | 0.0208 | 1.94E-20 | 0.2359 | T | C | -0.0111 | 0.0173 | 0.5215 | 757601 |
| rs4891258 | -0.1159 | 0.0187 | 5.72E-10 | 0.3174 | A | G | 4.00E-04 | 0.0155 | 0.9771 | 757601 |
| rs4903064 | 0.1543 | 0.0206 | 7.84E-14 | 0.2355 | T | C | 0.0011 | 0.0167 | 0.9454 | 757601 |
| rs4912840 | -0.1485 | 0.0245 | 1.25E-09 | 0.8453 | A | G | 0.018 | 0.0202 | 0.3738 | 757601 |
| rs4926901 | 0.0984 | 0.018 | 4.82E-08 | 0.6452 | A | G | 5.00E-04 | 0.015 | 0.976 | 757601 |
| rs4926923 | 0.1918 | 0.0308 | 4.75E-10 | 0.0883 | T | C | 0.0274 | 0.025 | 0.2717 | 757601 |
| rs4932373 | -0.3664 | 0.0189 | 7.71E-84 | 0.3257 | A | C | -0.0052 | 0.0163 | 0.7484 | 757601 |
| rs4936099 | 0.1745 | 0.0178 | 1.16E-22 | 0.4011 | A | C | -0.0151 | 0.0149 | 0.3104 | 757601 |
| rs4948643 | 0.1591 | 0.0194 | 2.26E-16 | 0.718 | T | C | -0.0037 | 0.0158 | 0.8167 | 757601 |
| rs4952668 | -0.192 | 0.018 | 1.13E-26 | 0.3763 | A | G | -0.0091 | 0.015 | 0.5446 | 757601 |
| rs4954192 | -0.1225 | 0.0179 | 8.15E-12 | 0.6128 | T | C | 0.0378 | 0.0146 | 0.009791 | 757601 |
| rs5010183 | 0.1195 | 0.018 | 2.86E-11 | 0.372 | T | C | 0.0071 | 0.0149 | 0.635 | 757601 |
| rs504217 | 0.2745 | 0.0335 | 2.51E-16 | 0.9264 | T | C | 0.0295 | 0.0274 | 0.2818 | 757601 |
| rs504691 | -0.1177 | 0.0177 | 3.14E-11 | 0.5998 | A | C | -0.004 | 0.0146 | 0.7822 | 757601 |
| rs507666 | -0.2854 | 0.0223 | 2.27E-37 | 0.8128 | A | G | -0.0112 | 0.0179 | 0.532 | 757601 |
| rs509067 | -0.1436 | 0.0175 | 2.65E-16 | 0.5863 | T | C | -0.0116 | 0.0144 | 0.42 | 757601 |
| rs521033 | -0.1802 | 0.0253 | 1.10E-12 | 0.1364 | A | G | -0.019 | 0.0201 | 0.3454 | 757601 |
| rs55684003 | 0.122 | 0.0189 | 1.01E-10 | 0.3041 | A | G | 0.0103 | 0.0155 | 0.5052 | 757601 |
| rs55747751 | -0.2239 | 0.0331 | 1.39E-11 | 0.919 | A | G | 0.0014 | 0.0296 | 0.963 | 757601 |
| rs55770741 | -0.1281 | 0.0175 | 2.20E-13 | 0.4387 | T | C | 0.0104 | 0.0147 | 0.4792 | 757601 |
| rs55857306 | -0.5224 | 0.0235 | 5.05E-109 | 0.8398 | A | G | 0.0569 | 0.0197 | 0.003821 | 757601 |
| rs55935819 | 0.1271 | 0.0181 | 1.96E-12 | 0.6364 | A | G | 0.0134 | 0.015 | 0.3723 | 757601 |
| rs55938136 | 0.1408 | 0.0225 | 4.21E-10 | 0.2249 | A | G | 0.0393 | 0.0179 | 0.02782 | 757601 |
| rs55944332 | -0.2365 | 0.0204 | 3.27E-31 | 0.2368 | A | G | 0.0081 | 0.017 | 0.6349 | 757601 |
| rs55993676 | -0.2097 | 0.0191 | 3.82E-28 | 0.7084 | T | G | -0.003 | 0.0161 | 0.8519 | 757601 |
| rs56256111 | 0.1926 | 0.0263 | 2.60E-13 | 0.8558 | A | G | 0.0174 | 0.024 | 0.4695 | 757601 |
| rs56345595 | 0.1329 | 0.0177 | 5.20E-14 | 0.4152 | A | G | 0.0201 | 0.0148 | 0.1748 | 757601 |
| rs569550 | -0.2688 | 0.0181 | 1.23E-49 | 0.3954 | T | G | 0.0191 | 0.0152 | 0.209 | 757601 |
| rs5753630 | 0.107 | 0.0175 | 8.76E-10 | 0.4382 | A | G | 0.0016 | 0.0144 | 0.9098 | 757601 |
| rs57708073 | 0.1907 | 0.0214 | 4.73E-19 | 0.2608 | A | G | -0.0069 | 0.0183 | 0.7072 | 757601 |
| rs58693787 | 0.1584 | 0.0202 | 3.82E-15 | 0.2458 | A | G | 0.0082 | 0.0165 | 0.6184 | 757601 |
| rs5992929 | 0.1684 | 0.0193 | 3.07E-18 | 0.7166 | T | C | 0.0206 | 0.0161 | 0.201 | 757601 |
| rs602521 | 0.1351 | 0.0195 | 3.97E-12 | 0.7344 | A | G | -0.0016 | 0.0161 | 0.922 | 757601 |
| rs6031431 | -0.1153 | 0.0175 | 4.94E-11 | 0.4622 | A | G | -0.0124 | 0.0146 | 0.3925 | 757601 |
| rs604723 | -0.3848 | 0.0194 | 2.32E-87 | 0.7247 | T | C | -0.001 | 0.0158 | 0.9477 | 757601 |
| rs6078393 | 0.1205 | 0.0176 | 7.66E-12 | 0.4106 | T | G | -0.0023 | 0.0146 | 0.8739 | 757601 |
| rs6108168 | -0.1901 | 0.0199 | 1.10E-21 | 0.7454 | A | C | 0.0105 | 0.0166 | 0.5263 | 757601 |
| rs61772592 | -0.1509 | 0.0261 | 7.42E-09 | 0.1257 | A | G | -0.016 | 0.0214 | 0.4557 | 757601 |
| rs61789369 | -0.3039 | 0.0436 | 3.07E-12 | 0.0435 | A | G | 0.0203 | 0.0368 | 0.5817 | 757601 |
| rs61917655 | 0.2246 | 0.0297 | 3.72E-14 | 0.8989 | T | C | -0.0013 | 0.0245 | 0.9578 | 757601 |
| rs61948065 | -0.1737 | 0.027 | 1.17E-10 | 0.1212 | A | C | -0.013 | 0.0233 | 0.577 | 757601 |
| rs62030049 | 0.1336 | 0.0209 | 1.55E-10 | 0.2404 | A | G | 0.004 | 0.0179 | 0.8245 | 757601 |
| rs62064603 | -0.1335 | 0.0229 | 5.52E-09 | 0.8144 | T | C | -0.0237 | 0.0183 | 0.1962 | 757601 |
| rs62155750 | -0.2177 | 0.0196 | 8.27E-29 | 0.3074 | A | G | -0.0091 | 0.0171 | 0.5928 | 757601 |
| rs62158170 | 0.1645 | 0.0211 | 6.63E-15 | 0.2166 | A | G | 0.0087 | 0.0176 | 0.6233 | 757601 |
| rs62234672 | 0.1248 | 0.0229 | 4.92E-08 | 0.8248 | A | C | -0.0194 | 0.0189 | 0.3048 | 757601 |
| rs62294352 | -0.1605 | 0.0223 | 6.07E-13 | 0.7843 | T | C | 0.0116 | 0.0187 | 0.5357 | 757601 |
| rs62301873 | -0.1734 | 0.0284 | 1.06E-09 | 0.1061 | A | G | 0.0476 | 0.0249 | 0.05573 | 757601 |
| rs62380354 | 0.1825 | 0.0291 | 3.68E-10 | 0.1096 | A | C | 9.00E-04 | 0.0257 | 0.9711 | 757601 |
| rs62413546 | -0.1877 | 0.032 | 4.58E-09 | 0.9153 | T | C | 0.0052 | 0.027 | 0.8475 | 757601 |
| rs62434124 | -0.4853 | 0.0338 | 7.83E-47 | 0.9289 | T | C | 0.0081 | 0.0285 | 0.7765 | 757601 |
| rs62503324 | 0.2033 | 0.0204 | 2.11E-23 | 0.7603 | T | C | 0.0269 | 0.0172 | 0.1182 | 757601 |
| rs6271 | -0.4313 | 0.0352 | 1.72E-34 | 0.9263 | T | C | -0.0258 | 0.037 | 0.4865 | 757601 |
| rs636202 | 0.1023 | 0.0174 | 4.40E-09 | 0.5185 | T | C | -0.004 | 0.0146 | 0.7858 | 757601 |
| rs6442105 | -0.2485 | 0.0185 | 3.10E-41 | 0.6726 | A | G | -0.0195 | 0.0153 | 0.2018 | 757601 |
| rs6445590 | 0.1284 | 0.0174 | 1.65E-13 | 0.5455 | A | G | 0.0098 | 0.0144 | 0.493 | 757601 |
| rs6464165 | -0.217 | 0.0195 | 7.34E-29 | 0.2809 | T | C | -0.0024 | 0.0163 | 0.8852 | 757601 |
| rs6487076 | 0.174 | 0.0209 | 8.69E-17 | 0.223 | A | G | -0.0022 | 0.0175 | 0.9021 | 757601 |
| rs6490019 | -0.1778 | 0.0178 | 2.10E-23 | 0.6203 | A | G | 0.0195 | 0.0147 | 0.1841 | 757601 |
| rs6504163 | -0.1842 | 0.0183 | 6.30E-24 | 0.3763 | T | C | 0.0474 | 0.0152 | 0.001788 | 757601 |
| rs6546810 | -0.12 | 0.0181 | 3.16E-11 | 0.3525 | T | C | -0.0017 | 0.0149 | 0.9116 | 757601 |
| rs6556384 | -0.152 | 0.0221 | 5.91E-12 | 0.1895 | A | C | -0.0178 | 0.0181 | 0.3253 | 757601 |
| rs6580970 | -0.1661 | 0.0191 | 4.03E-18 | 0.7013 | T | C | -0.0089 | 0.0159 | 0.5777 | 757601 |
| rs6581101 | -0.1261 | 0.0179 | 2.05E-12 | 0.3962 | A | C | -0.0091 | 0.015 | 0.5446 | 757601 |
| rs6602177 | -0.1203 | 0.0207 | 6.52E-09 | 0.2927 | T | C | 0.0103 | 0.0163 | 0.5287 | 757601 |
| rs66682451 | 0.1348 | 0.0194 | 3.44E-12 | 0.2747 | A | G | -0.0145 | 0.0159 | 0.364 | 757601 |
| rs6686889 | 0.1918 | 0.0199 | 6.95E-22 | 0.7467 | T | C | -0.0086 | 0.0164 | 0.6027 | 757601 |
| rs66887589 | -0.161 | 0.0174 | 1.83E-20 | 0.4779 | T | C | 0.0099 | 0.0143 | 0.4911 | 757601 |
| rs6763931 | 0.1383 | 0.0173 | 1.48E-15 | 0.5562 | A | G | -0.0169 | 0.0143 | 0.2366 | 757601 |
| rs6777317 | 0.1249 | 0.0195 | 1.51E-10 | 0.7101 | A | G | 0.024 | 0.0158 | 0.128 | 757601 |
| rs6779368 | -0.1791 | 0.0184 | 2.28E-22 | 0.3423 | A | G | -0.0153 | 0.0155 | 0.3221 | 757601 |
| rs6795735 | -0.1438 | 0.0176 | 3.05E-16 | 0.5891 | T | C | -0.0256 | 0.0145 | 0.07692 | 757601 |
| rs68085857 | 0.191 | 0.0205 | 9.83E-21 | 0.766 | T | C | -0.0213 | 0.0167 | 0.2042 | 757601 |
| rs682681 | -0.1454 | 0.0185 | 4.47E-15 | 0.6665 | T | C | 0.0036 | 0.0153 | 0.8141 | 757601 |
| rs6875967 | 0.1344 | 0.0181 | 1.21E-13 | 0.6479 | A | G | 0.0119 | 0.0153 | 0.4363 | 757601 |
| rs6905288 | 0.1759 | 0.0179 | 7.79E-23 | 0.4319 | A | G | -0.0072 | 0.0153 | 0.6389 | 757601 |
| rs6934891 | 0.1275 | 0.0177 | 5.21E-13 | 0.5745 | A | G | 0.0325 | 0.0147 | 0.02723 | 757601 |
| rs693974 | -0.1847 | 0.0177 | 1.76E-25 | 0.3964 | T | C | -0.0039 | 0.0147 | 0.7886 | 757601 |
| rs6959688 | -0.1269 | 0.0178 | 1.02E-12 | 0.4015 | A | G | 0.033 | 0.0148 | 0.02597 | 757601 |
| rs6983239 | 0.1159 | 0.0211 | 3.71E-08 | 0.7812 | T | G | -0.0067 | 0.0171 | 0.6957 | 757601 |
| rs699 | -0.2359 | 0.0177 | 1.30E-40 | 0.407 | A | G | 0.0314 | 0.0144 | 0.02953 | 757601 |
| rs7012891 | -0.1391 | 0.0205 | 1.20E-11 | 0.2367 | T | C | -0.0144 | 0.0165 | 0.3853 | 757601 |
| rs7106104 | -0.1186 | 0.0193 | 7.72E-10 | 0.281 | T | C | 0.009 | 0.0158 | 0.5669 | 757601 |
| rs710698 | 0.1059 | 0.0176 | 1.89E-09 | 0.4135 | A | G | 0.0028 | 0.0145 | 0.8453 | 757601 |
| rs7115331 | -0.1266 | 0.0192 | 3.92E-11 | 0.2857 | T | G | 0.0161 | 0.0156 | 0.3041 | 757601 |
| rs7137828 | -0.5027 | 0.0176 | 4.80E-180 | 0.4817 | T | C | 0.0216 | 0.0144 | 0.1334 | 757601 |
| rs7155504 | 0.2286 | 0.0317 | 5.16E-13 | 0.0876 | T | C | -0.0299 | 0.0249 | 0.2298 | 757601 |
| rs7169864 | -0.1132 | 0.0205 | 3.40E-08 | 0.7678 | T | C | 0.0287 | 0.0172 | 0.0938 | 757601 |
| rs7192407 | 0.1019 | 0.0174 | 4.53E-09 | 0.528 | T | C | 0.0118 | 0.0143 | 0.4097 | 757601 |
| rs7217916 | 0.1111 | 0.0179 | 5.63E-10 | 0.6146 | A | G | 0.0051 | 0.0148 | 0.7304 | 757601 |
| rs7227492 | 0.181 | 0.0227 | 1.43E-15 | 0.1822 | T | C | -0.0141 | 0.0187 | 0.4513 | 757601 |
| rs722783 | -0.2093 | 0.0208 | 9.03E-24 | 0.7784 | A | G | 0.0385 | 0.0171 | 0.02397 | 757601 |
| rs7257694 | 0.1837 | 0.0178 | 6.28E-25 | 0.5997 | T | C | 0.0185 | 0.0152 | 0.2237 | 757601 |
| rs7258382 | 0.2624 | 0.0248 | 3.03E-26 | 0.1611 | T | C | 0.0347 | 0.0204 | 0.08964 | 757601 |
| rs7265695 | 0.1967 | 0.0219 | 2.48E-19 | 0.1965 | T | C | -0.0248 | 0.0185 | 0.1799 | 757601 |
| rs72683923 | 0.5325 | 0.0635 | 5.02E-17 | 0.0212 | T | C | 0.1035 | 0.0504 | 0.03993 | 757601 |
| rs72719149 | -0.1279 | 0.0186 | 6.34E-12 | 0.3164 | T | C | -0.0143 | 0.0152 | 0.3459 | 757601 |
| rs7278003 | -0.1293 | 0.0176 | 1.78E-13 | 0.5615 | T | C | -0.0078 | 0.0144 | 0.5904 | 757601 |
| rs72831343 | 0.4936 | 0.0248 | 4.77E-88 | 0.1419 | T | G | -8.00E-04 | 0.0203 | 0.9703 | 757601 |
| rs72842207 | -0.2112 | 0.0211 | 1.10E-23 | 0.7851 | T | C | 0.0159 | 0.0175 | 0.3621 | 757601 |
| rs72976750 | -0.1718 | 0.0251 | 7.37E-12 | 0.1396 | T | C | -0.0112 | 0.0209 | 0.5934 | 757601 |
| rs72999033 | 0.2793 | 0.0358 | 5.95E-15 | 0.9342 | T | C | 0.0132 | 0.0298 | 0.6593 | 757601 |
| rs73033340 | 0.5312 | 0.0525 | 5.06E-24 | 0.0362 | A | G | -0.0506 | 0.05 | 0.3117 | 757601 |
| rs73046792 | -0.1518 | 0.0245 | 5.87E-10 | 0.8408 | A | G | -0.0222 | 0.0213 | 0.2965 | 757601 |
| rs7306947 | -0.205 | 0.0342 | 2.14E-09 | 0.072 | T | G | 0.0412 | 0.0287 | 0.1518 | 757601 |
| rs7321688 | 0.1507 | 0.0205 | 1.99E-13 | 0.7675 | A | C | 0.0049 | 0.0168 | 0.7724 | 757601 |
| rs7350752 | -0.1504 | 0.0268 | 1.97E-08 | 0.8759 | A | G | -0.0131 | 0.0229 | 0.5676 | 757601 |
| rs7427249 | -0.1098 | 0.0176 | 4.34E-10 | 0.42 | A | G | 0.0232 | 0.0145 | 0.1097 | 757601 |
| rs74439044 | -0.3496 | 0.0294 | 1.38E-32 | 0.0983 | T | C | -0.0291 | 0.0245 | 0.2351 | 757601 |
| rs7491960 | -0.1288 | 0.018 | 8.44E-13 | 0.508 | T | C | 0.0026 | 0.0145 | 0.8594 | 757601 |
| rs751984 | 0.3937 | 0.0275 | 1.38E-46 | 0.1174 | T | C | -0.0029 | 0.0232 | 0.8997 | 757601 |
| rs7524019 | 0.1036 | 0.0174 | 2.60E-09 | 0.508 | T | C | -0.0084 | 0.0144 | 0.5615 | 757601 |
| rs75511781 | -0.3721 | 0.047 | 2.45E-15 | 0.0425 | A | G | -0.059 | 0.0467 | 0.2068 | 757601 |
| rs75717699 | -0.4667 | 0.0541 | 6.71E-18 | 0.0305 | T | G | -0.0482 | 0.0533 | 0.3655 | 757601 |
| rs7572130 | -0.1796 | 0.0287 | 4.12E-10 | 0.1042 | A | G | 0.019 | 0.0232 | 0.4137 | 757601 |
| rs7592578 | -0.1998 | 0.0224 | 4.71E-19 | 0.8062 | T | G | -0.007 | 0.0181 | 0.699 | 757601 |
| rs7623706 | 0.0975 | 0.0176 | 2.84E-08 | 0.4349 | A | G | 0.0056 | 0.0144 | 0.6975 | 757601 |
| rs76326501 | 0.3618 | 0.0305 | 2.17E-32 | 0.0911 | A | C | -0.0114 | 0.0258 | 0.6584 | 757601 |
| rs76452347 | -0.2246 | 0.0229 | 9.37E-23 | 0.7947 | T | C | -0.0019 | 0.0199 | 0.9252 | 757601 |
| rs76719272 | -0.1438 | 0.0264 | 4.86E-08 | 0.8685 | T | C | -0.0569 | 0.023 | 0.0133 | 757601 |
| rs76785130 | -0.4285 | 0.0662 | 9.36E-11 | 0.0199 | A | G | 0.0335 | 0.0568 | 0.556 | 757601 |
| rs76954792 | 0.1213 | 0.0208 | 5.06E-09 | 0.7678 | T | C | -0.0267 | 0.0174 | 0.1247 | 757601 |
| rs77032376 | -0.173 | 0.0249 | 3.64E-12 | 0.8521 | T | C | -0.0057 | 0.021 | 0.7855 | 757601 |
| rs7737851 | -0.1256 | 0.022 | 1.11E-08 | 0.8056 | T | C | 0.0047 | 0.018 | 0.7936 | 757601 |
| rs7767235 | -0.1181 | 0.0182 | 7.95E-11 | 0.6468 | A | C | 0.0301 | 0.0151 | 0.04562 | 757601 |
| rs7788746 | -0.1644 | 0.0183 | 3.19E-19 | 0.3309 | T | G | 0.0427 | 0.0152 | 0.004935 | 757601 |
| rs77924615 | -0.3163 | 0.0224 | 3.72E-45 | 0.8018 | A | G | -0.0131 | 0.0189 | 0.4878 | 757601 |
| rs7800558 | 0.096 | 0.0175 | 4.46E-08 | 0.4219 | T | C | 0.0068 | 0.0145 | 0.6382 | 757601 |
| rs78151625 | -0.1869 | 0.0233 | 1.04E-15 | 0.1658 | T | C | -0.0322 | 0.0195 | 0.0981 | 757601 |
| rs786921 | -0.1145 | 0.0176 | 8.63E-11 | 0.4043 | A | G | 0.0031 | 0.0144 | 0.8271 | 757601 |
| rs78809139 | -0.2281 | 0.0288 | 2.58E-15 | 0.8986 | A | G | -0.0106 | 0.0244 | 0.6638 | 757601 |
| rs78909293 | 0.321 | 0.0429 | 7.31E-14 | 0.0449 | T | C | -0.0035 | 0.0346 | 0.9207 | 757601 |
| rs79208229 | 0.2128 | 0.0326 | 6.53E-11 | 0.9125 | T | G | -0.0816 | 0.0295 | 0.005631 | 757601 |
| rs7926335 | 0.1804 | 0.0195 | 2.05E-20 | 0.7301 | T | C | 0.0211 | 0.0161 | 0.1892 | 757601 |
| rs79286081 | -0.1631 | 0.0299 | 4.83E-08 | 0.8979 | A | G | 0.0228 | 0.0238 | 0.3366 | 757601 |
| rs7933758 | -0.1138 | 0.0191 | 2.58E-09 | 0.6953 | T | C | 0.016 | 0.0158 | 0.3112 | 757601 |
| rs7959649 | 0.1166 | 0.0202 | 8.14E-09 | 0.7576 | T | C | 0.002 | 0.0167 | 0.9033 | 757601 |
| rs7967705 | 0.2694 | 0.0178 | 1.54E-51 | 0.6196 | T | C | 0.0031 | 0.0146 | 0.8303 | 757601 |
| rs79889784 | -0.3941 | 0.0717 | 3.86E-08 | 0.9824 | T | G | 0.0466 | 0.0538 | 0.3869 | 757601 |
| rs7990017 | 0.1039 | 0.0185 | 1.92E-08 | 0.5267 | T | C | -0.0032 | 0.017 | 0.8529 | 757601 |
| rs7992292 | 0.1367 | 0.0231 | 3.19E-09 | 0.176 | A | G | 0.0085 | 0.0188 | 0.6518 | 757601 |
| rs80095680 | -0.1566 | 0.0198 | 2.81E-15 | 0.2633 | A | G | 0.0352 | 0.0165 | 0.03301 | 757601 |
| rs8014182 | -0.1942 | 0.0257 | 3.94E-14 | 0.8681 | T | C | -0.0442 | 0.0209 | 0.03428 | 757601 |
| rs8046697 | -0.1289 | 0.0179 | 6.10E-13 | 0.5833 | T | C | 0.0153 | 0.015 | 0.3059 | 757601 |
| rs8108717 | 0.1323 | 0.0179 | 1.39E-13 | 0.6084 | A | G | -0.0146 | 0.0149 | 0.3262 | 757601 |
| rs824523 | 0.1226 | 0.0183 | 2.26E-11 | 0.6656 | A | C | -0.0032 | 0.015 | 0.8305 | 757601 |
| rs881858 | 0.1553 | 0.0191 | 4.65E-16 | 0.306 | A | G | -0.0108 | 0.0163 | 0.5065 | 757601 |
| rs882624 | -0.1571 | 0.0185 | 2.33E-17 | 0.6675 | T | C | 0.0069 | 0.0153 | 0.6514 | 757601 |
| rs908951 | -0.1983 | 0.0181 | 7.73E-28 | 0.563 | T | C | 0.0161 | 0.0154 | 0.2956 | 757601 |
| rs917522 | 0.1665 | 0.0273 | 1.04E-09 | 0.115 | T | C | -0.0083 | 0.0219 | 0.7059 | 757601 |
| rs9286351 | -0.1412 | 0.0177 | 1.61E-15 | 0.4188 | A | G | 0.0195 | 0.0146 | 0.1823 | 757601 |
| rs9289557 | -0.119 | 0.0207 | 8.68E-09 | 0.7396 | T | C | 0.0402 | 0.0187 | 0.0317 | 757601 |
| rs9326869 | 0.1096 | 0.02 | 3.99E-08 | 0.7513 | T | C | -0.0168 | 0.0163 | 0.3034 | 757601 |
| rs9365555 | 0.1254 | 0.0187 | 1.96E-11 | 0.3259 | A | G | -0.0062 | 0.0155 | 0.6887 | 757601 |
| rs9399137 | 0.1148 | 0.0197 | 5.83E-09 | 0.2619 | T | C | -0.0034 | 0.0164 | 0.8363 | 757601 |
| rs9406076 | 0.101 | 0.0185 | 4.65E-08 | 0.6722 | T | C | 0.0097 | 0.0154 | 0.5297 | 757601 |
| rs9419374 | 0.1164 | 0.0185 | 3.44E-10 | 0.646 | A | G | 0.0088 | 0.0158 | 0.5784 | 757601 |
| rs9478282 | -0.1994 | 0.0279 | 8.70E-13 | 0.8884 | T | C | -0.0111 | 0.0233 | 0.6324 | 757601 |
| rs9508495 | -0.1944 | 0.0204 | 1.34E-21 | 0.2431 | T | C | 0.0032 | 0.0168 | 0.849 | 757601 |
| rs9526707 | -0.1217 | 0.0186 | 6.59E-11 | 0.6778 | A | G | -0.0128 | 0.0155 | 0.4087 | 757601 |
| rs9563529 | 0.1222 | 0.0215 | 1.38E-08 | 0.7957 | T | G | -0.0022 | 0.0179 | 0.9025 | 757601 |
| rs962369 | 0.1684 | 0.0189 | 6.02E-19 | 0.3013 | T | C | -0.0349 | 0.0158 | 0.02682 | 757601 |
| rs9638084 | 0.1154 | 0.0178 | 8.51E-11 | 0.6022 | A | G | -0.0456 | 0.0149 | 0.002162 | 757601 |
| rs9791312 | -0.1225 | 0.0184 | 2.89E-11 | 0.3452 | A | C | 0.0018 | 0.0152 | 0.9034 | 757601 |
| rs9918907 | -0.1188 | 0.021 | 1.59E-08 | 0.2162 | A | G | 5.00E-04 | 0.0171 | 0.9753 | 757601 |
| rs9932220 | -0.1591 | 0.021 | 3.76E-14 | 0.7823 | A | G | -0.0136 | 0.0179 | 0.4475 | 757601 |
| rs9937801 | 0.1554 | 0.0174 | 4.81E-19 | 0.4308 | T | C | -0.0301 | 0.0145 | 0.03748 | 757601 |
