# Supplementary material for: Genetically determined blood pressure, antihypertensive medications, and risk of Alzheimer’s disease: a Mendelian randomization study
Source: Alzheimers Res Ther. 2021 Feb 9;13:41. doi: 10.1186/s13195-021-00782-y (PMC7874453; doi:10.1186/s13195-021-00782-y)
Supplement: Supplementary file 5 — Additional file 5. Genome-wide significant and independent SNPs that were used as instruments for PP. [file 13195_2021_782_MOESM5_ESM.docx]

**Additional file 5 Genome-wide significant and independent SNPs that were used as instruments for PP**

| SNP | Beta.exposure | SE.exposure | Pval.exposure | EAF | Effect_allele | Other_allele | Beta.outcome | SE.outcome | Pval.outcome | Samplesize |
| --- | --- | --- | --- | --- | --- | --- | --- | --- | --- | --- |
| rs10052777 | -0.2457 | 0.021 | 1.70E-31 | 0.3931 | T | C | -0.0124 | 0.0144 | 0.3898 | 757601 |
| rs10076730 | 0.2217 | 0.0211 | 8.02E-26 | 0.3749 | T | C | 0.0148 | 0.0145 | 0.3061 | 757601 |
| rs10261098 | 0.168 | 0.0285 | 3.95E-09 | 0.8458 | T | C | -0.0224 | 0.0196 | 0.2532 | 757601 |
| rs10305838 | -0.2542 | 0.0293 | 4.65E-18 | 0.1408 | T | C | 0.0329 | 0.0202 | 0.1039 | 757601 |
| rs10433615 | 0.1655 | 0.021 | 3.40E-15 | 0.6065 | T | C | -0.0022 | 0.0147 | 0.8816 | 757601 |
| rs1047922 | -0.2121 | 0.0297 | 9.70E-13 | 0.1516 | T | C | -0.0099 | 0.0226 | 0.6607 | 757601 |
| rs10497529 | -0.4509 | 0.0575 | 4.67E-15 | 0.9648 | A | G | -0.0052 | 0.0463 | 0.9105 | 757601 |
| rs1061651 | 0.1304 | 0.0239 | 4.95E-08 | 0.2648 | T | C | 0.0027 | 0.0171 | 0.8724 | 757601 |
| rs10736585 | 0.1945 | 0.0217 | 2.82E-19 | 0.3355 | T | C | -0.0139 | 0.0149 | 0.3529 | 757601 |
| rs10770612 | 0.3421 | 0.0259 | 5.79E-40 | 0.2025 | A | G | -0.0214 | 0.0185 | 0.2467 | 757601 |
| rs10776752 | 0.3735 | 0.0392 | 1.51E-21 | 0.9199 | T | G | -0.0169 | 0.0284 | 0.5519 | 757601 |
| rs10787515 | -0.1354 | 0.0206 | 4.73E-11 | 0.4785 | T | C | -0.0195 | 0.0144 | 0.174 | 757601 |
| rs10885409 | -0.1879 | 0.0205 | 5.17E-20 | 0.4675 | T | C | 0.0021 | 0.0142 | 0.8832 | 757601 |
| rs10887914 | 0.1461 | 0.0205 | 1.13E-12 | 0.5373 | T | C | -0.0297 | 0.0144 | 0.03884 | 757601 |
| rs10890706 | -0.1622 | 0.0223 | 3.36E-13 | 0.3163 | T | C | 0.0023 | 0.0161 | 0.8879 | 757601 |
| rs10914057 | 0.1155 | 0.0209 | 3.49E-08 | 0.3998 | T | C | -0.018 | 0.0148 | 0.2247 | 757601 |
| rs1091811 | -0.1745 | 0.0275 | 2.28E-10 | 0.8313 | A | G | 0.0295 | 0.0193 | 0.1258 | 757601 |
| rs10941043 | -0.1272 | 0.0225 | 1.65E-08 | 0.29 | T | G | 0.0059 | 0.0157 | 0.7062 | 757601 |
| rs10988442 | 0.1809 | 0.0212 | 1.38E-17 | 0.3801 | A | G | -0.0139 | 0.0147 | 0.3448 | 757601 |
| rs11010470 | 0.1153 | 0.0205 | 1.97E-08 | 0.5006 | T | C | -0.0119 | 0.0143 | 0.4071 | 757601 |
| rs11031051 | -0.172 | 0.0222 | 1.04E-14 | 0.3096 | A | C | 0.0032 | 0.0155 | 0.8338 | 757601 |
| rs11037808 | 0.1366 | 0.0224 | 9.92E-10 | 0.3052 | T | C | 0.0132 | 0.0155 | 0.3934 | 757601 |
| rs11052722 | 0.1127 | 0.0206 | 4.73E-08 | 0.4807 | A | G | 0.0146 | 0.0143 | 0.307 | 757601 |
| rs11065861 | 0.1684 | 0.0249 | 1.37E-11 | 0.2158 | A | G | -0.0377 | 0.0174 | 0.02989 | 757601 |
| rs11100902 | 0.1572 | 0.0207 | 3.21E-14 | 0.5157 | A | G | -0.0105 | 0.0144 | 0.4665 | 757601 |
| rs111478946 | -0.4623 | 0.0276 | 8.22E-63 | 0.8331 | A | G | -5.00E-04 | 0.0196 | 0.9794 | 757601 |
| rs11154027 | 0.1439 | 0.0208 | 4.47E-12 | 0.539 | T | C | -0.0063 | 0.0147 | 0.6666 | 757601 |
| rs11187998 | -0.1403 | 0.0206 | 1.06E-11 | 0.5648 | A | G | 3.00E-04 | 0.0143 | 0.986 | 757601 |
| rs11190709 | 0.337 | 0.0328 | 8.41E-25 | 0.1119 | A | G | 0.0019 | 0.0227 | 0.9319 | 757601 |
| rs112005532 | -0.4967 | 0.0574 | 4.70E-18 | 0.0393 | T | C | 0.0737 | 0.0466 | 0.1137 | 757601 |
| rs1124000 | 0.1237 | 0.022 | 1.92E-08 | 0.6804 | A | G | -0.0039 | 0.0151 | 0.794 | 757601 |
| rs11248862 | 0.2282 | 0.0315 | 4.68E-13 | 0.875 | A | G | -0.0183 | 0.023 | 0.4259 | 757601 |
| rs11257655 | 0.139 | 0.0252 | 3.67E-08 | 0.7904 | T | C | 0.0083 | 0.0175 | 0.6348 | 757601 |
| rs112913898 | -0.5815 | 0.0376 | 5.68E-54 | 0.9179 | A | G | -0.017 | 0.0253 | 0.5018 | 757601 |
| rs1133400 | -0.1609 | 0.0255 | 2.96E-10 | 0.2143 | A | G | -0.0456 | 0.0178 | 0.01049 | 757601 |
| rs114697502 | -0.3813 | 0.0378 | 5.83E-24 | 0.9193 | T | C | 0.0223 | 0.0275 | 0.4167 | 757601 |
| rs11580654 | -0.2087 | 0.035 | 2.56E-09 | 0.9043 | A | G | 0.0243 | 0.0247 | 0.3248 | 757601 |
| rs11603014 | 0.1733 | 0.0258 | 1.89E-11 | 0.8033 | A | G | 0.0149 | 0.018 | 0.4057 | 757601 |
| rs11607056 | -0.1829 | 0.0218 | 4.77E-17 | 0.6728 | T | C | 0.0156 | 0.0153 | 0.3087 | 757601 |
| rs11609905 | -0.1817 | 0.0229 | 2.27E-15 | 0.7252 | T | C | -0.0067 | 0.016 | 0.674 | 757601 |
| rs11629850 | 0.1177 | 0.0205 | 9.65E-09 | 0.4712 | A | G | -0.004 | 0.0144 | 0.7812 | 757601 |
| rs11685352 | -0.1715 | 0.0264 | 8.80E-11 | 0.8162 | A | G | 0.0413 | 0.0183 | 0.02398 | 757601 |
| rs11689667 | 0.2029 | 0.0206 | 7.36E-23 | 0.4556 | T | C | -0.0199 | 0.0142 | 0.1615 | 757601 |
| rs11690961 | 0.3036 | 0.0319 | 1.93E-21 | 0.1167 | A | C | -0.0021 | 0.0223 | 0.9267 | 757601 |
| rs117557920 | 0.2363 | 0.0275 | 8.99E-18 | 0.8057 | A | G | -0.0138 | 0.0207 | 0.5042 | 757601 |
| rs11872627 | -0.2532 | 0.0298 | 1.77E-17 | 0.8611 | T | C | 0.0329 | 0.0207 | 0.1119 | 757601 |
| rs11874246 | 0.1574 | 0.0224 | 1.93E-12 | 0.7043 | T | C | -0.0055 | 0.0157 | 0.7261 | 757601 |
| rs11977526 | -0.4308 | 0.0211 | 1.75E-92 | 0.5982 | A | G | -0.0029 | 0.0149 | 0.8449 | 757601 |
| rs11988241 | -0.1323 | 0.0236 | 2.02E-08 | 0.256 | T | C | -0.0335 | 0.0162 | 0.03942 | 757601 |
| rs1198982 | -0.1242 | 0.0211 | 3.93E-09 | 0.3879 | A | G | -0.0017 | 0.0148 | 0.9095 | 757601 |
| rs12032588 | -0.1294 | 0.0208 | 5.12E-10 | 0.6007 | T | G | 0.0189 | 0.0145 | 0.193 | 757601 |
| rs12045477 | -0.176 | 0.0228 | 1.16E-14 | 0.7119 | T | C | 0.0152 | 0.0158 | 0.3372 | 757601 |
| rs12052878 | -0.1497 | 0.022 | 1.11E-11 | 0.6802 | A | G | 0.0082 | 0.0154 | 0.5943 | 757601 |
| rs12138150 | -0.1954 | 0.0208 | 5.66E-21 | 0.5979 | T | C | 0.0061 | 0.0146 | 0.6772 | 757601 |
| rs12149704 | 0.7476 | 0.0466 | 5.46E-58 | 0.937 | A | G | -0.0234 | 0.0355 | 0.5108 | 757601 |
| rs12153395 | -0.2134 | 0.033 | 1.00E-10 | 0.8855 | A | G | -0.0099 | 0.0244 | 0.6855 | 757601 |
| rs12172847 | -0.1278 | 0.0218 | 4.68E-09 | 0.6775 | A | G | -0.0461 | 0.0152 | 0.002429 | 757601 |
| rs12195276 | -0.1819 | 0.0231 | 3.49E-15 | 0.2748 | T | C | 0.0016 | 0.0164 | 0.9208 | 757601 |
| rs12201429 | 0.3763 | 0.0298 | 1.50E-36 | 0.8618 | T | C | -0.008 | 0.0214 | 0.708 | 757601 |
| rs12216497 | 0.1307 | 0.0206 | 2.25E-10 | 0.4384 | T | C | 0.0096 | 0.0143 | 0.5022 | 757601 |
| rs12264186 | 0.1972 | 0.0263 | 6.90E-14 | 0.8127 | T | C | -0.0055 | 0.0184 | 0.763 | 757601 |
| rs1229984 | -0.5111 | 0.0607 | 3.57E-17 | 0.9607 | T | C | 0.0171 | 0.0351 | 0.6258 | 757601 |
| rs12476956 | 0.1307 | 0.0211 | 6.15E-10 | 0.5485 | T | C | -0.0184 | 0.0151 | 0.2235 | 757601 |
| rs12536419 | -0.6985 | 0.0285 | 6.35E-133 | 0.1581 | A | C | 1.00E-04 | 0.0197 | 0.9973 | 757601 |
| rs12603813 | -0.2683 | 0.0237 | 8.31E-30 | 0.2527 | T | C | -0.0097 | 0.0165 | 0.5578 | 757601 |
| rs12627651 | 0.134 | 0.0232 | 8.16E-09 | 0.7128 | A | G | 0.012 | 0.0166 | 0.4699 | 757601 |
| rs12630450 | 0.1947 | 0.0235 | 1.22E-16 | 0.2671 | A | G | -0.0206 | 0.0163 | 0.2084 | 757601 |
| rs12636123 | 0.1283 | 0.0235 | 4.75E-08 | 0.3446 | T | G | -0.007 | 0.0158 | 0.6563 | 757601 |
| rs12705090 | -0.2361 | 0.0262 | 2.17E-19 | 0.8109 | T | C | 0.0138 | 0.0185 | 0.4556 | 757601 |
| rs1275957 | -0.2082 | 0.0214 | 2.62E-22 | 0.4082 | T | G | -0.0225 | 0.0149 | 0.1314 | 757601 |
| rs13107325 | -0.2527 | 0.0401 | 2.91E-10 | 0.926 | T | C | 0.0202 | 0.0273 | 0.4607 | 757601 |
| rs13149209 | 0.1769 | 0.0249 | 1.24E-12 | 0.2224 | T | C | 0.0065 | 0.0171 | 0.7022 | 757601 |
| rs13189347 | -0.1408 | 0.0206 | 8.13E-12 | 0.448 | A | C | 0.0233 | 0.0144 | 0.1054 | 757601 |
| rs13199674 | 0.2204 | 0.0207 | 1.65E-26 | 0.5571 | A | G | 0.0039 | 0.0144 | 0.7893 | 757601 |
| rs13206305 | -0.156 | 0.0259 | 1.78E-09 | 0.8029 | T | C | -0.0166 | 0.0181 | 0.3603 | 757601 |
| rs13279275 | -0.1501 | 0.0252 | 2.66E-09 | 0.2343 | A | C | 0.0264 | 0.0179 | 0.1396 | 757601 |
| rs13290326 | -0.1562 | 0.0204 | 2.11E-14 | 0.4988 | T | C | -0.0026 | 0.0143 | 0.8562 | 757601 |
| rs13356445 | -0.1415 | 0.025 | 1.45E-08 | 0.7827 | T | C | -0.0157 | 0.0173 | 0.3647 | 757601 |
| rs13409792 | 0.175 | 0.0294 | 2.58E-09 | 0.1404 | A | G | 0.0055 | 0.0207 | 0.7911 | 757601 |
| rs1344652 | -0.3455 | 0.0219 | 3.93E-56 | 0.6834 | A | G | 0.0061 | 0.0153 | 0.6909 | 757601 |
| rs1350100 | 0.1653 | 0.0208 | 1.80E-15 | 0.5549 | A | G | 0.0111 | 0.0147 | 0.451 | 757601 |
| rs1351394 | -0.1811 | 0.0204 | 6.53E-19 | 0.5108 | T | C | -0.0112 | 0.0144 | 0.437 | 757601 |
| rs138285687 | -0.3304 | 0.0517 | 1.68E-10 | 0.9567 | T | C | 0.0207 | 0.0387 | 0.5934 | 757601 |
| rs139919 | -0.2476 | 0.0272 | 8.04E-20 | 0.1803 | T | C | 0.0205 | 0.0198 | 0.3015 | 757601 |
| rs141212865 | 0.1497 | 0.0263 | 1.21E-08 | 0.1944 | A | C | 0.0097 | 0.0183 | 0.5962 | 757601 |
| rs142004400 | 0.3306 | 0.0566 | 5.24E-09 | 0.0361 | A | C | 0.0804 | 0.041 | 0.04966 | 757601 |
| rs142378207 | 0.3473 | 0.0304 | 3.50E-30 | 0.8689 | A | G | -0.0219 | 0.0219 | 0.3178 | 757601 |
| rs143167197 | -0.3069 | 0.0419 | 2.49E-13 | 0.0715 | A | G | -0.0304 | 0.0288 | 0.2907 | 757601 |
| rs144822931 | 0.5092 | 0.0797 | 1.71E-10 | 0.0175 | T | C | 0.0752 | 0.0528 | 0.1545 | 757601 |
| rs1449544 | 0.1927 | 0.0205 | 6.68E-21 | 0.4565 | A | C | -0.0164 | 0.0146 | 0.2624 | 757601 |
| rs1469760 | -0.1891 | 0.0208 | 1.16E-19 | 0.4162 | T | C | 0.0221 | 0.0148 | 0.1362 | 757601 |
| rs150266910 | 0.1731 | 0.0267 | 9.57E-11 | 0.8232 | T | C | 0.0192 | 0.0185 | 0.2983 | 757601 |
| rs1520222 | 0.1285 | 0.0232 | 2.89E-08 | 0.2626 | A | G | 0.0162 | 0.0162 | 0.3171 | 757601 |
| rs1563788 | 0.2119 | 0.0226 | 5.81E-21 | 0.7134 | T | C | 1.00E-04 | 0.0155 | 0.9957 | 757601 |
| rs1599116 | -0.1682 | 0.0292 | 8.66E-09 | 0.1455 | T | G | -0.0208 | 0.0201 | 0.3011 | 757601 |
| rs1644318 | -0.2308 | 0.021 | 3.92E-28 | 0.3866 | T | C | -0.017 | 0.0145 | 0.2401 | 757601 |
| rs1687692 | 0.1594 | 0.026 | 8.57E-10 | 0.7998 | A | G | 0.0229 | 0.0183 | 0.2104 | 757601 |
| rs16939351 | -0.29 | 0.0437 | 3.34E-11 | 0.9406 | A | G | 0.0446 | 0.0314 | 0.1559 | 757601 |
| rs17010957 | -0.3456 | 0.0292 | 2.30E-32 | 0.1461 | T | C | -0.0099 | 0.0208 | 0.6349 | 757601 |
| rs17037452 | 0.3833 | 0.0278 | 2.83E-43 | 0.1608 | A | G | -0.0576 | 0.0196 | 0.003297 | 757601 |
| rs17171688 | -0.3916 | 0.0509 | 1.50E-14 | 0.9541 | A | G | 0.0946 | 0.0356 | 0.007924 | 757601 |
| rs17248720 | -0.2289 | 0.0326 | 2.08E-12 | 0.8824 | T | C | -0.0066 | 0.0223 | 0.7684 | 757601 |
| rs17271730 | 0.1631 | 0.0213 | 2.13E-14 | 0.3628 | A | G | -0.0023 | 0.0149 | 0.8762 | 757601 |
| rs17535443 | -0.3649 | 0.023 | 7.60E-57 | 0.727 | A | G | 0.0018 | 0.0164 | 0.9124 | 757601 |
| rs17562391 | 0.1713 | 0.0209 | 2.32E-16 | 0.5818 | T | C | -0.004 | 0.0146 | 0.7837 | 757601 |
| rs17608766 | -0.5274 | 0.0295 | 2.12E-71 | 0.1443 | T | C | 0.0216 | 0.0213 | 0.3112 | 757601 |
| rs17732513 | -0.1465 | 0.0216 | 1.10E-11 | 0.649 | T | C | -5.00E-04 | 0.0151 | 0.9712 | 757601 |
| rs1779240 | -0.2018 | 0.0241 | 5.21E-17 | 0.2357 | A | G | 0.0154 | 0.0167 | 0.3579 | 757601 |
| rs1800470 | -0.15 | 0.0213 | 1.76E-12 | 0.3766 | A | G | 0.0085 | 0.0148 | 0.5663 | 757601 |
| rs1834596 | -0.1325 | 0.0216 | 9.37E-10 | 0.6538 | T | C | -0.0123 | 0.015 | 0.4124 | 757601 |
| rs1850507 | 0.168 | 0.026 | 1.10E-10 | 0.1966 | T | G | 0.0103 | 0.0185 | 0.5767 | 757601 |
| rs1866628 | 0.1201 | 0.0205 | 4.82E-09 | 0.5232 | T | C | -0.014 | 0.0143 | 0.3309 | 757601 |
| rs1918973 | 0.1253 | 0.0205 | 9.91E-10 | 0.5416 | A | G | 0.0208 | 0.0143 | 0.1464 | 757601 |
| rs1965942 | 0.1276 | 0.0217 | 3.78E-09 | 0.4614 | A | G | 0.0199 | 0.0158 | 0.2089 | 757601 |
| rs1997571 | -0.1448 | 0.0208 | 3.46E-12 | 0.409 | A | G | -0.0046 | 0.0144 | 0.7503 | 757601 |
| rs200528 | -0.2295 | 0.0258 | 6.38E-19 | 0.8069 | A | G | 0.0239 | 0.0178 | 0.181 | 757601 |
| rs2015637 | 0.5012 | 0.0345 | 8.89E-48 | 0.0996 | T | C | 8.00E-04 | 0.0235 | 0.9726 | 757601 |
| rs2055120 | -0.5578 | 0.0722 | 1.11E-14 | 0.0223 | A | G | -0.0842 | 0.0536 | 0.1159 | 757601 |
| rs2102397 | 0.1616 | 0.021 | 1.58E-14 | 0.4936 | A | C | -0.0427 | 0.0154 | 0.005625 | 757601 |
| rs2107595 | 0.4435 | 0.0282 | 8.24E-56 | 0.8414 | A | G | 4.00E-04 | 0.0194 | 0.9843 | 757601 |
| rs2109019 | -0.2464 | 0.0256 | 5.20E-22 | 0.7882 | A | C | -0.0118 | 0.018 | 0.514 | 757601 |
| rs2143618 | -0.2211 | 0.0277 | 1.41E-15 | 0.1638 | A | G | 0.0021 | 0.0195 | 0.9142 | 757601 |
| rs2148306 | -0.1802 | 0.0207 | 2.78E-18 | 0.4227 | A | C | 0.0208 | 0.0144 | 0.1471 | 757601 |
| rs2175337 | 0.1656 | 0.021 | 3.52E-15 | 0.3892 | A | C | -0.0167 | 0.0147 | 0.2555 | 757601 |
| rs2206815 | -0.3609 | 0.0207 | 4.27E-68 | 0.5022 | A | C | 5.00E-04 | 0.0144 | 0.972 | 757601 |
| rs2215590 | 0.1732 | 0.0235 | 1.67E-13 | 0.7451 | T | C | -2.00E-04 | 0.0161 | 0.991 | 757601 |
| rs222837 | 0.1265 | 0.0209 | 1.34E-09 | 0.4886 | T | C | -0.0071 | 0.0144 | 0.6208 | 757601 |
| rs2242652 | 0.1577 | 0.0281 | 1.93E-08 | 0.8049 | A | G | -0.0261 | 0.0235 | 0.2668 | 757601 |
| rs2255055 | -0.1259 | 0.0211 | 2.32E-09 | 0.6184 | T | C | 0.0112 | 0.015 | 0.4551 | 757601 |
| rs2288277 | -0.2645 | 0.036 | 2.16E-13 | 0.9096 | T | C | 0.0384 | 0.0254 | 0.1298 | 757601 |
| rs2289125 | -0.3847 | 0.0255 | 1.82E-51 | 0.7798 | A | C | 0.0053 | 0.018 | 0.7678 | 757601 |
| rs2328473 | -0.126 | 0.0209 | 1.71E-09 | 0.5983 | A | G | 0.0035 | 0.0146 | 0.8108 | 757601 |
| rs2344402 | 0.1496 | 0.0214 | 2.62E-12 | 0.4038 | T | C | 0.0027 | 0.015 | 0.8552 | 757601 |
| rs2354862 | 0.1272 | 0.0215 | 3.11E-09 | 0.3597 | A | C | 0.0143 | 0.0148 | 0.3351 | 757601 |
| rs2358891 | 0.1593 | 0.0241 | 4.08E-11 | 0.7545 | A | G | 0.0092 | 0.0168 | 0.5855 | 757601 |
| rs2395655 | 0.1566 | 0.0211 | 1.08E-13 | 0.3882 | A | G | -0.0082 | 0.0149 | 0.5833 | 757601 |
| rs2493134 | -0.1344 | 0.0209 | 1.35E-10 | 0.4068 | T | C | 0.0312 | 0.0144 | 0.03046 | 757601 |
| rs2493296 | 0.1894 | 0.03 | 2.64E-10 | 0.8576 | T | C | -0.0254 | 0.0206 | 0.2172 | 757601 |
| rs2498323 | 0.2957 | 0.035 | 3.07E-17 | 0.9018 | A | G | -0.0493 | 0.0239 | 0.03945 | 757601 |
| rs251252 | 0.1249 | 0.022 | 1.35E-08 | 0.6694 | T | C | -0.0133 | 0.0156 | 0.3939 | 757601 |
| rs2540951 | 0.2243 | 0.021 | 1.26E-26 | 0.3787 | A | G | 0.0178 | 0.0147 | 0.2242 | 757601 |
| rs256824 | -0.1408 | 0.0232 | 1.36E-09 | 0.7293 | T | C | -0.0231 | 0.0162 | 0.1537 | 757601 |
| rs2610990 | -0.1586 | 0.0233 | 1.06E-11 | 0.7364 | A | G | -0.0231 | 0.0164 | 0.1587 | 757601 |
| rs263017 | 0.1356 | 0.0204 | 3.23E-11 | 0.5047 | A | G | 0.0279 | 0.0143 | 0.05034 | 757601 |
| rs263532 | 0.1186 | 0.0208 | 1.25E-08 | 0.4244 | T | C | 6.00E-04 | 0.0146 | 0.9657 | 757601 |
| rs2820443 | 0.1857 | 0.0224 | 1.30E-16 | 0.2911 | T | C | -0.0334 | 0.0159 | 0.03511 | 757601 |
| rs2834440 | 0.1174 | 0.021 | 2.27E-08 | 0.3796 | A | G | 0.0206 | 0.0146 | 0.1579 | 757601 |
| rs28499085 | 0.1569 | 0.023 | 9.35E-12 | 0.2746 | A | G | -0.0278 | 0.0162 | 0.08625 | 757601 |
| rs28572357 | -0.1504 | 0.0209 | 6.83E-13 | 0.3971 | A | C | 0.0263 | 0.0148 | 0.07574 | 757601 |
| rs28651151 | 0.1448 | 0.0231 | 3.59E-10 | 0.2752 | T | G | -0.0024 | 0.0161 | 0.8799 | 757601 |
| rs2953930 | 0.1712 | 0.0304 | 1.76E-08 | 0.8676 | T | C | 0.0245 | 0.0216 | 0.2571 | 757601 |
| rs2969036 | 0.1308 | 0.023 | 1.36E-08 | 0.693 | T | G | 0.0073 | 0.0165 | 0.6593 | 757601 |
| rs2978456 | -0.1781 | 0.0212 | 5.14E-17 | 0.4487 | T | C | 0.0341 | 0.0148 | 0.02157 | 757601 |
| rs2983896 | 0.2127 | 0.0249 | 1.26E-17 | 0.7815 | A | G | -0.022 | 0.0172 | 0.1996 | 757601 |
| rs3006576 | 0.1923 | 0.0224 | 9.05E-18 | 0.296 | T | C | -0.0187 | 0.0155 | 0.227 | 757601 |
| rs30232 | -0.1234 | 0.0209 | 3.37E-09 | 0.4175 | A | G | -0.0237 | 0.0148 | 0.1092 | 757601 |
| rs307359 | -0.3344 | 0.0442 | 3.92E-14 | 0.9312 | A | G | -0.0529 | 0.0355 | 0.137 | 757601 |
| rs3098186 | -0.1735 | 0.0207 | 4.40E-17 | 0.4844 | T | C | -0.045 | 0.0146 | 0.001971 | 757601 |
| rs3134950 | 0.2928 | 0.0222 | 8.16E-40 | 0.3758 | A | C | -0.0444 | 0.0153 | 0.003702 | 757601 |
| rs324075 | 0.2009 | 0.0276 | 3.52E-13 | 0.1869 | A | G | 0.041 | 0.0204 | 0.04443 | 757601 |
| rs34587622 | -0.2084 | 0.035 | 2.49E-09 | 0.8903 | T | C | -0.0142 | 0.027 | 0.5978 | 757601 |
| rs34587684 | 0.1448 | 0.0254 | 1.15E-08 | 0.7958 | T | C | 0.0087 | 0.0179 | 0.6278 | 757601 |
| rs35429 | 0.1777 | 0.0212 | 4.94E-17 | 0.3869 | A | G | 0.0099 | 0.015 | 0.5111 | 757601 |
| rs35680304 | 0.1848 | 0.021 | 1.60E-18 | 0.407 | T | C | 0.0059 | 0.0148 | 0.6903 | 757601 |
| rs36047283 | 0.3743 | 0.0334 | 3.42E-29 | 0.1235 | A | G | 0.0154 | 0.0239 | 0.5191 | 757601 |
| rs365990 | 0.3079 | 0.0213 | 1.75E-47 | 0.3663 | A | G | 0.0058 | 0.015 | 0.6958 | 757601 |
| rs37060 | 0.1489 | 0.0237 | 3.12E-10 | 0.7535 | A | G | -0.0047 | 0.0165 | 0.7773 | 757601 |
| rs3742182 | -0.1863 | 0.0261 | 9.00E-13 | 0.1899 | T | C | 0.008 | 0.0186 | 0.6669 | 757601 |
| rs3753802 | 0.1161 | 0.0209 | 2.71E-08 | 0.3972 | T | C | -0.0062 | 0.0146 | 0.6721 | 757601 |
| rs3760994 | -0.144 | 0.0218 | 4.27E-11 | 0.5084 | A | G | -0.0031 | 0.0165 | 0.8532 | 757601 |
| rs3780190 | -0.1406 | 0.0208 | 1.35E-11 | 0.5365 | A | G | -0.0096 | 0.0154 | 0.5344 | 757601 |
| rs3819532 | -0.1337 | 0.0209 | 1.45E-10 | 0.6088 | T | C | 0.0014 | 0.0146 | 0.9234 | 757601 |
| rs385437 | 0.1626 | 0.0297 | 4.51E-08 | 0.1397 | A | G | 0.007 | 0.0204 | 0.7326 | 757601 |
| rs3915425 | 0.1913 | 0.022 | 4.00E-18 | 0.3182 | T | C | 0.005 | 0.0156 | 0.7495 | 757601 |
| rs4075289 | 0.1539 | 0.0233 | 4.12E-11 | 0.293 | T | G | 0.0107 | 0.0166 | 0.518 | 757601 |
| rs4076789 | -0.1281 | 0.0231 | 2.93E-08 | 0.7341 | A | G | -0.0262 | 0.0163 | 0.1067 | 757601 |
| rs42377 | -0.3175 | 0.0225 | 2.42E-45 | 0.6956 | A | G | -0.0534 | 0.0154 | 0.0005432 | 757601 |
| rs4245599 | -0.1548 | 0.0207 | 7.70E-14 | 0.5421 | A | G | 0.0285 | 0.0143 | 0.04621 | 757601 |
| rs4304924 | -0.1198 | 0.0208 | 8.67E-09 | 0.4323 | A | G | 0.0276 | 0.0146 | 0.0592 | 757601 |
| rs4347920 | -0.1263 | 0.0211 | 2.23E-09 | 0.5966 | A | C | -0.0222 | 0.0151 | 0.1418 | 757601 |
| rs4440615 | -0.2492 | 0.0212 | 8.22E-32 | 0.368 | A | G | 0.0204 | 0.0149 | 0.1724 | 757601 |
| rs4441458 | -0.1289 | 0.0227 | 1.42E-08 | 0.7171 | T | C | 0.0127 | 0.0158 | 0.4238 | 757601 |
| rs4491476 | -0.1678 | 0.0211 | 1.89E-15 | 0.5903 | A | G | -0.0024 | 0.0147 | 0.8708 | 757601 |
| rs4551303 | -0.1873 | 0.0221 | 2.06E-17 | 0.6835 | T | C | -0.0367 | 0.0158 | 0.01993 | 757601 |
| rs4553000 | -0.1464 | 0.0204 | 7.47E-13 | 0.4861 | T | C | -0.0182 | 0.0142 | 0.2006 | 757601 |
| rs4559481 | -0.1174 | 0.021 | 2.30E-08 | 0.5181 | A | G | -0.0239 | 0.015 | 0.1102 | 757601 |
| rs4594944 | -0.1256 | 0.0221 | 1.38E-08 | 0.3159 | A | G | 0.0067 | 0.0154 | 0.6631 | 757601 |
| rs4664080 | -0.135 | 0.0209 | 1.03E-10 | 0.6022 | A | G | 0.0077 | 0.0146 | 0.5985 | 757601 |
| rs4672081 | -0.142 | 0.0206 | 4.98E-12 | 0.435 | T | C | -0.0049 | 0.0144 | 0.7323 | 757601 |
| rs4674114 | -0.2116 | 0.0256 | 1.28E-16 | 0.799 | A | G | -0.0083 | 0.0177 | 0.6403 | 757601 |
| rs4691670 | -0.2359 | 0.0205 | 1.11E-30 | 0.4671 | T | C | 0.0345 | 0.0142 | 0.01553 | 757601 |
| rs4796514 | -0.2313 | 0.021 | 4.11E-28 | 0.3914 | T | C | -0.0155 | 0.0147 | 0.2902 | 757601 |
| rs4819852 | 0.2486 | 0.0228 | 8.73E-28 | 0.7129 | A | G | -0.0276 | 0.0159 | 0.08176 | 757601 |
| rs4842266 | -0.1675 | 0.0222 | 4.95E-14 | 0.3138 | A | G | -0.015 | 0.0153 | 0.327 | 757601 |
| rs486098 | 0.1536 | 0.0232 | 3.68E-11 | 0.7309 | T | C | -0.0091 | 0.0164 | 0.5785 | 757601 |
| rs4873492 | 0.2202 | 0.0273 | 8.05E-16 | 0.8277 | T | C | 0.0199 | 0.0185 | 0.2812 | 757601 |
| rs4922591 | -0.1513 | 0.0214 | 1.39E-12 | 0.6133 | T | C | -0.0039 | 0.0149 | 0.7951 | 757601 |
| rs4946265 | 0.1546 | 0.0205 | 5.38E-14 | 0.488 | A | G | -0.0016 | 0.0143 | 0.911 | 757601 |
| rs4950838 | 0.212 | 0.0346 | 8.60E-10 | 0.0985 | T | C | -0.0055 | 0.0234 | 0.815 | 757601 |
| rs4952955 | -0.1424 | 0.0258 | 3.48E-08 | 0.7983 | T | C | 0.0216 | 0.0181 | 0.2329 | 757601 |
| rs4968716 | 0.1364 | 0.0211 | 9.84E-11 | 0.4798 | T | C | 0.0037 | 0.0153 | 0.8081 | 757601 |
| rs4977492 | -0.1252 | 0.0216 | 6.70E-09 | 0.3379 | T | C | -0.0187 | 0.0159 | 0.24 | 757601 |
| rs4980515 | 0.1628 | 0.0205 | 2.29E-15 | 0.5012 | T | C | -0.0405 | 0.0143 | 0.004616 | 757601 |
| rs558248 | 0.1915 | 0.0212 | 1.61E-19 | 0.378 | A | G | -0.0176 | 0.0148 | 0.2331 | 757601 |
| rs55947600 | -0.1895 | 0.0206 | 4.10E-20 | 0.5377 | A | G | 0.0205 | 0.0143 | 0.1525 | 757601 |
| rs55962736 | -0.1562 | 0.0205 | 2.49E-14 | 0.4906 | T | G | -0.002 | 0.0143 | 0.8901 | 757601 |
| rs560887 | -0.1904 | 0.0223 | 1.57E-17 | 0.7011 | T | C | -0.0256 | 0.0157 | 0.1031 | 757601 |
| rs56090516 | -0.1304 | 0.0216 | 1.56E-09 | 0.3377 | T | C | -0.0108 | 0.015 | 0.4721 | 757601 |
| rs56255660 | 0.2339 | 0.0236 | 4.23E-23 | 0.7422 | A | C | 0.0255 | 0.0167 | 0.1275 | 757601 |
| rs56287081 | 0.2018 | 0.0262 | 1.40E-14 | 0.811 | A | G | -0.0104 | 0.0183 | 0.5682 | 757601 |
| rs56288724 | -0.2377 | 0.0211 | 1.99E-29 | 0.4174 | A | G | -0.0161 | 0.0149 | 0.278 | 757601 |
| rs57139556 | 0.3087 | 0.0399 | 1.02E-14 | 0.0714 | A | G | -0.0082 | 0.0285 | 0.7728 | 757601 |
| rs573455 | 0.2515 | 0.0206 | 2.37E-34 | 0.5386 | A | G | 0.0108 | 0.0145 | 0.4578 | 757601 |
| rs5753103 | 0.1377 | 0.0206 | 2.62E-11 | 0.549 | A | G | -0.0028 | 0.0143 | 0.8443 | 757601 |
| rs57946343 | 0.2405 | 0.0289 | 8.47E-17 | 0.1475 | T | C | 0.0043 | 0.0199 | 0.8308 | 757601 |
| rs58232567 | -0.32 | 0.0523 | 9.62E-10 | 0.9561 | A | G | -0.0122 | 0.0337 | 0.7174 | 757601 |
| rs58278271 | -0.2276 | 0.0368 | 6.39E-10 | 0.9154 | A | G | 0.0176 | 0.0256 | 0.4911 | 757601 |
| rs6006987 | 0.1312 | 0.0229 | 1.08E-08 | 0.7231 | A | C | -0.0097 | 0.0157 | 0.5373 | 757601 |
| rs60255247 | 0.2491 | 0.033 | 4.53E-14 | 0.1125 | A | C | 0.0136 | 0.0239 | 0.5705 | 757601 |
| rs6031431 | -0.1575 | 0.0207 | 2.72E-14 | 0.4625 | A | G | -0.0124 | 0.0146 | 0.3925 | 757601 |
| rs604723 | -0.2689 | 0.023 | 1.46E-31 | 0.7245 | T | C | -0.001 | 0.0158 | 0.9477 | 757601 |
| rs60672471 | 0.1919 | 0.0333 | 7.96E-09 | 0.1071 | T | C | 0.0154 | 0.0232 | 0.5076 | 757601 |
| rs6078000 | -0.1946 | 0.0226 | 8.02E-18 | 0.2844 | A | G | 0.0196 | 0.0157 | 0.2143 | 757601 |
| rs60991988 | 0.5294 | 0.0337 | 1.44E-55 | 0.1069 | T | G | 0.0083 | 0.0228 | 0.7157 | 757601 |
| rs62055086 | 0.1854 | 0.0243 | 2.55E-14 | 0.7078 | T | C | -0.0092 | 0.0187 | 0.6222 | 757601 |
| rs62062581 | 0.1903 | 0.0282 | 1.56E-11 | 0.1684 | T | G | -0.0298 | 0.0205 | 0.1458 | 757601 |
| rs62111832 | 0.2373 | 0.0414 | 9.78E-09 | 0.9336 | A | G | -0.0511 | 0.0292 | 0.08001 | 757601 |
| rs62270945 | 0.5276 | 0.0651 | 5.17E-16 | 0.9711 | T | C | 0.014 | 0.046 | 0.7609 | 757601 |
| rs62278541 | 0.1677 | 0.0214 | 4.84E-15 | 0.3526 | A | G | 0.0143 | 0.0149 | 0.3385 | 757601 |
| rs62449490 | 0.1137 | 0.0207 | 3.72E-08 | 0.4588 | T | G | -0.0028 | 0.0144 | 0.8489 | 757601 |
| rs629445 | -0.1319 | 0.021 | 3.53E-10 | 0.6106 | A | G | 0.0102 | 0.0149 | 0.4932 | 757601 |
| rs631441 | -0.1543 | 0.0222 | 3.56E-12 | 0.3053 | T | G | -0.0297 | 0.0156 | 0.0566 | 757601 |
| rs6415872 | 0.1215 | 0.0206 | 3.70E-09 | 0.5087 | A | G | 0.0035 | 0.0144 | 0.8094 | 757601 |
| rs6461992 | -0.4361 | 0.04 | 1.03E-27 | 0.9261 | A | G | -0.0052 | 0.0282 | 0.8527 | 757601 |
| rs6504252 | -0.2951 | 0.0504 | 4.70E-09 | 0.9481 | T | C | -0.0241 | 0.0368 | 0.5131 | 757601 |
| rs6544652 | -0.1441 | 0.024 | 1.95E-09 | 0.7642 | T | C | 0.0107 | 0.0167 | 0.5214 | 757601 |
| rs6598886 | 0.218 | 0.0367 | 2.97E-09 | 0.088 | T | C | 0.0152 | 0.0245 | 0.5363 | 757601 |
| rs6601523 | -0.205 | 0.0208 | 7.91E-23 | 0.4099 | A | G | 0.0064 | 0.0147 | 0.661 | 757601 |
| rs663640 | -0.1547 | 0.025 | 5.94E-10 | 0.783 | T | C | -0.0362 | 0.0176 | 0.03949 | 757601 |
| rs6731373 | 0.1336 | 0.0221 | 1.43E-09 | 0.6503 | A | G | 6.00E-04 | 0.0162 | 0.9704 | 757601 |
| rs6747874 | 0.1704 | 0.0247 | 5.02E-12 | 0.7775 | A | G | 0.0209 | 0.0173 | 0.2271 | 757601 |
| rs6766170 | -0.165 | 0.0207 | 1.36E-15 | 0.5067 | A | C | -0.0045 | 0.0144 | 0.7536 | 757601 |
| rs67772913 | 0.2307 | 0.0225 | 1.14E-24 | 0.3041 | A | G | 0.0046 | 0.016 | 0.7728 | 757601 |
| rs6788984 | 0.1882 | 0.0293 | 1.31E-10 | 0.144 | A | G | -0.0116 | 0.0195 | 0.5509 | 757601 |
| rs6806529 | 0.1372 | 0.0209 | 5.81E-11 | 0.5662 | A | C | 0.0102 | 0.0146 | 0.4882 | 757601 |
| rs68100343 | 0.1425 | 0.0235 | 1.28E-09 | 0.7352 | T | C | 0.0067 | 0.017 | 0.6912 | 757601 |
| rs6823199 | 0.1567 | 0.0236 | 3.07E-11 | 0.2565 | T | C | -0.02 | 0.0163 | 0.2191 | 757601 |
| rs686722 | 0.3174 | 0.022 | 4.20E-47 | 0.6381 | T | C | -0.0222 | 0.0154 | 0.1495 | 757601 |
| rs6920534 | 0.2738 | 0.0357 | 1.62E-14 | 0.0973 | T | C | -0.0205 | 0.0248 | 0.4083 | 757601 |
| rs6951894 | -0.1416 | 0.0208 | 8.85E-12 | 0.5757 | A | G | 0.0126 | 0.0144 | 0.3822 | 757601 |
| rs696 | 0.2107 | 0.0214 | 7.48E-23 | 0.6317 | T | C | 0.0215 | 0.0149 | 0.1485 | 757601 |
| rs7011889 | 0.1167 | 0.0207 | 1.64E-08 | 0.4454 | A | C | -0.0171 | 0.0143 | 0.2308 | 757601 |
| rs702395 | 0.1437 | 0.0207 | 4.17E-12 | 0.5634 | T | C | 0.0408 | 0.0146 | 0.005262 | 757601 |
| rs704191 | 0.1628 | 0.0206 | 2.74E-15 | 0.5369 | T | C | -0.0184 | 0.0145 | 0.2037 | 757601 |
| rs7058 | 0.1804 | 0.0206 | 1.86E-18 | 0.537 | T | G | -0.0148 | 0.0143 | 0.3018 | 757601 |
| rs7070115 | -0.2565 | 0.0208 | 4.63E-35 | 0.4304 | A | G | 0.0235 | 0.0146 | 0.1068 | 757601 |
| rs7095472 | 0.1156 | 0.0211 | 4.12E-08 | 0.5308 | A | G | 0.0276 | 0.0149 | 0.06443 | 757601 |
| rs7099368 | 0.1417 | 0.0209 | 1.16E-11 | 0.41 | T | C | -0.0285 | 0.0145 | 0.0496 | 757601 |
| rs7107356 | -0.234 | 0.0205 | 3.24E-30 | 0.5044 | A | G | -0.0668 | 0.0143 | 2.79E-06 | 757601 |
| rs7119612 | -0.246 | 0.0354 | 3.64E-12 | 0.9059 | T | C | 0.0431 | 0.025 | 0.08398 | 757601 |
| rs714417 | -0.2229 | 0.0224 | 2.53E-23 | 0.7001 | T | C | 0.0242 | 0.0154 | 0.1153 | 757601 |
| rs71594307 | 0.2664 | 0.0488 | 4.83E-08 | 0.9513 | A | G | 0.0414 | 0.037 | 0.2631 | 757601 |
| rs7178506 | -0.1229 | 0.0216 | 1.30E-08 | 0.3882 | T | C | 0.0113 | 0.0156 | 0.4671 | 757601 |
| rs7214 | 0.1255 | 0.0207 | 1.32E-09 | 0.4313 | T | G | 0.0424 | 0.0145 | 0.003437 | 757601 |
| rs7236548 | 0.3621 | 0.0264 | 8.48E-43 | 0.8152 | A | C | -0.0046 | 0.0183 | 0.8013 | 757601 |
| rs7245814 | -0.2909 | 0.0345 | 3.75E-17 | 0.9031 | A | G | 0.0209 | 0.0238 | 0.3796 | 757601 |
| rs72664332 | 0.2758 | 0.0354 | 6.81E-15 | 0.0922 | A | C | -0.0103 | 0.0248 | 0.6778 | 757601 |
| rs72676189 | -0.204 | 0.0294 | 3.62E-12 | 0.1405 | A | G | 0.0091 | 0.0203 | 0.6533 | 757601 |
| rs72761109 | 0.1583 | 0.0223 | 1.19E-12 | 0.6971 | T | C | 0.0222 | 0.0158 | 0.1603 | 757601 |
| rs72830615 | 0.1794 | 0.0211 | 1.59E-17 | 0.4017 | A | G | -0.0171 | 0.0146 | 0.2413 | 757601 |
| rs72874178 | -0.3788 | 0.0242 | 4.29E-55 | 0.7655 | A | G | -0.0031 | 0.0171 | 0.8567 | 757601 |
| rs72884380 | -0.1245 | 0.021 | 3.11E-09 | 0.3793 | T | C | -0.0079 | 0.0148 | 0.596 | 757601 |
| rs72943207 | 0.1439 | 0.0258 | 2.33E-08 | 0.7981 | A | G | -0.0133 | 0.0187 | 0.4759 | 757601 |
| rs7313556 | -0.1396 | 0.0214 | 6.79E-11 | 0.652 | A | G | 0.0093 | 0.0148 | 0.528 | 757601 |
| rs73158180 | -0.1693 | 0.023 | 1.76E-13 | 0.2966 | A | C | 9.00E-04 | 0.0162 | 0.9562 | 757601 |
| rs7321688 | 0.1888 | 0.0242 | 6.48E-15 | 0.7672 | A | C | 0.0049 | 0.0168 | 0.7724 | 757601 |
| rs7338758 | 0.1575 | 0.024 | 5.49E-11 | 0.7561 | T | C | -8.00E-04 | 0.0168 | 0.9623 | 757601 |
| rs7341594 | 0.5069 | 0.0248 | 5.56E-93 | 0.7797 | A | G | 0.0364 | 0.0171 | 0.03363 | 757601 |
| rs73727606 | 0.2532 | 0.0411 | 6.99E-10 | 0.9286 | A | G | 0.0161 | 0.0307 | 0.5995 | 757601 |
| rs73767089 | 0.2843 | 0.0332 | 1.08E-17 | 0.1082 | T | C | 0 | 0.0231 | 0.9999 | 757601 |
| rs73900405 | 0.1651 | 0.0237 | 3.34E-12 | 0.2482 | A | G | -0.0096 | 0.0163 | 0.5568 | 757601 |
| rs74048200 | -0.2268 | 0.039 | 6.05E-09 | 0.0861 | A | G | -0.0116 | 0.0288 | 0.6873 | 757601 |
| rs7412 | -0.3769 | 0.0391 | 5.73E-22 | 0.9182 | T | C | -0.4673 | 0.0305 | 6.40E-53 | 757601 |
| rs74179970 | 0.272 | 0.0387 | 2.09E-12 | 0.9188 | A | G | 0.0052 | 0.0287 | 0.8575 | 757601 |
| rs74889068 | 0.2053 | 0.0298 | 5.48E-12 | 0.8547 | A | G | 0.0395 | 0.0204 | 0.0536 | 757601 |
| rs7491248 | 0.1544 | 0.0247 | 3.94E-10 | 0.776 | A | G | -0.0103 | 0.017 | 0.5429 | 757601 |
| rs7497304 | 0.2821 | 0.0223 | 1.39E-36 | 0.6731 | T | G | 0.0044 | 0.0162 | 0.7845 | 757601 |
| rs7500448 | 0.3589 | 0.0239 | 3.62E-51 | 0.2536 | A | G | -0.0147 | 0.0168 | 0.3831 | 757601 |
| rs75016974 | -0.219 | 0.0299 | 2.50E-13 | 0.858 | T | C | -0.012 | 0.0215 | 0.5751 | 757601 |
| rs753361 | 0.1289 | 0.0215 | 2.03E-09 | 0.5789 | A | G | 0.0106 | 0.0152 | 0.4852 | 757601 |
| rs75461554 | -0.1959 | 0.0256 | 1.84E-14 | 0.7993 | T | C | 0.0092 | 0.0176 | 0.6002 | 757601 |
| rs75758489 | -0.1763 | 0.0291 | 1.36E-09 | 0.1777 | T | C | 0.0063 | 0.0226 | 0.7808 | 757601 |
| rs7578166 | 0.1383 | 0.0209 | 4.06E-11 | 0.6139 | A | C | 0.0306 | 0.0146 | 0.03622 | 757601 |
| rs75887402 | 0.5292 | 0.065 | 4.05E-16 | 0.029 | T | C | -0.1053 | 0.0455 | 0.02056 | 757601 |
| rs7603849 | -0.1564 | 0.0205 | 2.28E-14 | 0.522 | A | C | 0.0116 | 0.0142 | 0.414 | 757601 |
| rs76183925 | -0.2019 | 0.0331 | 1.12E-09 | 0.1103 | T | C | 0.0316 | 0.0234 | 0.1771 | 757601 |
| rs7630745 | 0.1636 | 0.0215 | 2.73E-14 | 0.3409 | T | C | -0.0036 | 0.015 | 0.812 | 757601 |
| rs7707563 | 0.1545 | 0.0238 | 8.84E-11 | 0.7555 | T | C | -0.0255 | 0.0167 | 0.1278 | 757601 |
| rs77301788 | -0.1633 | 0.0221 | 1.67E-13 | 0.3094 | T | C | 0.0159 | 0.0155 | 0.3052 | 757601 |
| rs7733331 | -0.3378 | 0.0209 | 6.01E-59 | 0.6008 | T | C | 0.0225 | 0.0145 | 0.1221 | 757601 |
| rs7763294 | -0.1551 | 0.022 | 1.87E-12 | 0.6838 | T | G | -0.0099 | 0.0153 | 0.5168 | 757601 |
| rs7774311 | -0.3547 | 0.0288 | 9.11E-35 | 0.1511 | A | G | 0.0078 | 0.0204 | 0.7 | 757601 |
| rs7821832 | 0.2137 | 0.0236 | 1.54E-19 | 0.2552 | T | G | 0.0135 | 0.0163 | 0.4076 | 757601 |
| rs78378222 | 1.0488 | 0.0945 | 1.28E-28 | 0.0139 | T | G | 0.04 | 0.0735 | 0.5864 | 757601 |
| rs7853859 | 0.1212 | 0.0212 | 1.11E-08 | 0.3671 | T | C | 0.0241 | 0.0147 | 0.1007 | 757601 |
| rs7854147 | 0.2778 | 0.0314 | 8.16E-19 | 0.1227 | A | G | 0.0019 | 0.0227 | 0.932 | 757601 |
| rs7857437 | 0.3696 | 0.0591 | 4.13E-10 | 0.9682 | T | C | 0.0535 | 0.0392 | 0.1725 | 757601 |
| rs786923 | -0.1984 | 0.021 | 3.98E-21 | 0.3764 | T | C | 0.0031 | 0.0146 | 0.8332 | 757601 |
| rs78799967 | -0.4695 | 0.0689 | 9.39E-12 | 0.9735 | T | C | 0.0777 | 0.0494 | 0.1156 | 757601 |
| rs78806058 | -0.1873 | 0.0312 | 1.95E-09 | 0.8644 | A | G | 0.0453 | 0.0236 | 0.05511 | 757601 |
| rs79409628 | -0.3086 | 0.0368 | 5.24E-17 | 0.9154 | T | G | 0.0076 | 0.0253 | 0.7644 | 757601 |
| rs7977311 | -0.199 | 0.032 | 4.88E-10 | 0.8844 | T | C | -0.0174 | 0.0219 | 0.4266 | 757601 |
| rs8010344 | 0.1513 | 0.0269 | 1.81E-08 | 0.1799 | A | G | -0.0099 | 0.0183 | 0.5882 | 757601 |
| rs8017780 | 0.162 | 0.0251 | 1.14E-10 | 0.7861 | A | C | -0.0209 | 0.0173 | 0.2284 | 757601 |
| rs8052826 | 0.1683 | 0.0254 | 3.40E-11 | 0.7887 | A | G | 0.0047 | 0.018 | 0.7951 | 757601 |
| rs8102624 | 0.5573 | 0.0393 | 1.11E-45 | 0.9228 | A | G | -0.0335 | 0.0273 | 0.221 | 757601 |
| rs8118848 | -0.1881 | 0.0249 | 4.35E-14 | 0.7597 | A | G | 0.0348 | 0.0175 | 0.04713 | 757601 |
| rs848445 | -0.1309 | 0.023 | 1.24E-08 | 0.7146 | T | C | 0.0193 | 0.0162 | 0.2354 | 757601 |
| rs853170 | 0.152 | 0.0233 | 7.13E-11 | 0.2628 | T | C | 4.00E-04 | 0.016 | 0.9799 | 757601 |
| rs916904 | 0.1199 | 0.0217 | 3.33E-08 | 0.6293 | A | G | -0.0118 | 0.0155 | 0.4455 | 757601 |
| rs9291825 | -0.1274 | 0.0206 | 5.91E-10 | 0.5162 | A | G | 0.0128 | 0.0142 | 0.3693 | 757601 |
| rs929581 | 0.1318 | 0.0213 | 5.73E-10 | 0.3544 | T | C | -0.0026 | 0.0148 | 0.86 | 757601 |
| rs9302885 | 0.1208 | 0.0206 | 4.12E-09 | 0.5545 | A | G | 0.0102 | 0.0144 | 0.4786 | 757601 |
| rs9310608 | -0.1673 | 0.0295 | 1.45E-08 | 0.8565 | T | C | -7.00E-04 | 0.0205 | 0.9741 | 757601 |
| rs9337951 | 0.2583 | 0.0227 | 4.24E-30 | 0.6585 | A | G | -0.0277 | 0.0166 | 0.09473 | 757601 |
| rs9340985 | -0.4763 | 0.033 | 4.20E-47 | 0.1095 | T | C | 0.0077 | 0.023 | 0.7376 | 757601 |
| rs9349379 | 0.2677 | 0.0212 | 1.32E-36 | 0.4068 | A | G | -0.0067 | 0.0148 | 0.6496 | 757601 |
| rs9356816 | -0.1292 | 0.0236 | 4.35E-08 | 0.2498 | A | G | -0.0114 | 0.0165 | 0.4872 | 757601 |
| rs9486916 | 0.1842 | 0.0261 | 1.84E-12 | 0.8025 | T | C | -0.0254 | 0.0181 | 0.1595 | 757601 |
| rs949827 | 0.1523 | 0.0218 | 2.63E-12 | 0.3253 | T | C | 0.0078 | 0.0152 | 0.6082 | 757601 |
| rs9532798 | 0.1395 | 0.0241 | 7.62E-09 | 0.2416 | T | C | -0.0044 | 0.0163 | 0.7871 | 757601 |
| rs9549328 | 0.2164 | 0.0247 | 1.77E-18 | 0.7696 | T | C | -0.0049 | 0.0175 | 0.7812 | 757601 |
| rs9608690 | -0.2489 | 0.0409 | 1.15E-09 | 0.9319 | A | G | 0.0492 | 0.0282 | 0.08054 | 757601 |
| rs9661802 | 0.1377 | 0.0218 | 2.66E-10 | 0.3345 | A | C | -0.0339 | 0.0155 | 0.02854 | 757601 |
| rs9747001 | -0.209 | 0.0251 | 8.84E-17 | 0.7917 | A | G | 0.0163 | 0.0175 | 0.3518 | 757601 |
| rs977184 | -0.1947 | 0.0213 | 6.86E-20 | 0.3741 | T | C | -0.0059 | 0.0149 | 0.6922 | 757601 |
| rs9835724 | 0.1626 | 0.0221 | 1.82E-13 | 0.3148 | A | G | 0.015 | 0.0154 | 0.3315 | 757601 |
| rs9835962 | -0.2514 | 0.0392 | 1.40E-10 | 0.9251 | A | C | 0.0113 | 0.0258 | 0.6621 | 757601 |
| rs9839213 | -0.5316 | 0.0279 | 4.03E-81 | 0.8299 | T | C | -0.0112 | 0.0189 | 0.5552 | 757601 |
| rs9860290 | -0.1737 | 0.0252 | 5.22E-12 | 0.7908 | A | G | -0.0266 | 0.0178 | 0.136 | 757601 |
| rs9860302 | -0.1365 | 0.0227 | 1.89E-09 | 0.2809 | A | G | -0.0183 | 0.0157 | 0.2444 | 757601 |
| rs9937815 | -0.1386 | 0.022 | 2.87E-10 | 0.3266 | A | G | -0.0021 | 0.0152 | 0.8909 | 757601 |
| rs999958 | -0.2208 | 0.0205 | 5.69E-27 | 0.5172 | A | C | 0.0014 | 0.0142 | 0.9201 | 757601 |
