# Supplementary material for: Genetically determined blood pressure, antihypertensive medications, and risk of Alzheimer’s disease: a Mendelian randomization study
Source: Alzheimers Res Ther. 2021 Feb 9;13:41. doi: 10.1186/s13195-021-00782-y (PMC7874453; doi:10.1186/s13195-021-00782-y)
Supplement: Supplementary file 6 — Additional file 6. SNPs that fulfilled our selection criteria to be used as proxies for the effects for AHM classes. [file 13195_2021_782_MOESM6_ESM.docx]

**Additional file 6 SNP that fulfilled our selection criteria to be used as proxies for the effects for antihypertensive drug classes**

|  | SNP | Beta_  exposure | SE_  exposure | Pval_  exposure | EAF | Effect_  allele | Other_  allele | Beta_  outcome | SE_  outcome | Pval_  outcome | Gene | Chromosome: position |
| --- | --- | --- | --- | --- | --- | --- | --- | --- | --- | --- | --- | --- |
| **ARB** | rs1797912 | 1.74E-01 | 3.17E-02 | 3.79E-08 | 3.62E-01 | A | C | -1.07E-02 | 1.49E-02 | 4.74E-01 | *PPARG* | chr3:12428740 (GRCh38.p12) |
| **CCB** | rs718448 | 2.06E-01 | 3.46E-02 | 2.43E-09 | 7.43E-01 | T | C | -2.43E-02 | 1.63E-02 | 1.37E-01 | *PDE1A* | chr2:182339642 (GRCh38.p12) |
|  | rs312487 | 2.19E-01 | 3.07E-02 | 9.65E-13 | 5.22E-01 | T | C | -7.00E-03 | 1.45E-02 | 6.29E-01 | *CACNA1D* | chr3:53511595 (GRCh38.p12) |
|  | rs3821843 | 3.37E-01 | 3.35E-02 | 6.56E-24 | 3.19E-01 | A | G | -1.69E-02 | 1.61E-02 | 2.93E-01 | *CACNA1D* | chr3:53523985 (GRCh38.p12) |
|  | rs9311502 | -2.46E-01 | 3.55E-02 | 3.87E-12 | 2.39E-01 | T | C | 4.00E-03 | 1.68E-02 | 8.10E-01 | *CACNA1D* | chr3:53526294 (GRCh38.p12) |
|  | rs1547950 | -2.15E-01 | 3.07E-02 | 2.33E-12 | 4.62E-01 | T | C | 2.22E-02 | 1.48E-02 | 1.32E-01 | *CACNA1D* | chr3:53534256 (GRCh38.p12) |
|  | rs11709630 | 1.93E-01 | 3.20E-02 | 1.61E-09 | 3.63E-01 | T | G | 1.70E-03 | 1.53E-02 | 9.10E-01 | *CACNA1D* | chr3:53543137 (GRCh38.p12) |
|  | rs114987861 | 5.29E-01 | 9.58E-02 | 3.36E-08 | 9.72E-01 | A | G | -5.66E-02 | 4.83E-02 | 2.41E-01 | *CACNA1D* | chr3:53571685 (GRCh38.p12) |
|  | rs113210396 | -4.34E-01 | 7.70E-02 | 1.76E-08 | 9.55E-01 | T | G | 5.64E-02 | 3.81E-02 | 1.39E-01 | *CACNA1D* | chr3:53578300 (GRCh38.p12) |
|  | rs7340705 | -2.43E-01 | 3.22E-02 | 4.87E-14 | 3.27E-01 | T | C | 4.20E-03 | 1.51E-02 | 7.79E-01 | *CACNA1D* | chr3:53700416 (GRCh38.p12) |
|  | rs2633731 | -1.96E-01 | 3.09E-02 | 2.21E-10 | 6.04E-01 | T | C | -2.00E-02 | 1.46E-02 | 1.71E-01 | *CACNA1D* | chr3:53704397 (GRCh38.p12) |
|  | rs10764319 | 2.69E-01 | 3.29E-02 | 2.54E-16 | 6.96E-01 | T | C | 1.90E-03 | 1.56E-02 | 9.04E-01 | *CACNB2* | chr10:18139486 (GRCh38.p12) |
|  | rs34606998 | 2.58E-01 | 3.57E-02 | 4.34E-13 | 7.61E-01 | T | C | -1.57E-02 | 1.71E-02 | 3.59E-01 | *CACNB2* | chr10:18141926 (GRCh38.p12) |
|  | rs11012811 | 3.10E-01 | 3.26E-02 | 2.31E-21 | 6.90E-01 | T | G | -2.19E-02 | 1.54E-02 | 1.53E-01 | *CACNB2* | chr10:18149527 (GRCh38.p12) |
|  | rs1888693 | 3.86E-01 | 3.17E-02 | 4.69E-34 | 6.55E-01 | A | G | -7.50E-03 | 1.50E-02 | 6.16E-01 | *CACNB2* | chr10:18151515 (GRCh38.p12) |
|  | rs17604757 | -5.02E-01 | 6.06E-02 | 1.12E-16 | 6.75E-02 | A | G | 2.60E-03 | 2.86E-02 | 9.27E-01 | *CACNB2* | chr10:18154011 (GRCh38.p12) |
|  | rs12571593 | -4.00E-01 | 5.21E-02 | 1.71E-14 | 9.27E-02 | A | G | 1.80E-02 | 2.47E-02 | 4.67E-01 | *CACNB2* | chr10:18154293 (GRCh38.p12) |
|  | rs17662793 | 2.36E-01 | 3.38E-02 | 2.65E-12 | 2.88E-01 | A | G | -2.48E-02 | 1.60E-02 | 1.21E-01 | *CACNB2* | chr10:18176550 (GRCh38.p12) |
|  | rs2482100 | 3.13E-01 | 4.17E-02 | 5.65E-14 | 8.43E-01 | A | G | -2.11E-02 | 1.96E-02 | 2.81E-01 | *CACNB2* | chr10:18192151 (GRCh38.p12) |
|  | rs61278674 | -3.30E-01 | 5.40E-02 | 1.03E-09 | 9.38E-02 | A | G | -7.70E-03 | 2.83E-02 | 7.84E-01 | *CACNB2* | chr10:18192808 (GRCh38.p12) |
|  | rs4748444 | 1.94E-01 | 3.27E-02 | 3.13E-09 | 3.36E-01 | T | C | -2.37E-02 | 1.56E-02 | 1.30E-01 | *CACNB2* | chr10:18205553 (GRCh38.p12) |
|  | rs1779209 | 2.74E-01 | 3.36E-02 | 4.23E-16 | 7.12E-01 | T | C | -5.00E-03 | 1.57E-02 | 7.53E-01 | *CACNB2* | chr10:18225632 (GRCh38.p12) |
|  | rs1757213 | 3.08E-01 | 5.07E-02 | 1.15E-09 | 8.88E-01 | A | G | -5.90E-03 | 2.68E-02 | 8.26E-01 | *CACNB2* | chr10:18248665 (GRCh38.p12) |
|  | rs10828399 | -1.95E-01 | 3.02E-02 | 1.10E-10 | 4.78E-01 | A | G | -4.00E-04 | 1.42E-02 | 9.79E-01 | *CACNB2* | chr10:18265039 (GRCh38.p12) |
|  | rs17610275 | 3.87E-01 | 6.13E-02 | 2.87E-10 | 7.34E-02 | T | G | -5.69E-02 | 2.83E-02 | 4.42E-02 | *CACNB2* | chr10:18332701 (GRCh38.p12) |
|  | rs10828542 | 1.82E-01 | 3.11E-02 | 5.18E-09 | 3.86E-01 | A | G | -1.76E-02 | 1.49E-02 | 2.37E-01 | *CACNB2* | chr10:18338356 (GRCh38.p12) |
|  | rs10741039 | 1.72E-01 | 3.01E-02 | 1.22E-08 | 4.76E-01 | A | C | -9.60E-03 | 1.44E-02 | 5.05E-01 | *CACNB2* | chr10:18370887 (GRCh38.p12) |
|  | rs11013938 | -3.27E-01 | 3.50E-02 | 1.17E-20 | 7.45E-01 | C | G | 2.79E-02 | 1.66E-02 | 9.22E-02 | *CACNB2* | chr10:18380342 (GRCh38.p12) |
|  | rs35241357 | -3.08E-01 | 3.17E-02 | 2.64E-22 | 3.52E-01 | A | G | 1.89E-02 | 1.50E-02 | 2.07E-01 | *CACNB2* | chr10:18397336 (GRCh38.p12) |
|  | rs112133583 | -5.55E-01 | 9.73E-02 | 1.18E-08 | 9.70E-01 | T | C | -7.05E-02 | 5.47E-02 | 1.97E-01 | *CACNB2* | chr10:18406752 (GRCh38.p12) |
|  | rs10828662 | -2.88E-01 | 3.04E-02 | 2.54E-21 | 4.41E-01 | T | C | 2.30E-03 | 1.44E-02 | 8.71E-01 | *CACNB2* | chr10:18414168 (GRCh38.p12) |
|  | rs982003 | -2.41E-01 | 3.51E-02 | 6.21E-12 | 2.43E-01 | T | C | -6.70E-03 | 1.70E-02 | 6.91E-01 | *CACNB2* | chr10:18418367 (GRCh38.p12) |
|  | rs1325990 | -3.87E-01 | 3.02E-02 | 1.09E-37 | 5.30E-01 | A | G | 1.22E-02 | 1.42E-02 | 3.91E-01 | *CACNB2* | chr10:18418423 (GRCh38.p12) |
|  | rs11014170 | -6.70E-01 | 1.15E-01 | 5.61E-09 | 9.79E-01 | A | G | 3.19E-02 | 5.66E-02 | 5.73E-01 | *CACNB2* | chr10:18422062 (GRCh38.p12) |
|  | rs67214975 | -4.14E-01 | 3.07E-02 | 1.42E-41 | 5.44E-01 | A | C | 1.56E-02 | 1.48E-02 | 2.94E-01 | *CACNB2* | chr10:18438322 (GRCh38.p12) |
|  | rs7923191 | -3.69E-01 | 3.76E-02 | 1.10E-22 | 2.08E-01 | A | G | 1.88E-02 | 1.78E-02 | 2.92E-01 | *CACNB2* | chr10:18438972 (GRCh38.p12) |
|  | rs12258967 | 6.33E-01 | 3.37E-02 | 1.08E-78 | 2.95E-01 | C | G | -1.68E-02 | 1.64E-02 | 3.05E-01 | *CACNB2* | chr10:18439030 (GRCh38.p12) |
|  | rs72786098 | -5.03E-01 | 8.83E-02 | 1.18E-08 | 9.68E-01 | A | G | 3.40E-03 | 4.52E-02 | 9.41E-01 | *CACNB2* | chr10:18440926 (GRCh38.p12) |
|  | rs116936375 | -5.74E-01 | 8.10E-02 | 1.40E-12 | 9.60E-01 | A | G | 1.60E-03 | 3.92E-02 | 9.68E-01 | *CACNB2* | chr10:18448206 (GRCh38.p12) |
|  | rs1998822 | -1.96E-01 | 3.43E-02 | 1.15E-08 | 2.77E-01 | A | G | 1.46E-02 | 1.61E-02 | 3.65E-01 | *CACNB2* | chr10:18466735 (GRCh38.p12) |
|  | rs10828749 | -3.66E-01 | 3.09E-02 | 2.27E-32 | 5.88E-01 | A | G | 2.24E-02 | 1.45E-02 | 1.24E-01 | *CACNB2* | chr10:18467952 (GRCh38.p12) |
|  | rs7076247 | 2.56E-01 | 3.09E-02 | 1.33E-16 | 6.11E-01 | T | C | -1.80E-02 | 1.45E-02 | 2.16E-01 | *CACNB2* | chr10:18470700 (GRCh38.p12) |
|  | rs4748472 | 3.16E-01 | 3.19E-02 | 4.04E-23 | 3.44E-01 | T | C | -2.88E-02 | 1.49E-02 | 5.38E-02 | *CACNB2* | chr10:18487268 (GRCh38.p12) |
|  | rs12416030 | -2.09E-01 | 3.81E-02 | 4.32E-08 | 2.03E-01 | T | C | 1.20E-02 | 1.81E-02 | 5.07E-01 | *CACNB2* | chr10:18500146 (GRCh38.p12) |
|  | rs12416052 | 1.99E-01 | 3.11E-02 | 1.59E-10 | 4.05E-01 | T | C | -3.10E-02 | 1.46E-02 | 3.38E-02 | *CACNB2* | chr10:18500338 (GRCh38.p12) |
|  | rs4748476 | 2.17E-01 | 3.65E-02 | 2.89E-09 | 2.23E-01 | T | C | -4.70E-03 | 1.72E-02 | 7.84E-01 | *CACNB2* | chr10:18503946 (GRCh38.p12) |
|  | rs2239046 | 2.08E-01 | 3.22E-02 | 9.58E-11 | 3.18E-01 | A | G | 4.10E-03 | 1.55E-02 | 7.90E-01 | *CACNA1C* | chr12:2325253 (GRCh38.p12) |
|  | rs714277 | 1.99E-01 | 3.33E-02 | 2.38E-09 | 7.17E-01 | T | C | 4.90E-03 | 1.60E-02 | 7.59E-01 | *CACNA1C* | chr12:2405104 (GRCh38.p12) |
| **Thiazides** | rs1262894 | 3.47E-01 | 6.10E-02 | 1.29E-08 | 7.64E-02 | A | C | 4.03E-02 | 3.27E-02 | 2.18E-01 | *SCNN1D* | chr1:1281631 (GRCh38.p12) |
|  | rs33996239 | -3.66E-01 | 6.62E-02 | 3.39E-08 | 9.40E-01 | T | C | -3.57E-02 | 3.13E-02 | 2.54E-01 | *ADORA1* | chr1:203140673 (GRCh38.p12) |
|  | rs4836365 | 3.48E-01 | 4.78E-02 | 3.15E-13 | 8.87E-01 | A | C | -1.43E-02 | 2.31E-02 | 5.37E-01 | *SLC12A2* | chr5:128141078 (GRCh38.p12) |
|  | rs59484271 | 2.41E-01 | 3.73E-02 | 1.09E-10 | 7.87E-01 | T | C | -1.40E-03 | 1.77E-02 | 9.35E-01 | *SLC12A2* | chr5:128168089 (GRCh38.p12) |
|  | rs3101725 | -1.98E-01 | 3.56E-02 | 2.75E-08 | 7.58E-01 | T | C | 1.32E-02 | 1.68E-02 | 4.32E-01 | *SLC12A2* | chr5:128188326 (GRCh38.p12) |
| **BB** | rs12540183 | 1.95E-01 | 3.12E-02 | 4.17E-10 | 6.16E-01 | T | C | -1.34E-02 | 1.47E-02 | 3.63E-01 | *KCNH2* | chr7:150967053 (GRCh38.p12) |

| **AHMs**/SNP | Beta_  exposure | SE_  exposure | Pval_  exposure | EAF | Effect_  allele | Other_  allele | Beta_  outcome | SE_  outcome | Pval_  outcome | Gene | chromosome: position | F | R^2^ |
| --- | --- | --- | --- | --- | --- | --- | --- | --- | --- | --- | --- | --- | --- |
| rs1262894 | 0.3469 | 0.061 | 1.29E-08 | 0.0764 | A | C | 0.0403 | 0.0327 | 0.2178 | *SCNN1D* | chr1:1281631 (GRCh38.p12) | 32.35 | 4.27E-05 |
| rs33996239 | -0.3655 | 0.0662 | 3.39E-08 | 0.9399 | T | C | -0.0357 | 0.0313 | 0.254 | *ADORA1* | chr1:203140673 (GRCh38.p12) | 30.47 | 4.02E-05 |
| rs718448 | 0.2063 | 0.0346 | 2.43E-09 | 0.7428 | T | C | -0.0243 | 0.0163 | 0.1373 | *PDE1A* | chr2:182339642 (GRCh38.p12) | 35.59 | 4.70E-05 |
| rs1797912 | 0.1742 | 0.0317 | 3.79E-08 | 0.3618 | A | C | -0.0107 | 0.0149 | 0.4736 | *PPARG* | chr3:12428740 (GRCh38.p12) | 30.25 | 3.99E-05 |
| rs312487 | 0.2194 | 0.0307 | 9.65E-13 | 0.5217 | T | C | -0.007 | 0.0145 | 0.6285 | *CACNA1D* | chr3:53511595 (GRCh38.p12) | 50.91 | 6.72E-05 |
| rs3821843 | 0.3373 | 0.0335 | 6.56E-24 | 0.3192 | A | G | -0.0169 | 0.0161 | 0.2931 | *CACNA1D* | chr3:53523985 (GRCh38.p12) | 101.67 | 1.34E-04 |
| rs9311502 | -0.2463 | 0.0355 | 3.87E-12 | 0.2391 | T | C | 0.004 | 0.0168 | 0.8104 | *CACNA1D* | chr3:53526294 (GRCh38.p12) | 48.19 | 6.36E-05 |
| rs1547950 | -0.2151 | 0.0307 | 2.33E-12 | 0.4623 | T | C | 0.0222 | 0.0148 | 0.132 | *CACNA1D* | chr3:53534256 (GRCh38.p12) | 49.18 | 6.49E-05 |
| rs11709630 | 0.1931 | 0.032 | 1.61E-09 | 0.3627 | T | G | 0.0017 | 0.0153 | 0.9098 | *CACNA1D* | chr3:53543137 (GRCh38.p12) | 36.4 | 4.80E-05 |
| rs114987861 | 0.5289 | 0.0958 | 3.36E-08 | 0.9716 | A | G | -0.0566 | 0.0483 | 0.2408 | *CACNA1D* | chr3:53571685 (GRCh38.p12) | 30.49 | 4.02E-05 |
| rs113210396 | -0.4338 | 0.077 | 1.76E-08 | 0.9549 | T | G | 0.0564 | 0.0381 | 0.1392 | *CACNA1D* | chr3:53578300 (GRCh38.p12) | 31.74 | 4.19E-05 |
| rs7340705 | -0.2425 | 0.0322 | 4.87E-14 | 0.3268 | T | C | 0.0042 | 0.0151 | 0.7786 | *CACNA1D* | chr3:53700416 (GRCh38.p12) | 56.78 | 7.49E-05 |
| rs2633731 | -0.1963 | 0.0309 | 2.21E-10 | 0.6038 | T | C | -0.02 | 0.0146 | 0.1709 | *CACNA1D* | chr3:53704397 (GRCh38.p12) | 40.27 | 5.32E-05 |
| rs4836365 | 0.3483 | 0.0478 | 3.15E-13 | 0.8871 | A | C | -0.0143 | 0.0231 | 0.5369 | *SLC12A2* | chr5:128141078 (GRCh38.p12) | 53.11 | 7.01E-05 |
| rs59484271 | 0.241 | 0.0373 | 1.09E-10 | 0.787 | T | C | -0.0014 | 0.0177 | 0.9354 | *SLC12A2* | chr5:128168089 (GRCh38.p12) | 41.65 | 5.50E-05 |
| rs3101725 | -0.1979 | 0.0356 | 2.75E-08 | 0.7583 | T | C | 0.0132 | 0.0168 | 0.4322 | *SLC12A2* | chr5:128188326 (GRCh38.p12) | 30.88 | 4.08E-05 |
| rs12540183 | 0.1951 | 0.0312 | 4.17E-10 | 0.6157 | T | C | -0.0134 | 0.0147 | 0.363 | *KCNH2* | chr7:150967053 (GRCh38.p12) | 39.03 | 5.15E-05 |
| rs10764319 | 0.2693 | 0.0329 | 2.54E-16 | 0.6956 | T | C | 0.0019 | 0.0156 | 0.904 | *CACNB2* | chr10:18139486 (GRCh38.p12) | 67.13 | 8.86E-05 |
| rs34606998 | 0.2583 | 0.0357 | 4.34E-13 | 0.7606 | T | C | -0.0157 | 0.0171 | 0.3585 | *CACNB2* | chr10:18141926 (GRCh38.p12) | 52.48 | 6.93E-05 |
| rs11012811 | 0.3095 | 0.0326 | 2.31E-21 | 0.69 | T | G | -0.0219 | 0.0154 | 0.1531 | *CACNB2* | chr10:18149527 (GRCh38.p12) | 90.06 | 1.19E-04 |
| rs1888693 | 0.3858 | 0.0317 | 4.69E-34 | 0.6551 | A | G | -0.0075 | 0.015 | 0.6164 | *CACNB2* | chr10:18151515 (GRCh38.p12) | 148.02 | 1.95E-04 |
| rs17604757 | -0.5022 | 0.0606 | 1.12E-16 | 0.0675 | A | G | 0.0026 | 0.0286 | 0.9268 | *CACNB2* | chr10:18154011 (GRCh38.p12) | 68.75 | 9.07E-05 |
| rs12571593 | -0.3996 | 0.0521 | 1.71E-14 | 0.0927 | A | G | 0.018 | 0.0247 | 0.4674 | *CACNB2* | chr10:18154293 (GRCh38.p12) | 58.84 | 7.77E-05 |
| rs17662793 | 0.2363 | 0.0338 | 2.65E-12 | 0.2876 | A | G | -0.0248 | 0.016 | 0.1211 | *CACNB2* | chr10:18176550 (GRCh38.p12) | 48.93 | 6.46E-05 |
| rs2482100 | 0.3134 | 0.0417 | 5.65E-14 | 0.8428 | A | G | -0.0211 | 0.0196 | 0.2811 | *CACNB2* | chr10:18192151 (GRCh38.p12) | 56.49 | 7.46E-05 |
| rs61278674 | -0.3298 | 0.054 | 1.03E-09 | 0.0938 | A | G | -0.0077 | 0.0283 | 0.7841 | *CACNB2* | chr10:18192808 (GRCh38.p12) | 37.27 | 4.92E-05 |
| rs4748444 | 0.1939 | 0.0327 | 3.13E-09 | 0.3363 | T | C | -0.0237 | 0.0156 | 0.1296 | *CACNB2* | chr10:18205553 (GRCh38.p12) | 35.1 | 4.63E-05 |
| rs1779209 | 0.2736 | 0.0336 | 4.23E-16 | 0.7124 | T | C | -0.005 | 0.0157 | 0.7528 | *CACNB2* | chr10:18225632 (GRCh38.p12) | 66.13 | 8.73E-05 |
| rs1757213 | 0.3084 | 0.0507 | 1.15E-09 | 0.888 | A | G | -0.0059 | 0.0268 | 0.8264 | *CACNB2* | chr10:18248665 (GRCh38.p12) | 37.05 | 4.89E-05 |
| rs10828399 | -0.1947 | 0.0302 | 1.10E-10 | 0.4782 | A | G | -4.00E-04 | 0.0142 | 0.9793 | *CACNB2* | chr10:18265039 (GRCh38.p12) | 41.64 | 5.50E-05 |
| rs17610275 | 0.3868 | 0.0613 | 2.87E-10 | 0.0734 | T | G | -0.0569 | 0.0283 | 0.0442 | *CACNB2* | chr10:18332701 (GRCh38.p12) | 39.76 | 5.25E-05 |
| rs10828542 | 0.1817 | 0.0311 | 5.18E-09 | 0.3863 | A | G | -0.0176 | 0.0149 | 0.2371 | *CACNB2* | chr10:18338356 (GRCh38.p12) | 34.12 | 4.50E-05 |
| rs10741039 | 0.1716 | 0.0301 | 1.22E-08 | 0.4761 | A | C | -0.0096 | 0.0144 | 0.5047 | *CACNB2* | chr10:18370887 (GRCh38.p12) | 32.45 | 4.28E-05 |
| rs35241357 | -0.3077 | 0.0317 | 2.64E-22 | 0.3522 | A | G | 0.0189 | 0.015 | 0.2069 | *CACNB2* | chr10:18397336 (GRCh38.p12) | 94.35 | 1.25E-04 |
| rs112133583 | -0.5546 | 0.0973 | 1.18E-08 | 0.9701 | T | C | -0.0705 | 0.0547 | 0.1969 | *CACNB2* | chr10:18406752 (GRCh38.p12) | 32.52 | 4.29E-05 |
| rs10828662 | -0.2879 | 0.0304 | 2.54E-21 | 0.4414 | T | C | 0.0023 | 0.0144 | 0.8705 | *CACNB2* | chr10:18414168 (GRCh38.p12) | 89.87 | 1.19E-04 |
| rs982003 | -0.2414 | 0.0351 | 6.21E-12 | 0.2432 | T | C | -0.0067 | 0.017 | 0.6913 | *CACNB2* | chr10:18418367 (GRCh38.p12) | 47.26 | 6.24E-05 |
| rs1325990 | -0.3873 | 0.0302 | 1.09E-37 | 0.5297 | A | G | 0.0122 | 0.0142 | 0.3912 | *CACNB2* | chr10:18418423 (GRCh38.p12) | 164.65 | 2.17E-04 |
| rs11014170 | -0.6701 | 0.115 | 5.61E-09 | 0.9794 | A | G | 0.0319 | 0.0566 | 0.5727 | *CACNB2* | chr10:18422062 (GRCh38.p12) | 33.97 | 4.48E-05 |
| rs67214975 | -0.4144 | 0.0307 | 1.42E-41 | 0.5437 | A | C | 0.0156 | 0.0148 | 0.2944 | *CACNB2* | chr10:18438322 (GRCh38.p12) | 182.44 | 2.41E-04 |
| rs7923191 | -0.369 | 0.0376 | 1.10E-22 | 0.2082 | A | G | 0.0188 | 0.0178 | 0.2915 | *CACNB2* | chr10:18438972 (GRCh38.p12) | 96.09 | 1.27E-04 |
| rs72786098 | -0.5033 | 0.0883 | 1.18E-08 | 0.9678 | A | G | 0.0034 | 0.0452 | 0.9407 | *CACNB2* | chr10:18440926 (GRCh38.p12) | 32.52 | 4.29E-05 |
| rs116936375 | -0.5739 | 0.081 | 1.40E-12 | 0.9595 | A | G | 0.0016 | 0.0392 | 0.968 | *CACNB2* | chr10:18448206 (GRCh38.p12) | 50.18 | 6.62E-05 |
| rs1998822 | -0.1958 | 0.0343 | 1.15E-08 | 0.2766 | A | G | 0.0146 | 0.0161 | 0.365 | *CACNB2* | chr10:18466735 (GRCh38.p12) | 32.57 | 4.30E-05 |
| rs10828749 | -0.3658 | 0.0309 | 2.27E-32 | 0.588 | A | G | 0.0224 | 0.0145 | 0.1241 | *CACNB2* | chr10:18467952 (GRCh38.p12) | 140.32 | 1.85E-04 |
| rs7076247 | 0.2557 | 0.0309 | 1.33E-16 | 0.6114 | T | C | -0.018 | 0.0145 | 0.2155 | *CACNB2* | chr10:18470700 (GRCh38.p12) | 68.41 | 9.03E-05 |
| rs4748472 | 0.3161 | 0.0319 | 4.04E-23 | 0.3442 | T | C | -0.0288 | 0.0149 | 0.0538 | *CACNB2* | chr10:18487268 (GRCh38.p12) | 98.07 | 1.29E-04 |
| rs12416030 | -0.2088 | 0.0381 | 4.32E-08 | 0.2031 | T | C | 0.012 | 0.0181 | 0.5065 | *CACNB2* | chr10:18500146 (GRCh38.p12) | 30 | 3.96E-05 |
| rs12416052 | 0.1987 | 0.0311 | 1.59E-10 | 0.4053 | T | C | -0.031 | 0.0146 | 0.03375 | *CACNB2* | chr10:18500338 (GRCh38.p12) | 40.91 | 5.40E-05 |
| rs4748476 | 0.2166 | 0.0365 | 2.89E-09 | 0.2229 | T | C | -0.0047 | 0.0172 | 0.7842 | *CACNB2* | chr10:18503946 (GRCh38.p12) | 35.26 | 4.65E-05 |
| rs2239046 | 0.2082 | 0.0322 | 9.58E-11 | 0.3183 | A | G | 0.0041 | 0.0155 | 0.7902 | *CACNA1C* | chr12:2325253 (GRCh38.p12) | 41.91 | 5.53E-05 |
| rs714277 | 0.1986 | 0.0333 | 2.38E-09 | 0.7166 | T | C | 0.0049 | 0.016 | 0.7593 | *CACNA1C* | chr12:2405104 (GRCh38.p12) | 35.63 | 4.70E-05 |
