# Supplementary figures and images for: Genetically determined blood pressure, antihypertensive medications, and risk of Alzheimer’s disease: a Mendelian randomization study
Source: Alzheimers Res Ther. 2021 Feb 9;13:41. doi: 10.1186/s13195-021-00782-y (PMC7874453; doi:10.1186/s13195-021-00782-y)

**Additional file 7 Leave-one-out plots**

**AHMs**

**
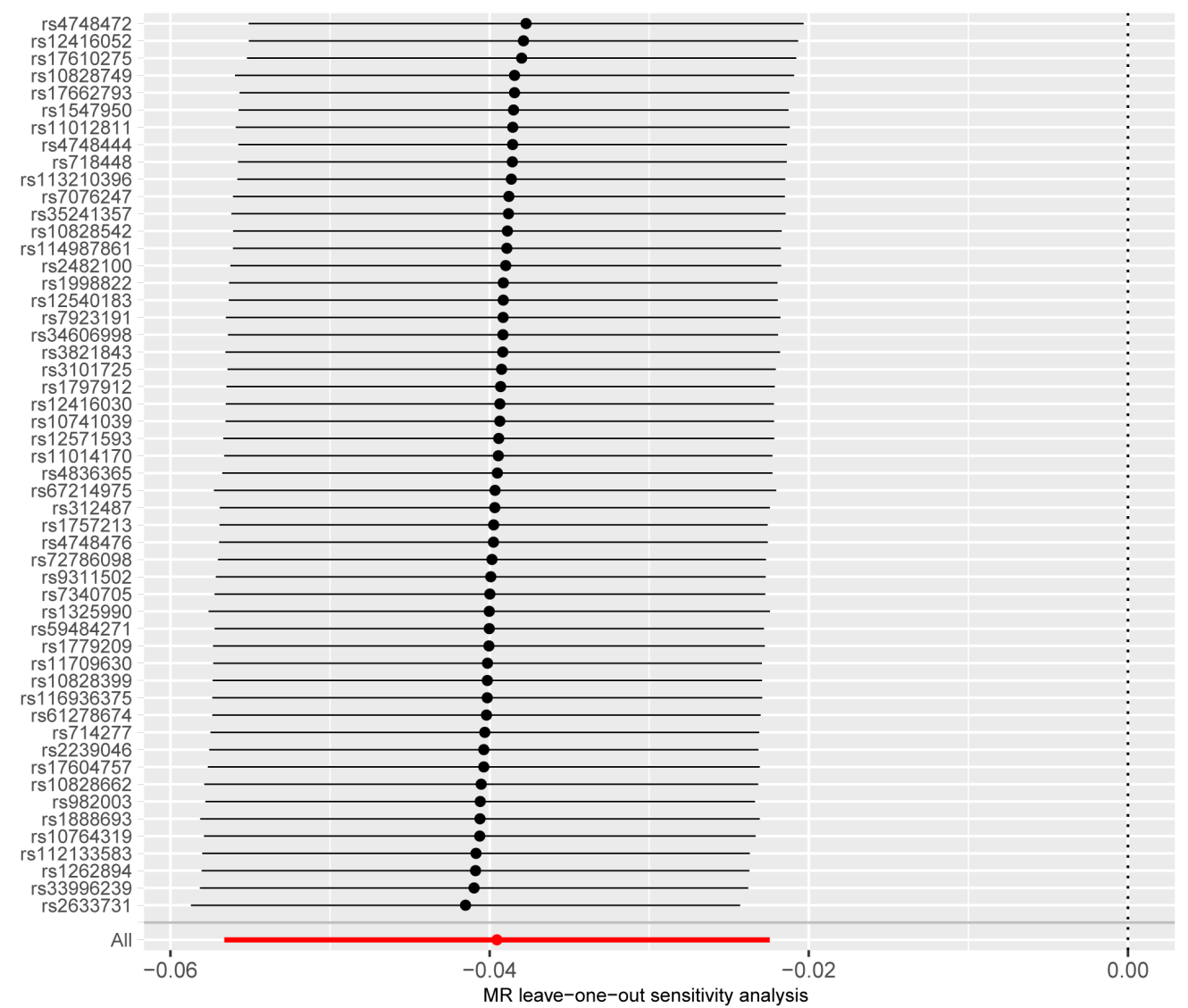
**

**CCB**

**
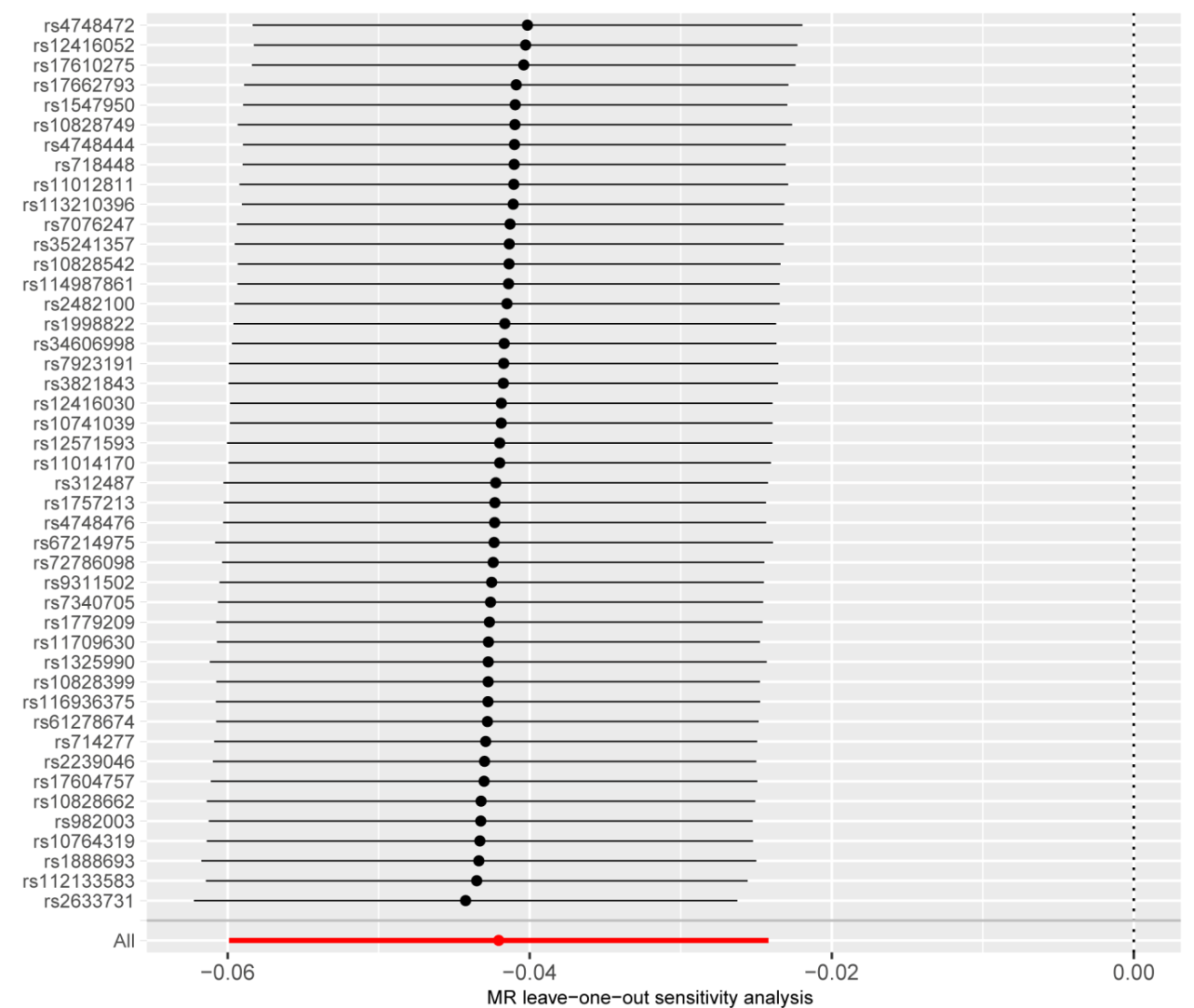
**

**Thiazides**

**
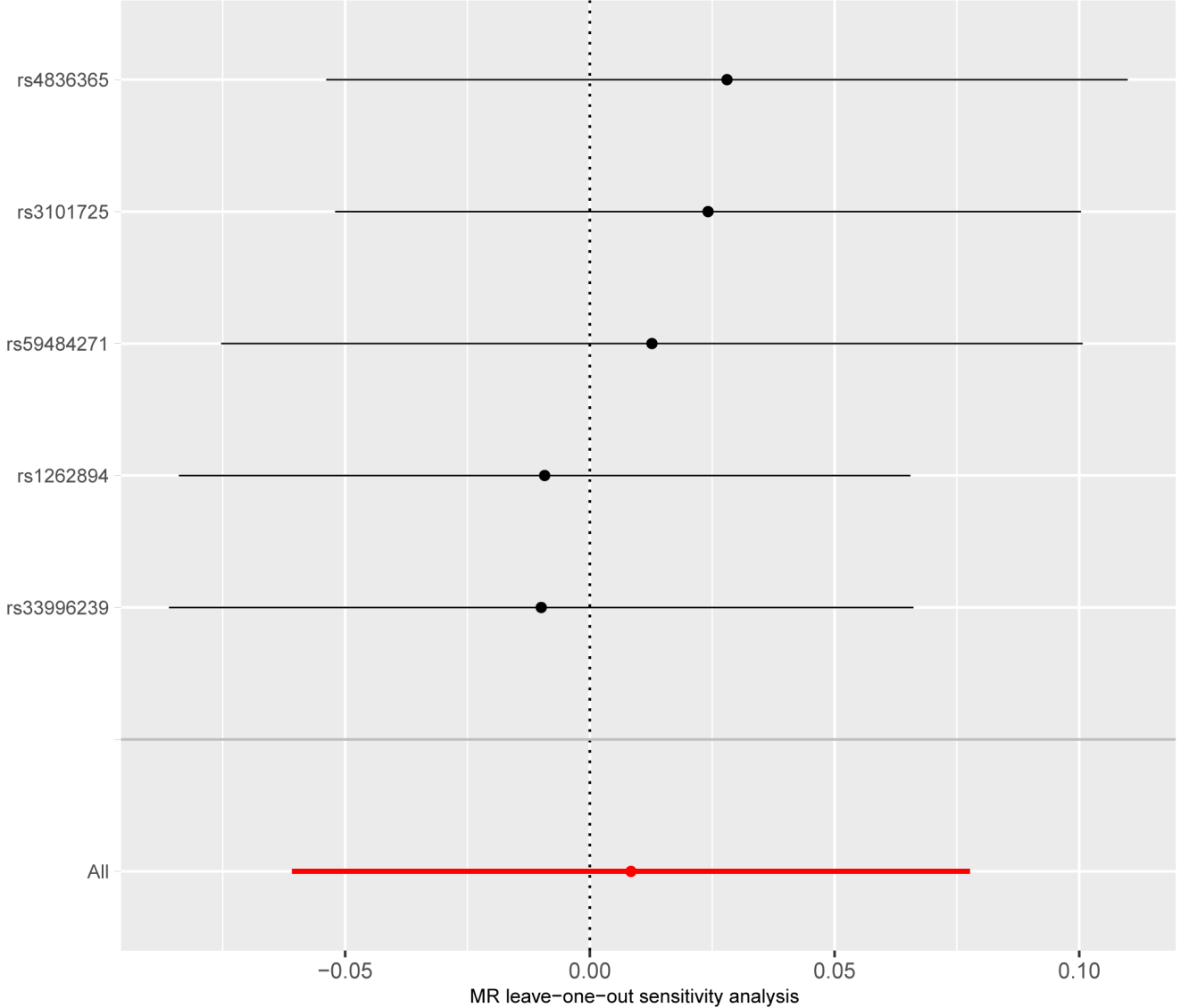
**

Supplement: Supplementary file 7 — Additional file 7. Leave-one-out plots. [file 13195_2021_782_MOESM7_ESM.docx]
