# Supplementary material for: Genetically determined blood pressure, antihypertensive medications, and risk of Alzheimer’s disease: a Mendelian randomization study
Source: Alzheimers Res Ther. 2021 Feb 9;13:41. doi: 10.1186/s13195-021-00782-y (PMC7874453; doi:10.1186/s13195-021-00782-y)
Supplement: Supplementary file 8 — Additional file 8. MR results for the casual relationships between AHMs and AD using a LD R2 < 0.1. [file 13195_2021_782_MOESM8_ESM.docx]

**Additional file 8 MR results for the casual relationships between AHMs and AD using a LD R^2^<0.1**

|  | **OR (95% CI)** | **P value** | **No. of SNPs** |
| --- | --- | --- | --- |
| ***AHMs*** | |  |  |
| **IVW** | **0.961 (0.936-0.987)** | **0.003** | **27** |
| MR Egger | 0.965 (0.898-1.038) | 0.347 | 27 |
| Weighted median | 0.967 (0.932-1.004) | 0.078 | 27 |
| Simple mode | 0.975 (0.912-1.043) | 0.471 | 27 |
| Weighted mode | 0.968 (0.921-1.018) | 0.220 | 27 |
| ***ARB*** | |  |  |
| Wald ratio | 0.940 (0.795-1.112) | 0.473 | 1 |
| ***BB*** |  |  |  |
| Wald ratio | 0.937 (0.800-1.099) | 0.426 | 1 |
| ***CCB*** |  |  |  |
| **IVW** | **0.955 (0.927-0.983)** | **0.002** | **21** |
| MR Egger | 0.945 (0.875-1.020) | 0.164 | 21 |
| Weighted median | 0.967 (0.930-1.006) | 0.099 | 21 |
| Simple mode | 0.982 (0.917-1.051) | 0.604 | 21 |
| Weighted mode | 0.969 (0.919-1.022) | 0.259 | 21 |
| ***Thiazides*** | |  |  |
| IVW | 1.028 (0.937-1.128) | 0.565 | 4 |
| MR Egger | 1.403 (1.006-1.956) | 0.184 | 4 |
| Weighted median | 1.044 (0.941-1.158) | 0.417 | 4 |
| Simple mode | 1.097 (0.939-1.282) | 0.327 | 4 |
| Weighted mode | 1.083 (0.938-1.249) | 0.357 | 4 |

Bold fonts represent significant results.

Abbreviations: MR, Mendelian randomization; AHMs, antihypertensive medications; AD, Alzheimer’s disease; OR, odds ratio; CI, confidence interval; SNP, Single nucleotide polymorphism; IVW, Inverse variance weighted; ARB, angiotensin receptor blockers; BB, β-blockers; CCB, calcium channel blocker.
