# Supplementary material for: Genetically determined blood pressure, antihypertensive medications, and risk of Alzheimer’s disease: a Mendelian randomization study
Source: Alzheimers Res Ther. 2021 Feb 9;13:41. doi: 10.1186/s13195-021-00782-y (PMC7874453; doi:10.1186/s13195-021-00782-y)
Supplement: Supplementary file 9 — Additional file 9. MR results for the casual relationships between AHMs and AD using a LD R2 < 0.001. [file 13195_2021_782_MOESM9_ESM.docx]

**Additional file 9 MR results for the casual relationships between AHMs and AD using a LD R^2^<0.001**

|  | **OR (95% CI)** | **P value** | **No. of SNPs** |
| --- | --- | --- | --- |
| ***AHMs*** | |  |  |
| IVW | 0.974 **(**0.933**-**1.015**)** | 0.209 | 9 |
| MR Egger | 0.989 (0.845-1.133) | 0.884 | 9 |
| Weighted median | 0.951 (0.898-1.004) | 0.063 | 9 |
| Simple mode | 0.949 (0.880-1.018) | 0.177 | 9 |
| Weighted mode | 0.950 (0.889-1.012) | 0.142 | 9 |
| ***ARB*** | |  |  |
| Wald ratio | 0.940 (0.795-1.112) | 0.473 | 1 |
| ***BB*** |  |  |  |
| Wald ratio | 0.934 (0.805-1.082) | 0.362 | 1 |
| ***CCB*** |  |  |  |
| IVW | 0.963 **(**0.913**-**1.014**)** | 0.144 | 4 |
| MR Egger | 0.872 (0.658-1.086) | 0.337 | 4 |
| Weighted median | 0.951 (0.891-1.010) | 0.096 | 4 |
| Simple mode | 0.953 (0.880-1.027) | 0.293 | 4 |
| Weighted mode | 0.951 (0.883-1.019) | 0.245 | 4 |
| ***Thiazides*** | |  |  |
| IVW | 1.036 (0.936-1.148) | 0.492 | 3 |
| MR Egger | 4.899 (0.016-1546.898) | 0.684 | 3 |
| Weighted median | 1.053 (0.948-1.170) | 0.335 | 3 |
| Simple mode | 1.113 (0.947-1.307) | 0.322 | 3 |
| Weighted mode | 0.960 (0.854-1.079) | 0.562 | 3 |

Abbreviations: MR, Mendelian randomization; AHMs, antihypertensive medications; AD, Alzheimer’s disease; OR, odds ratio; CI, confidence interval; SNP, Single nucleotide polymorphism; IVW, Inverse variance weighted; ARB, angiotensin receptor blockers; BB, β-blockers; CCB, calcium channel blocker.
